# Cranial form differences in goats by breed and domestic status

**DOI:** 10.1038/s41598-023-50357-0

**Published:** 2024-01-09

**Authors:** A. M. Balcarcel, M. Geiger, M. R. Sánchez-Villagra

**Affiliations:** 1 Department of Paleontology, University of Zurich, Karl-Schmid-Str. 4, 8006 Zurich, Switzerland; 2 Naturmuseum St.Gallen, Rorschacher Strasse 263, 9016 St.Gallen, Switzerland

**Keywords:** Evolution, Zoology

## Abstract

Domestic goats (*Capra hircus*) are globally represented by over 300 breeds, making them a useful model for investigating patterns of morphological change related to domestication. However, they have been little studied, likely due to their poor representation in museum collections and the difficulty in obtaining truly wild goat (*Capra aegagrus*, the bezoar) samples. Similar studies on other species reveal that domestication correlates with craniofacial alterations in domestics, which are non-uniform and often species-specific. Here, we use three-dimensional geometric morphometric methods (3DGMM) to describe and quantify cranial shape variation in wild (n = 21) versus domestic (n = 54) goats. We find that mean cranial shapes differ significantly between wild and domestic goats as well as between certain breeds. The detected differences are lower in magnitude than those reported for other domestic groups, possibly explained by the fewer directions of artificial selection in goat breeding, and their low global genetic diversity compared to other livestock. We also find tooth-row length reduction in the domestics, suggestive of rostral shortening—a prediction of the “domestication syndrome” (DS). The goat model thus expands the array—and combinations of—morphological changes observed under domestication, notably detecting alterations to the calvarium form which could be related to the ~ 15% brain size reduction previously reported for domestic compared to wild goats. The global success of domestic goats is due more to their ability to survive in a variety of harsh environments than to systematized human management. Nonetheless, their domestication has resulted in a clear disruption from the wild cranial form, suggesting that even low-intensity selection can lead to significant morphological changes under domestication.

## Introduction

Phenotypic differences between wild and domestic animals have been studied since the early nineteenth century^1,2^. Evidence of these differences continues to accumulate as more domestic taxa are investigated, yet efforts to assemble a universal set of morphological domestication traits have faltered^3–5^. The broad spectrum of phenotypic changes observed across different domesticated groups includes changes in skin/coat color, body form, reproductive cycles, and behavior^5–11^. Certain skull form changes also occur with remarkable frequency and in distantly related taxa^12–17^, raising the question of potentially common mechanisms^4,18, 19^. A popular explanation for these variations is the Neural Crest Hypothesis (NCH)^19^, that argues selection for tameness triggers developmental changes in neural-crest-derived tissues—including facial bones of the skull—resulting in domesticated populations exhibiting common patterns of phenotypic changes. This hypothesis has benefitted from recent genetic, endocrinal, and developmental insights^20–23^, but is still contested^18,24^. More broadly, investigations of domestication-related skull form changes remain pivotal to understanding patterns of morphological evolution in short time scales.

Goats (*Capra hircus*) were among the first domesticated animals, their domestication process having started approximately ten and a half thousand years ago^8,25–27^. The Middle Eastern species *Capra aegagrus* (the bezoar) is currently regarded as domesticated goats’ closest wild relative^28–30^. Today, the global domestic goat population is estimated to exceed one billion individuals^31^, including more than 300 different breeds^29^. Domestic goats’ wide diversity and distribution has been attributed to a natural resistance to harsh environments, low nutritional requirements, and ease of management^32^. As a result, goats are particularly important to human subsistence in many areas^6,32, 33^, as multi-purpose producers of dairy products, meat, wool and sinew. In some regions, particularly in Europe, most breeds are highly specialized for either dairy or meat production^34^.

Accounts of goat phenotypic diversity have mainly addressed integumentary differences, body size variation, and to a lesser degree, variation in horn shapes^6,35–37^. For example, outer ear shape can vary significantly, from the small nub-like ears of the La Mancha goat to the large floppy ears of the English Nubian; skin coloration can vary much within breeds, but the Boer goat is distinguished by a consistent pattern of a white body with a reddish brown head; and fiber texture can vary from the short fine hair of the Swiss Saanen to the coveted mohair of the Angora^35^. Given this diversity, goats provide a good model for testing patterns of morphological change. However, comparisons of skeletal variation between wild and domestic goats are rare, likely due to the difficulty in obtaining samples of the wild population which, like many breed exemplars, are rare in natural history collections. Previous studies have employed non-geometric morphometrics, and reported variation in post-cranial skeletal proportions^37–39^; and one recent study reported endocranial volume reduction of ~ 15.5% in domestic goats sampled (n = 41) compared to the wild bezoar (n = 23)^40^. However, no previous study has addressed the question of whether and how goat skull form changes under domestication.

Modern methods of three-dimensional geometric morphometrics (3DGMM) have significantly improved investigations of skull form evolution, as they allow higher-resolution visualizations of form and shape. Such approaches facilitate the description and quantification of skull form changes at higher levels of geometric resolution than were possible using non-geometric methods and have been recently applied to domestication models in Carnivora, Artiodactyla, Perissodactyla and Lagomorpha. Results have been mixed with respect to the “domestication syndrome” (DS) hypothesis, which claims domestic populations trend toward (a) rostral shortening and (b) reduction of tooth size or tooth row length^5,19^. In fact, craniofacial changes detected in these analyses vary in manner, direction, and magnitude, depending on the taxon^4^.

The primary goals of this study were to use 3DGMM to describe and compare cranial form variation between wild and domestic goats, and test if their mean cranial forms differ significantly. In particular, we test for two specific morphological changes: (a) rostral shortening and (b) reduction of tooth row length in the domestic sample compared to the wild, as predicted by the DS^19^. We also test for generalized morphological changes across domestics by sampling 17 domestic goat breeds/populations from Europe, African and South America. Lastly, we investigate differences in cranial form variation among breeds, as such variation has been recently observed in domesticated cattle^41^, and test for distinctions in mean cranial form by breed. Although we sampled many populations our dataset is not comprehensive of global goat breed diversity^35^, but it is the first exploration of cranial form diversity in goats using 3DGMM, thus providing a robust database that future studies may enrich.

Horn form varies significantly across Artiodactyla, particularly in the Caprini clade to which goats belong^42,43^. A few studies have noted increased horn shape diversity in domesticated bovids^42^ and caprids^36,44^, but little is known about this variation in *Capra hircus,* specifically. This important feature of the caprid skull, known for its function in defense and intraspecific competition^42^ has yet to be investigated systemically^11,36^. As a first step in this goal, we provide a figured plate of goat skulls with complete horns that were collected during fieldwork for this study, many directly from Swiss goat breeders, others from museums or the literature, thus documenting variation in a large and diverse tissue of the skull which is also derived from neural crest cells (see above)^45^.

## Materials and methods

### Materials

Seventy-five adult goat crania were analysed: 54 domestic *Capra hircus*, and 21 wild *Capra aegagrus* (bezoar). Both sexes were included, and dental maturity was defined as complete eruption of M3. Effort was made to include a variety of goat breeds, as well as a large sample of bezoar skulls. In total, 17 breeds/populations were sampled from European, African and South American populations, as noted by museum records or breeder accounts (Table 1) (S1). Where breed data were uncertain, descriptions reflect regional provenance, i.e., “Greek_unk” is a Greek goat of unknown breed (see Supplementary S1). Fourteen skulls were collected directly from Swiss breeders affiliated with ProSpecieRara^34^, the Swiss foundation for cultural, historical and genetic diversity of plants and animals. Many of these specimens are presented in Fig. 6A–K. No animals were killed for this study; skulls would have otherwise been discarded by breeders focusing on meat and dairy production.

**Table 1:** Specimens used in analyses. Wild vs domestic analyses: (a) without brachycephalic specimens (main analysis, total n = 70), and (b) with brachycephalic specimens (total n = 75).

	Breed/population	n	Geographical provenance	Breed primary use	Analysis
Wild	n/a	21	Asia Minor	n/a	(a), (b)
Domestic	Capra grigia	5	Europe	Dual	All
	Chamois	4	Europe	Dairy	All
	Criollo	2**	Venezuela	Meat	(a), (b)
	Damaranziege	1**	Africa	n/a	(a), (b)
	Haslitaler	2**	Europe	Dairy	(a), (b)
	Jamtland	1**	Sweden	Dairy	(a), (b)
	Mamberziege*	1	Africa	Unclear	(b), (c)
	Pygmy	7	Europe	Meat and hobby	All
	Saanen	5	Europe	Dairy	All
	Sempione	2**	Switzerland	Meat	(a), (b)
	Stiefelgeissziege	3	Switzerland	Dual	All
	Thebener/Zaraibi*	4	Egypt and Eastern Africa	Unclear	(b), (c)
	Toggenburger	3	Europe	Dairy	All
	Valais Blackneck	6	Europe	Meat	All
	White goat	4	Europe	Dairy	All
	Breed not known	4*	Africa and Greece	n/a	(a), (b)
Total wild + domestic		75			

Breed analysis: (c) only domestic groups with at least three individuals, including all brachycephalic specimens (total n = 42). *n/a* data inapplicable. Breed primary use data were collected from www.prospecierara.ch, Eid et al.^73^, Ekarius^35^ and Kababya et al.^74^.

*Brachycephalic or contains brachycephalics.

**Individual populations too small for breed analysis (c).

#### Wild versus domestic sampling

We conducted two separate analyses comparing wild vs. domestic goats: one without brachychephalic specimens (analysis “a”) and one with them (analysis “b”). In the main analysis (“a”), which tested for craniofacial change correlating with domestication, all brachycephalic^46^ specimens were omitted. Brachycephaly was established according to Geiger et al.^47^. These specimens were members of the Thebener/Zaraibi and Mamberziege breeds: NMW562, MNW2073, NMW2074, K1436, and NMW2072. As in the case of domesticated dogs and other domesticated groups, brachycephaly may be the result of directed artificial selection^13,46, 48^. However, one study suggested that although brachycephaly is common among these populations, they may be primarily bred for meat and dairy consumption^36^. The breeding history of both of these populations is unclear, and their selection is reportedly less formalized than for other goat populations/breeds. Due to the uncertainty in the root of their brachycephaly and the intensity of selection for this condition, the secondary analysis (“b”) was performed with these specimens included, for comparison only.

#### Breed sampling

Breed analyses were conducted on a subset of breeds/populations with at least 3 individuals each, plus all brachycephalics, for a combined dataset of n = 42 specimens from ten different domestic populations (Table 1). Wild goats were omitted from these in order to focus on skull shape differentiation among breeds.

#### Morphometric analyses

**Figure 1:**
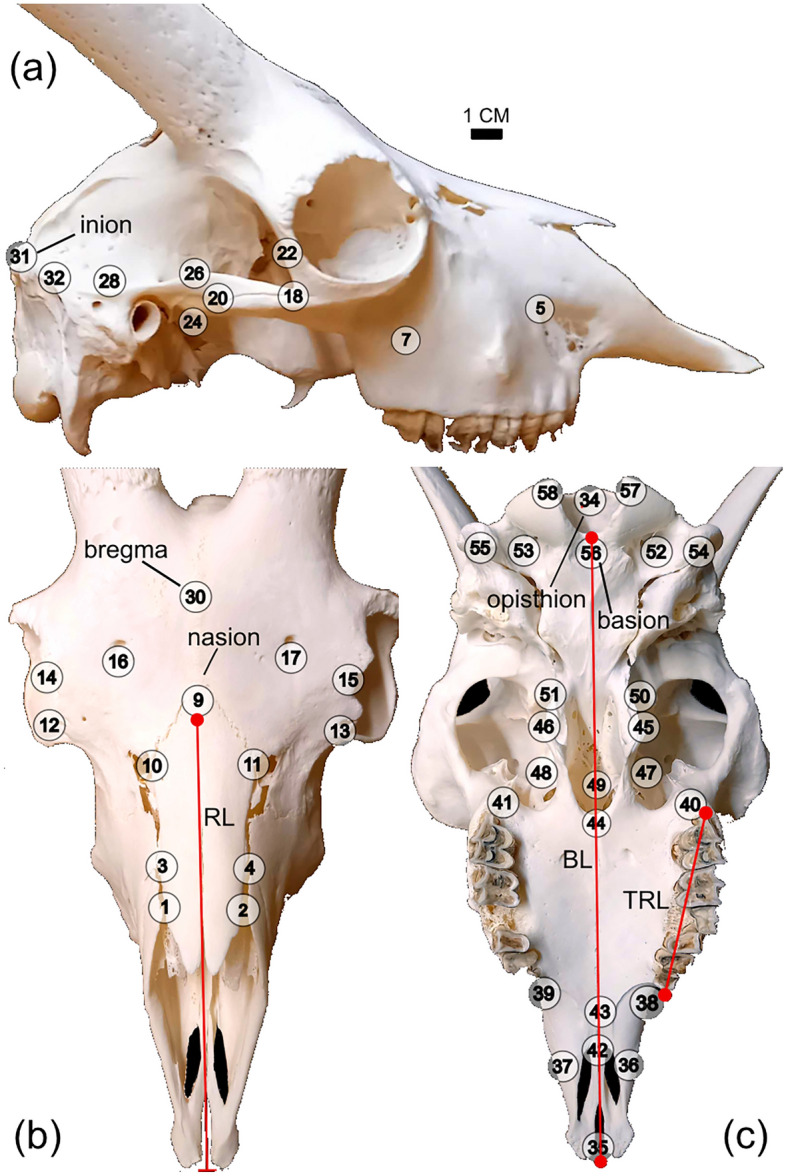
Morphometric landmarks. Landmarks (LMs) (n = 58) used to capture goat skull shape in (**a**) lateral, (**b**) dorsal, and (**c**) ventral views. In red: linear measurements used to test for rostral shortening and tooth row length reduction: *RL* rostral length, *TRL* tooth row length, *BL* basicranial length (body size proxy). Landmark descriptions in Supplementary S2.

Cranial shapes were quantified using 58 bilateral three-dimensional (3D) landmarks (Fig. 1), digitized with a Microscribe (MLX, Revware, Inc., USA) and Microscribe Utility Software (MUS, v.7.0.1.1, Revware, Inc., USA) (SD1). These landmarks have been used in previous studies testing for domestication-related cranial shape change in other taxa^12,16, 47^, and should thus make these collective studies more comparable. Anatomical terminology followed Aiello and Dean^49^. Ventral and dorsal landmarks were collected separately and fused using reference landmarks 34, 36, and 37 (Fig. 1). With the exception of a few wild specimens collected by MG, all morphometric data were collected by the first author. Both authors conferred and cross-checked the landmark placements to ensure consistency in data-collection.

#### Wild versus domestic analyses

Three-dimensional geometric morphometric methods (3DGMM) sensu Bookstein^50^ were employed to determine whether domestic goats exhibit cranial shapes that differ from those of their closest wild sister species. All analyses and visualizations were conducted in R Studio (v.4.0.4)^51^ using the packages geomorph (v.4.0)^52^, Morpho (v.2.9)^53^, mevolCVP (v.5)^54^, MASS (v.7.3-54)^55^, PCDimension (v.1.1.11)^56^, and ggplot2 (v.3.3.5)^57^. Landmark configurations were constructed using generalized Procrustes alignment (GPA)^58,59^. Centroid size (CS) was used as a proxy for both skull and overall body size, and compared between groups using ANOVA after log_10_-transformation. A principal component analysis (PCA)^60^ was used to visualize cranial form variation among wild and domestic goat samples, using the symmetrical component of the Procrustes shape coordinates. The relationship between size (CS) and shape, i.e., allometry, was investigated via multivariate regression of shape coordinates on CS, and tested for statistical significance with a Procrustes ANOVA^50,61, 62^ using an ordinary least squares (OLS) model. Cranial shape disparity, known to increase in many domesticated populations compared to their wild relatives, was estimated using the “morphol.disparity” function of the geomorph package and all Procrustes shape coordinates (see Supplementary S3). To test the statistical significance of mean shape differences between groups we performed a MANOVA, after applying a dimensionality reduction method with the broken stick test from the package PCDimension (Supplementary S3), to avoid overestimating the significance of our results. For comparison, this was also tested with the full set of PCs in a Procrustes ANOVA followed by a Wilcoxon Pairwise test. To test the accuracy of classification of wild versus domestic individuals, we performed a linear discriminant analysis (LDA) using the “mevolCVP” function. This function helps to identify the appropriate number of PCs which maximizes the cross-validated classification rates in the LDA, using leave-one-out cross-validation^54^. The report the “balanced” LDA results, which account for unbalanced samples. The “pldam” function^63^ (predictive LDA, same package) was then used to identify which domestic specimens tended to be misclassified, i.e., were closer to wild goats in shape space^54^. All (M)ANOVAs used a residual randomization permutation (= Monte Carlo) procedure based on 1000 randomized iterations. Probability values were corrected for multi-test comparisons with the Benjamini–Hochberg (BH) procedure^64^ and null hypotheses were rejected at p-values below 0.05. Visualizations of shape changes along PCs were made using 3D thin-plate spline diagrams, or “wireframes”.

Linear distances between landmarks (LMs) were calculated using the Pythagorean Theorem to test for rostral shortening and reduction of tooth row length in the domestic sample compared to the wild condition, i.e., DS traits (Fig. 1). For these tests, brachycephalic specimens were omitted, due to their extreme facial morphology^46^. Rostral and tooth row lengths were normalized for size using basicranial length (BL, Fig. 1) as a body size proxy^15^. Data were log_10_-transformed, compared via Wilcoxon Pairwise tests, and visualized in boxplots.

#### Breed analyses

In order to visualize if cranial shape differences distinguished breeds, a separate PCA was performed on a subset of breeds/populations with three or more representatives, plus all five brachycephalic individuals, for a total sample of n = 42 (see Table 1, Supplementary S4). Due to the small group sizes, no statistical tests were applied.

## Results

### Wild vs domestic goats

**Figure 2:**
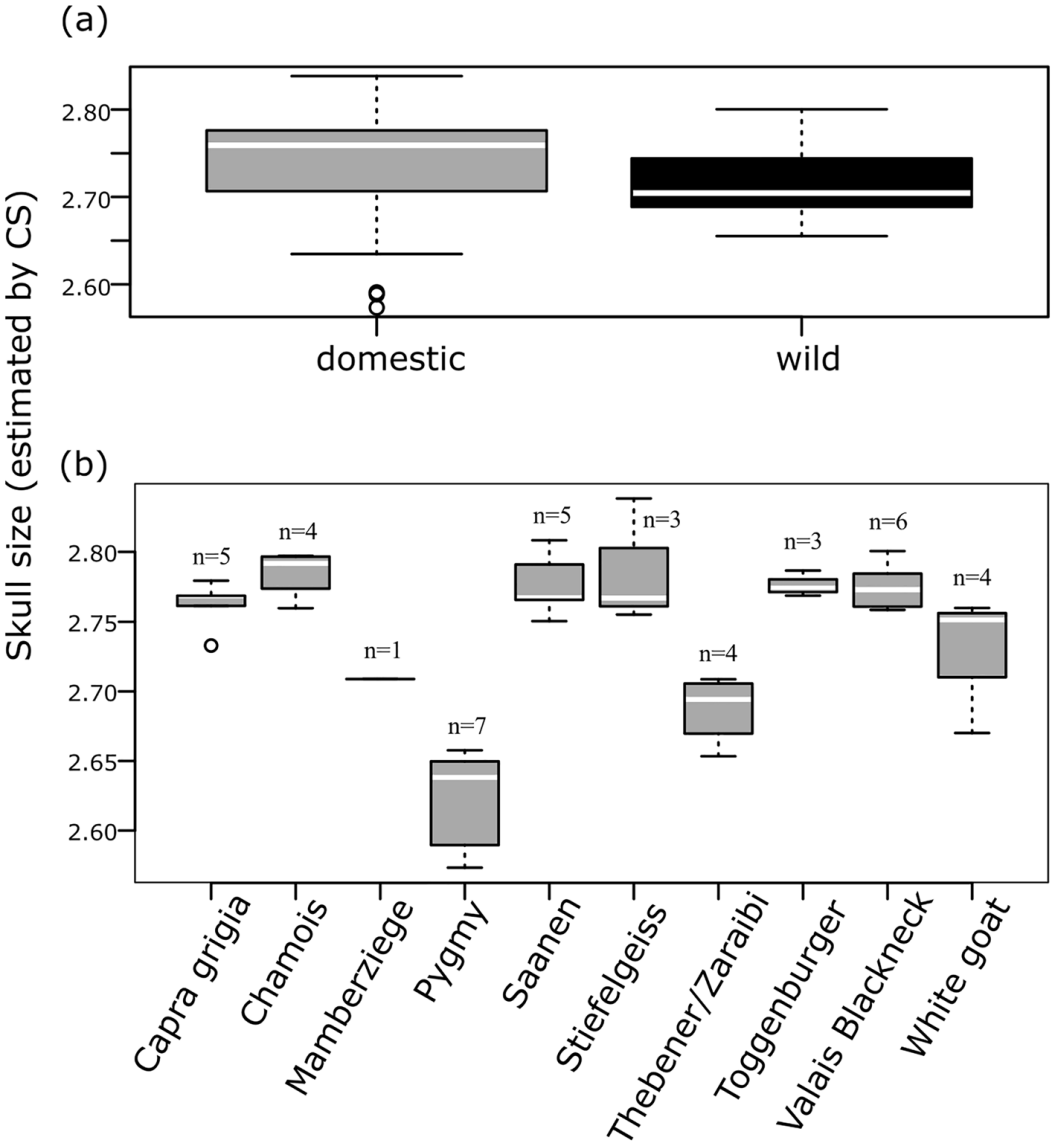
Cranial sizes. Boxplot comparison of cranium sizes, estimated by centroid size (CS). (**a**) Wild (n = 21) vs. domestics (n = 54). (**b**) Only domestic breeds/populations with at least 3 individuals, plus brachycephalic specimens (Mamberziege n = 1, and Thebener/Zaraibi n = 4). Data are log_10-_transformed. Box starts in the first quartile (25%) and ends in the third (75%). Horizontal line = median. Whiskers = extremes of the data range for each group. Open circles = outliers.

Skull size ranges of wild and domestic goats overlap, with Pygmy goats representing outliers (Fig. 2a). Mean skull sizes do not differ (ANOVA: Df_1,73_, F-stat: 1.396, p = 0.241; pairwise Wilcoxon, p = 0.058) (SD3), although there is a trend toward larger skull sizes for domestic breeds in this sample. However, breed cranium size diversity is manifest when the data are structured by breed/population (Fig. 2b), with pygmy goats having the smallest median size and the Chamois and Toggenburger breeds the largest.

**Figure 3:**
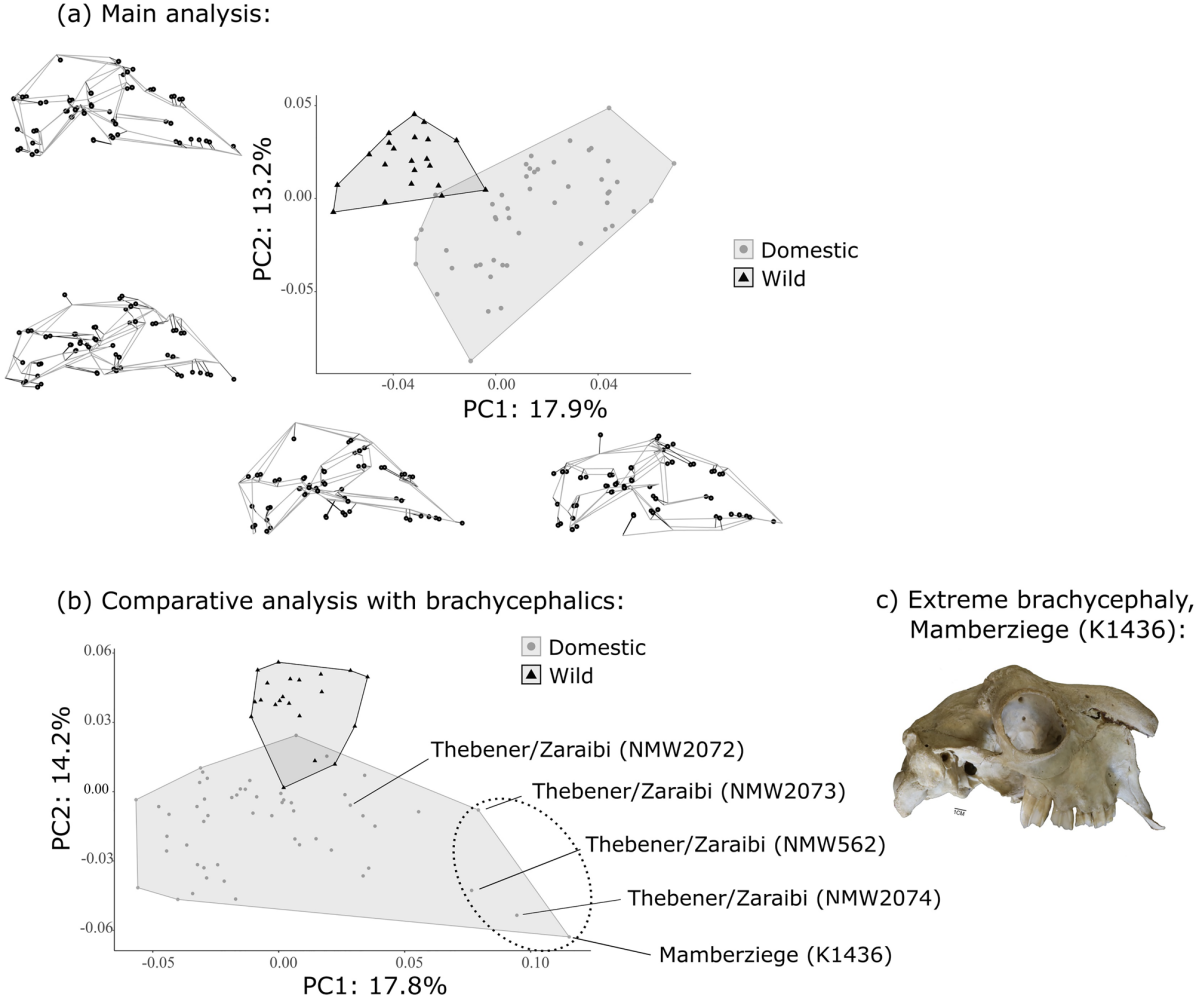
Principal components analyses (PCAs). (**a**) Main wild versus domestic analysis, without the brachycephalic specimens (total n = 70). Wireframes represent cranium shapes in lateral view at extreme ends of each axis; mean shape = black, target shape = gray. Circles = landmarks. Polygons = convex hulls of each sample distribution. (**b**) Comparative analysis with “brachycephalic” Thebener/Zaraibi and Mamberziege populations (total n = 75). Ellipse: observably-brachycephalic specimens. (**c**) Image of specimen at extreme right of PC1, Mamberziege K1436.

A regression of shape coordinates on centroid size revealed that differences in skull size (CS) accounted for approximately 9.5% of skull shape variation (Proc. ANOVA, *F*_1,74_ = 7.62, R^2^ = 0.0945, *p* = 0.001, see Supplementary S3). Since size is an integral aspect of form and one frequently selected under domestication, all PCAs and CVAs were performed without removing size (CS) during GLS Procrustes alignment. The eigenvector-based linear decomposition of the pooled shape covariance matrix resulted in 69 orthogonal components of shape variation, of which the first 30 accounted for 95% of that variation. Principal component 1 and PC2 represent only 17.9% and 13.2% of the pooled form variance, respectively (Fig. 3a). Wild and domestic samples overlap slightly, but mostly occupy distinct areas of the resultant shape space. The greatest distinction between both groups occurs along PC1, which represents variation in the neurocranium including height and shape of the skull roof, and degree of cranial flexion; and variation in facial morphology including dorsoventral positioning of nasals, position/extent of the palate, and position of M3 (Fig. 3a). At least some of the variation observed along PC1 is also due to differences in size (regression of PC1 scores on CS: R^2^ = 0.29, see SD3). Domestics lie mostly at higher values of PC1 relative to wild goats. Accordingly, some domestic goats can display lower/flatter skull roofs, and a more horizontal alignment to their entire skull in lateral view, compared to wild goats. More specifically, the calvarium in domestics appears to reduce in overall size as the nuchal crest (posterior skull, LMs 31–33) shifts anteriorly, and the frontonasal region (LM 9, 16, 17) shifts posteriorly. Domestics can also possess larger nasopalatal cavities, suggested by the lower position of the palate and higher nasals. The palate can also extend further anteriorly but with a striking anterior shift of M3 (LMs 40–41), resulting in relative reduction of the upper tooth row compared to wild goats.

Principal component 2 represents variation in the dorsoventral orientation of the rostrum, i.e., degree of airorhynchy (relatively upward-tilting) or klinorhynchy (relatively downward-tilting), and in the concavity of the face in the nasofrontal region (Fig. 3a). Domestic goats display the full range of shape variation along PC2, while wild goats are restricted to its higher values. Consequently, wild goats have a deeper concavity, or “dent”, at the naso-fronto-lacrimal suture (LMs 10–11) relative to the nasion (LM 9) and anterior nasals (LMs 1–4), and a more downward orientation to the rostrum compared to many domestics. This is indicated by the lower position of the anterior palate (LMs 38–39) and incisive bones in the wild sample (LMs 35–37, 42–43) (see Figs. 1, 3a).

The broken stick test found the first six PCs to be statistically significant and carry a cumulative 62% of the total variance structure (see Supplementary S3). The mean shapes of wild and domestic samples are significantly different with respect to their respective sample variances, based on both full-dimension and reduced-dimension variance tests: MANOVA, *F*_1,6_ = 33.1, *p* < 0.0001; ANOVA, *F*_1,68_ = 9.67, *p* = 0.001). There are no statistical differences in disparity between the two samples (*p* = 0.5, see Supplementary S3).

When brachycephalic specimens were included in a secondary PCA (Fig. 3b), the resultant form space had a markedly enlarged PC1 axis with a maximum value above 0.10, compared to the main analysis (Fig. 3a). Space occupation and orientation relative to the ordination axes changed for both wild and domestic samples. Except for one individual (NMW2072), all Thebener/Zaraibi and Mamberziege individuals projected to positions at the highest extremes of PC1 (Fig. 3b, in red ellipse), arguably as outliers to all other domestics, supporting their exclusion from the main analysis (Fig. 3a). The extreme craniofacial morphology at the right limit of PC1 is pictured in Fig. 3c.

The balanced LDA correctly classified wild versus domestic individuals with a mean accuracy of 96.5%. The 90% interval ranging from 92.8 to 100.0%. The maximum classification accuracy was achieved with the first seven PCs which together comprised 65.7% of the total shape variance structure. Domestic specimens with a slightly closer affinity to wild goats (classification rates: 75–81%) were one Greek goat of unknown breed (NMW2069) and one Pygmy (K18059). Misclassified wild specimens (ZIN439, ZIN1052, ZIN31215; classification rates: 60–71%) had no notable differences in metadata—all were from Asia Minor and from the same museum collection (Supplementary S3).

### Specific trends in domestication

**Figure 4:**
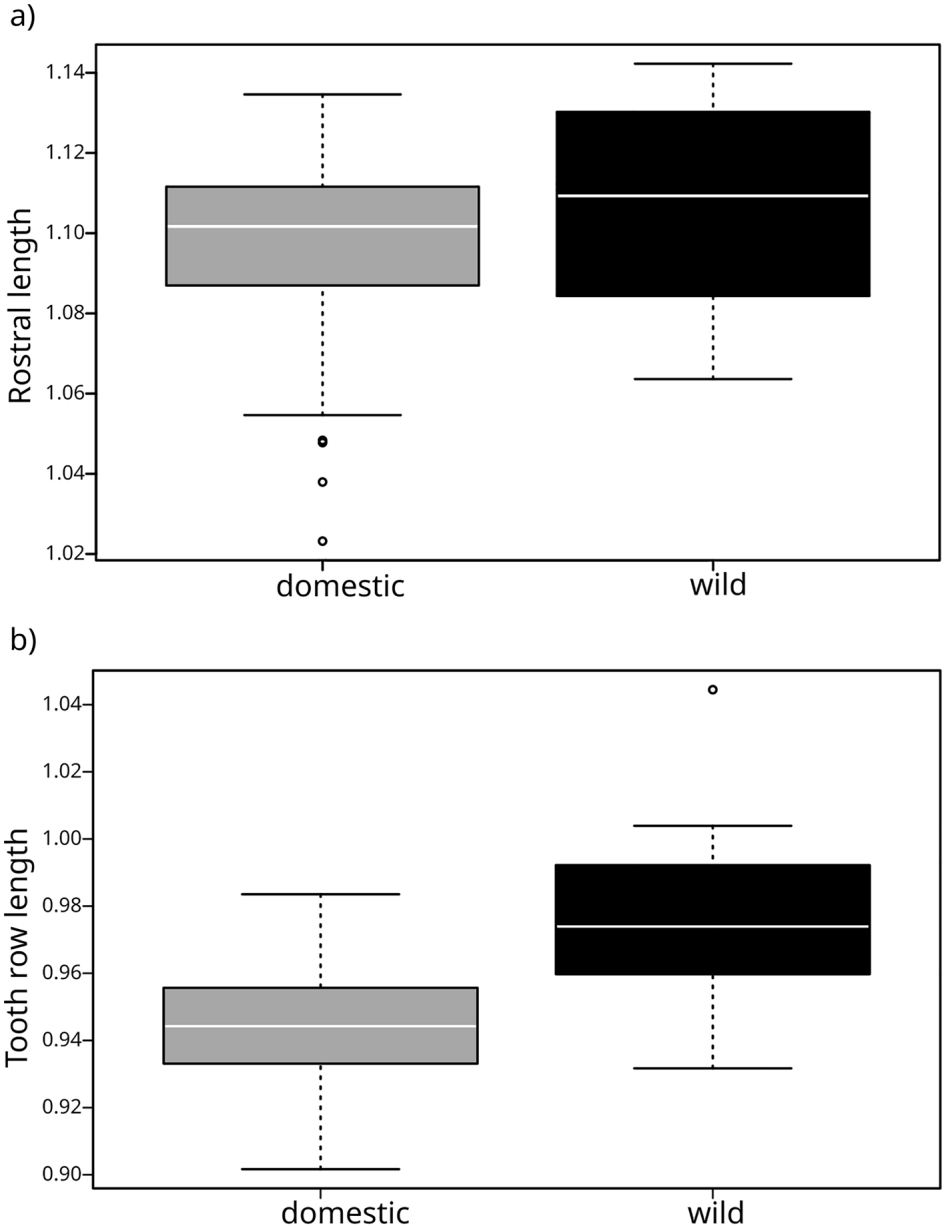
Domestication trends. Testing trends claimed by the Domestication Syndrome (DS)^19^. Comparison of (**a**) rostral length, and (**b**) tooth row length between wild (n = 21) and domestic (n = 54) groups. Dimensions are normalized for size using basicranial length (BL) as body size proxy (Fig. 1). Data are log_10-_transformed. Box starts in the first quartile (25%) and ends in the third (75%). Horizontal line = median. Whiskers = extremes of the data range for each group. Open circles = outliers.

There is a trend towards shortening of the rostrum in domestics (n = 54) compared to wild goats (n = 21), but not a statistically significant one, based on a p-value cutoff of 0.05 (Wilcoxon test p-value = 0.073) (Fig. 4a). Relative tooth row length is significantly shorter in the domestic sample (Wilcoxon test p-value < 0.0001) (Fig. 4b).

#### Breed analyses: potential trends in shape variation

**Figure 5:**
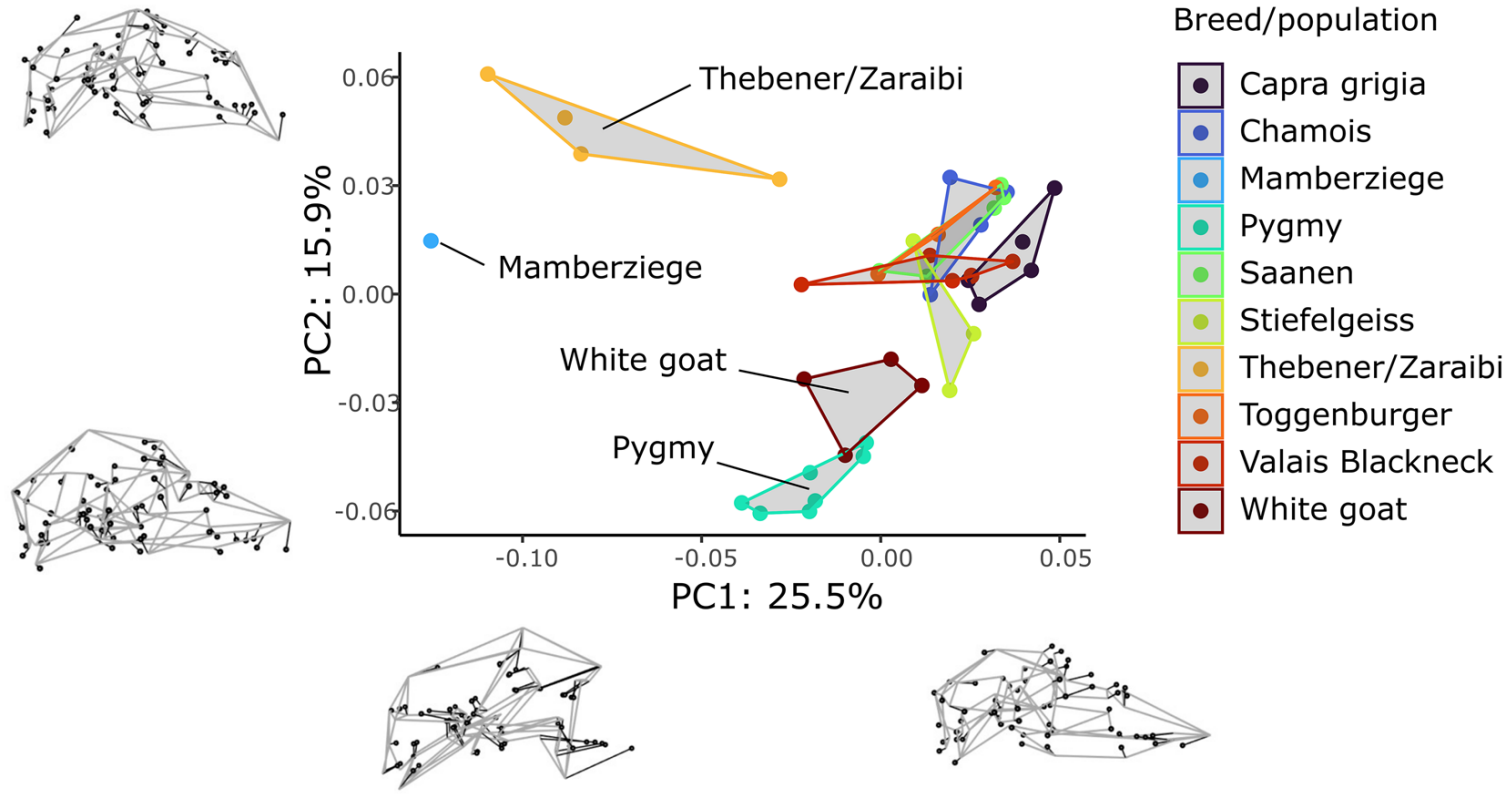
Breed analysis. Principal components analysis (PCA). Wireframes represent cranium shapes in lateral view at extreme ends of each axis; mean shape = black, target shape = gray.

The breed PCA (Fig. 5) differs from the wild versus domestic PCA in using a pruned dataset of breeds/populations with at least 3 representatives plus all five brachycephalics, and no wild sample (see Table 1, Supplementary S4). This visualization serves simply to expose potential trends in shape variation across breeds that could be tested with larger datasets. The resultant form variation space was composed of 41 PCs of which the first 21 captured ~ 95% of the total variation in the pooled sample. PC1 represents 25.5% of the total variance, and is associated changes in the convexity of the frontonasal region and degree of klinorhynchy (Fig. 5). All bracycephalic specimens (Mamberziege and Thebenener/Zaraibi) were, unsurprisingly, separated from the remaining eight populations, at low values of PC1 where the frontonasal region is enlarged and convex, and the rostrum has an extremely downward-pointing orientation. We observe some separation between both brachycephalic populations: Mamberziege and Thebener/Zaraibi. Principal component 2 carries 15.9% of the variance structure and is characterized by variation in the dorsoventral orientation of the rostrum, as well as differences in the anteroposterior positions of nasals and frontals (Fig. 5). A possible trend here may be the slight separation of Pygmy and White goats in the low values of PC2 from the cluster of remaining breeds. Due to the significantly smaller body size of Pygmy goats (Fig. 2, Supplementary S4), a secondary breed PCA was performed with size-adjusted shape scores (Supplementary S4) which caused Pygmy goats to project to positions in closer proximity to other breeds.

## Discussion

Our sample included 17 populations from various environments, body sizes, and geographic regions (Table 1). Although it is not representative of the global diversity of domesticated goats, it provides a robust preliminary dataset to explore the impact of domestication on cranial shape in goats. We find that wild and domestic groups differ significantly in their mean cranial shapes, and can be correctly distinguished by linear discriminant analysis with great accuracy (96.5%). However, the two groups do not differ in their respective amounts of morphological disparity. Size is partly responsible for differences along the main axis of variation (PC1). However, domesticated goats can display neurocranial form variation including reduced cranial flexion, flatter skull roofs, and more horizontal anteroposterior alignment of the skull—variation that has also been observed in domesticated llamas and alpacas^12^. We note one morphological variation that has not been reported in other domestics: an apparent reduction in the size of the calvarium, marked by simultaneous constriction of the nuchal crest and frontonasals towards the bregma. This may be visible, in part, to the high landmark density of our analysis. Changes in neurocranial form are of particular relevance in domestication studies since relative brain size reduction is one of the most consistent trends observed among domesticated taxa^10,11, 25, 40, 65^. An endocranial volume study reported reduced brain size of approximately 15.5% for domestic goats compared to their closest wild relatives^40^.

Domesticated goats also display notable variation in the rostrum. Compared to wild goats, domestics can display an enlargement of the nasopalatal space combined with a lower palate, and more dorsal orientation of the palate and nasals. A more dorsal orientation of the rostrum—i.e., increased airorhynchy—has also been reported for domesticated pigs^14^ and South American camelids^12^, but not an expansion of the nasal cavity. We suggest a larger nasal space may be related to enhanced respiratory function in domesticated goats, possibly taken to the extreme in brachycephalic populations (see Geiger et al.^47^ for further discussion). Alternatively, the lowering of the palate could also be associated with changes in diet and feeding strategy. Several features of the mouth, palate, and tooth row have been tested for correlation with different feeding efficiencies in grazers and browsers^66^, but not dorsoventral positioning of the palate.

In contrast to other domesticated species (e.g., dogs: Drake and Klingenberg^13^; pigs: Owen et al.^14^; cattle: Veitschegger et al.^41^), skull form variation differences between wild and domesticated goats appear to result from low-magnitude phenotypic changes spread over many aspects of the form. The principal axes of variation (PCs 1–2) reflect low percentages of form/shape variation. One concern when exploring the principal axes, as is common in these types of studies, is that lower-magnitude phenotypic changes may go unnoticed. Principal component eigenvalues are a function of comparisons between patterns of variation across landmark pairs and depend on the number of landmarks and sample sizes analyzed. Despite variation in study parameters, however, other domesticated groups including pigs, dogs, and horses^13,14, 16^ display much higher magnitude changes along the principal directions of shape change (PCs). In this respect, the skull shape variation structure of wild and domesticated goats is most similar to that of wild and domesticated South American camelids^12^—a group that has also experienced relatively fewer directions of artificial selection compared to other domesticated groups including pigs^14^ and dogs^13^. Most domesticated goat populations are considered a means of subsistence for small farmers^31^, and their management, at a global scale, is considered much less organized compared to that of other domesticated groups^67^. In fact, their global success is primarily due to their hardiness and ability to survive in a variety of harsh environments^31^ rather than to organized breeding or human management^67^. Consequently, we suggest that the great variety of low-magnitude cranial shape changes observed in domesticated goats compared to wild goats—a pattern that differs from those observed in comparable studies on other domesticated groups—may result from the relatively lower intensity of their selection and husbandry. This is a topic that requires much further investigation, but is a pattern observed in domestic camelids^12^ and now also in domestic goats. Furthermore, we confirm that cranial shape changes occur in correlation with domestication even under lower-intensity selection regimes as noted above.

Despite differences in cranial form, disparity did not increase significantly in domestic goats compared to the wild sample. This is likely due to our sampling which includes 17 out of approximately 300 goat breeds that exist globally^31^. We propose two other possible explanations. First, is the low level of global genetic diversity reported for domestic goats compared to other ungulates^31^: over 90 percent of domestic goat populations worldwide carry the same mtDNA haplotype A, and is likely related to their historically extensive international transport^68^. Second, is the aforementioned simpler and less variable selection applied in the husbandry of domestic goats compared to other livestock^31^. Only a small proportion of global goat populations (mostly European breeds) are under well-established breeding regimes^31,67^. In fact, although goat milk is available in much of the world, less than 5% of the milk produced is marketed, and the goat meat industry is considered ‘not well-organized’^67^. Weaker husbandry practices have also been discussed as a possible reason for reduced variability in gestation length for other domesticated artiodactyls^69^. A broader sampling of global goat breeds may yield different results in terms of disparity.

We found only a slight trend towards a shortening of the snout in domesticates, which was not statistically significant based on a *p*-value cutoff of 0.05. However, a *p*-value of 0.073 leaves much room for interpretation and future testing. Rostral shortening continues to be a debatable trend in domesticated groups and in discussions of the DS hypothesis^4,5, 19^. It does not appear to occur in domesticated South America camelids^12^, and is reportedly absent in domesticated rabbits. However, the clear and significant shortening of the tooth row in domestic mammals supports a trend previously reported for some domestic species and is in line with the DS hypothesis^19^.

It is possible that cranial shapes differ between certain breeds, but this is something to be tested with a larger dataset. Preliminarily, not only are brachycephalics different from all other breeds, but they appear to vary among themselves, with the Mamberziege falling far from the Thebener/Zaraibi breed in morphospace (Fig. 5). Additionally, Pygmy goats along with White goats appear more distant from the remaining domestic sample (Fig. 5). In a supplementary analysis where size (CS) was removed from the breed PCA, Pygmy goats, and to a lesser extent, White goats, maintained a degree of shape distinction, suggesting that extreme body size selection in Pygmies has not resulted in a complete loss of shared allometric components with other breeds (SD4). In other words, some cranial form differences in Pygmies are related to body size selection, but certainly not all. We note here that PCA alone might not identify between-group differences with great accuracy^70^, but rather discriminant analyses or a combination of both is recommended for purposes of distinguishing groups^71^. We suggest our interpretations be tested with a dataset including many more breeds like the Pakistani dancing goats, myotonic goats, wool-producing Angoras, or rare breeds like the Arapawa and Kiko of New Zealand^35^.

Unlike what has been observed in cattle^41^, cranial form does not appear to be associated with main breeding purpose in goats. Brachycephalic breeds are likely multi-purpose goats, and the reason for their brachycephalic condition is unclear^36^; White goats are bred exclusively for dairy production, while Pygmy goats are known to be bred for both meat and hobby^35^.

**Figure 6:**
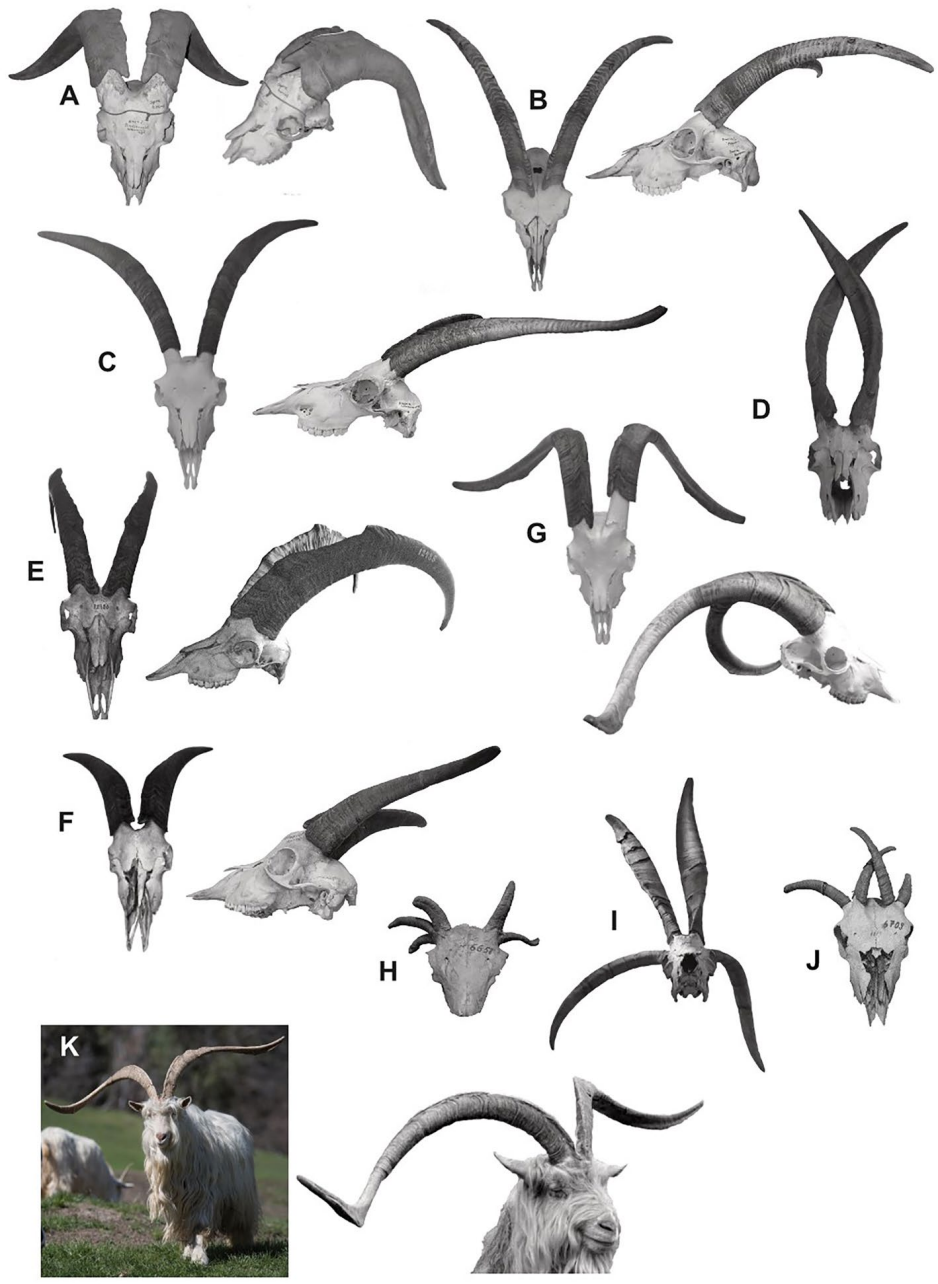
Horn plate. Domestic goat horns (**A–D,F–K**) are remarkably diverse compared to the wild form (**E**), although horn form may be shared across different breeds (e.g., Sempione and Valais Blackneck: **B,C**), or may differ between the sexes of one breed (e.g., Sempione female and male: **B,J**). Skulls are shown in frontal view and lateral view (when available). Images are scaled approximately to similar length, i.e., they are not to scale. Note that the selection of breeds/populations shown here is not comprehensive. (**A**) Unknown breed (ZMB Mamm A14011); (**B**) Sempione, female (Naters1_Mayara); (**C**) Valais Blackneck (Naters_1666203); (**D**) unknown breed (ZMUZH 17734); (**E**) wild goat, *Capra aegagrus* (ZIN12488); (**F**) Capra grigia (Spirito_Florian1); (**G**) Pfauenziege/Taubenziege (Althus1); (**H**) unknown breed (AF0630); (**I**) unknown breed (ZMB Mamm #6658); (**J**) unknown breed (ZMB Mamm #6709); (**K**) Sempione male (“Albino” in his natural environment). Specimen details in S5.

Documentation of horn shape variation in goats (*Capra hircus*) has been mostly anecdotal. The straight, scimitar-shaped horns of the bezoar are reportedly similar to those of Neolithic-aged domestic goats^72^. Later in the Bronze age, domestic goats had increasingly more twisted horns or were even polled^31^. Horn cores became more angular in cross-section, more bilaterally-compressed, or even triangle-shaped, diverging from the round horn cores of the bezoar^31,72^. Our survey (Fig. 6A–K) confirms and highlights the greater horn shape diversity in domesticated goats compared to wild goats. But, as in other cranium form/shape studies related to domestication, this survey has not exhausted all the potential empirical results or interpretations of those results. It is an easy matter to imagine horn forms and shape that have never been recorded for domestic goats^11^.

## Conclusions

Finding universal patterns of morphological variation between wild and domestic populations continues to be challenging. In this study we report on the modes of cranial form variation between wild and domestic goats, and find that their morphological differences result in highly accurate distinction of the two groups (96.5%) in linear discriminant analyses. Domesticated goats display a greater variety of cranial form changes in neurocranial and facial regions than reported for other domesticated Artiodactyls, although these changes are lower in magnitude and are spread across many aspects of cranial form, similar to the case of llamas and alpacas, which have been similarly studied^12^. The low magnitudes may be associated with fewer directions of artificial selection in goat husbandry (as is the case with llamas and alpacas) compared to other livestock, as well as with the low genetic diversity across global goat populations. These hypotheses require much further testing. A notable finding is the apparent reduced size of the domestic goat calvarium, which may be related to the ~ 15.5% endocranial volume reduction of domestic goats compared to their closest wild relatives^40^. Variation in the location and orientation of palate and nasals appears to result in an enlarged nasopalatal cavity, possibly related to respiratory or masticatory adaptations, but research in this area is lacking. Other cranial form variations observed here align with observations in other domestic taxa, such as a straighter anteroposterior alignment of the skull, a flatter skull roof, and variation in degree of airprhynchy/klinorhynchy. Among breeds, only further testing may confirm if cranial shape differences exist between different brachychephalic populations. We find partial support for the DS hypothesis with a reduction in tooth row length for the domestic sample. The test for rostral shortening is not definitive in this sample, although a slight trend towards shortening is observed. In summary, wild and domestic goat cranial shapes differ significantly, and expand on the cranial form differences associated with domestication. The observed variation does not correlate with main breed use, as it does in other domesticated taxa.

### Supplementary Information

Supplementary Information.

## Data Availability

All data, analyses, and r-scripts for this study are provided in Supplementary Files S1–S9.
