# Supplementary material for: Cranial form differences in goats by breed and domestic status
Source: Sci Rep. 2024 Jan 9;14:917. doi: 10.1038/s41598-023-50357-0 (PMC10776561; doi:10.1038/s41598-023-50357-0)
Supplement: Supplementary file 1 — Supplementary Information. [file 41598_2023_50357_MOESM1_ESM.pdf]

Cranial form differences in goats by breed and domestic status

A.M. Balcarcel[1], M. Geiger, M.R. Sánchez-Villagra*

Appendix S1_Supplementary table 1. Samples and data

Institutional abbreviations: ZMB_Mamm: Nehring Collection, Zoologische Sammlung der Königlichen Landwirtschaftlichen Hochschule zu Berlin, Museum für Naturkunde Berlin; NHMB: Naturhistorisches Museum Bern; PIMUZ: Paleontology Institute and Museum, UZH, Zurich; I.f.H: Zoologisches Institut/Populationsgenetik (former Institut für Haustierkunde), Christian-Albrechts-Universität zu Kiel, Germany; V_UB: Vetsuisse Faculty, University of Bern; NMW: Naturhistorisches Museum Vienna; ZIN: Zoological Museum of the Zoological Institute of the Russian Academy of Sciences.

ID	Collection	Sex	*Genus*	*species*	W_D	breed_variety	region
BZM68636	ZMB_Mamm	na	*Capra*	*aegagrus*	wild	wild	Asia_Minor
BZM68520	ZMB_Mamm	na	*Capra*	*aegagrus*	wild	wild	Asia_Minor
BZM68616	ZMB_Mamm	na	*Capra*	*aegagrus*	wild	wild	Asia_Minor
BZM8999	ZMB_Mamm	na	*Capra*	*aegagrus*	wild	wild	Asia_Minor
BZM68641	ZMB_Mamm	na	*Capra*	*aegagrus*	wild	wild	Asia_Minor
ZIN3820	ZIN	f	*Capra*	*aegagrus*	wild	wild	Asia_Minor
ZIN439	ZIN	f	*Capra*	*aegagrus*	wild	wild	Asia_Minor
ZIN3806	ZIN	f	*Capra*	*aegagrus*	wild	wild	Asia_Minor
ZIN3804	ZIN	na	*Capra*	*aegagrus*	wild	wild	Asia_Minor
ZIN1052	ZIN	m	*Capra*	*aegagrus*	wild	wild	Asia_Minor
ZIN12479	ZIN	f	*Capra*	*aegagrus*	wild	wild	Asia_Minor
ZIN12488	ZIN	m	*Capra*	*aegagrus*	wild	wild	Asia_Minor
ZIN447	ZIN	f	*Capra*	*aegagrus*	wild	wild	Asia_Minor
ZIN12490	ZIN	m	*Capra*	*aegagrus*	wild	wild	Asia_Minor
ZIN6956	ZIN	f	*Capra*	*aegagrus*	wild	wild	Asia_Minor
ZIN19042	ZIN	f	*Capra*	*aegagrus*	wild	wild	Asia_Minor
ZIN19043	ZIN	f	*Capra*	*aegagrus*	wild	wild	Asia_Minor
ZIN19041	ZIN	f	*Capra*	*aegagrus*	wild	wild	Asia_Minor
ZIN958	ZIN	m	*Capra*	*aegagrus*	wild	wild	Asia_Minor
ZIN18249	ZIN	m	*Capra*	*aegagrus*	wild	wild	Asia_Minor
ZIN31215	ZIN	m	*Capra*	*aegagrus*	wild	wild	Asia_Minor
NMW2072	NMW	m	*Capra*	*hircus*	domestic	Thebener/Zaraibi	Africa
NMW2073	NMW	f	*Capra*	*hircus*	domestic	Thebener/Zaraibi	Africa
NMW562	NMW	m	*Capra*	*hircus*	domestic	Thebener/Zaraibi	Africa
NMW2074	NMW	m	*Capra*	*hircus*	domestic	Thebener/Zaraibi	Africa
K1436	IfH	na	*Capra*	*hircus*	domestic	Mamberziege	Africa
Federer1	NHMB	m	*Capra*	*hircus*	domestic	Capra_grigia	Europe
103843	NHMB	m	*Capra*	*hircus*	domestic	Capra_grigia	Europe
Florian_1	NHMB	na	*Capra*	*hircus*	domestic	Capra_grigia	Europe
Florian_2	NHMB	na	*Capra*	*hircus*	domestic	Capra_grigia	Europe
Florian_3	NHMB	na	*Capra*	*hircus*	domestic	Capra_grigia	Europe
PIM10892	NHMB	na	*Capra*	*hircus*	domestic	Chamois	Europe
PIM10882	NHMB	na	*Capra*	*hircus*	domestic	Chamois	Europe
PIM10880	NHMB	na	*Capra*	*hircus*	domestic	Chamois	Europe
PIM10888	NHMB	na	*Capra*	*hircus*	domestic	Chamois	Europe
Venezuela_1237	PIMUZ	na	*Capra*	*hircus*	domestic	Criollo	South_America
Venezuela2_1236	PIMUZ	na	*Capra*	*hircus*	domestic	Criollo	South_America
K19506	IfH	f	*Capra*	*hircus*	domestic	Damaranziege	Africa
K7558	IfH	m	*Capra*	*hircus*	domestic	African_unk	Africa
NMW18639	NMW	m	*Capra*	*hircus*	domestic	Greek_unk	Europe
NMW2069	NMW	f	*Capra*	*hircus*	domestic	Greek_unk	Europe
NMW2559	NMW	f	*Capra*	*hircus*	domestic	domestic	na
Bern314	V_UB	f	*Capra*	*hircus*	domestic	Haslitaler	Europe
Bern301	V_UB	m	*Capra*	*hircus*	domestic	Haslitaler	Europe
K1556	IfH	f	*Capra*	*hircus*	domestic	Jamtland	Europe
K20591	IfH	m	*Capra*	*hircus*	domestic	Pygmy	Europe
K21179	IfH	f	*Capra*	*hircus*	domestic	Pygmy	Europe
K19238	IfH	m	*Capra*	*hircus*	domestic	Pygmy	Europe
K19235	IfH	na	*Capra*	*hircus*	domestic	Pygmy	Europe
K22336	IfH	f	*Capra*	*hircus*	domestic	Pygmy	Europe
K18059	IfH	f	*Capra*	*hircus*	domestic	Pygmy	Europe
Bern316	V_UB	na	*Capra*	*hircus*	domestic	Pygmy	Europe
PIM10883	PIMUZ	na	*Capra*	*hircus*	domestic	Saanen	Europe

PIM10895	PIMUZ	na	*Capra*	*hircus*	domestic	Saanen	Europe
PIM10881	PIMUZ	na	*Capra*	*hircus*	domestic	Saanen	Europe
PIM10891	PIMUZ	na	*Capra*	*hircus*	domestic	Saanen	Europe
PIM10884	PIMUZ	na	*Capra*	*hircus*	domestic	Saanen	Europe
103848	NHMB	f	*Capra*	*hircus*	domestic	Sempione	Europe
103857	NHMB	na	*Capra*	*hircus*	domestic	Sempione	Europe
103854	NHMB	f	*Capra*	*hircus*	domestic	Stiefelgeiss	Europe
103852	NHMB	f	*Capra*	*hircus*	domestic	Stiefelgeiss	Europe
103851	NHMB	m	*Capra*	*hircus*	domestic	Stiefelgeiss	Europe
PIM10893	PIMUZ	na	*Capra*	*hircus*	domestic	Toggenburger	Europe
PIM10903	PIMUZ	na	*Capra*	*hircus*	domestic	Toggenburger	Europe
PIM10879	PIMUZ	na	*Capra*	*hircus*	domestic	Toggenburger	Europe
K1438	IfH	f	*Capra*	*hircus*	domestic	Valais_Blackneck	Europe
K30447	IfH	f	*Capra*	*hircus*	domestic	Valais_Blackneck	Europe
103850	NHMB	f	*Capra*	*hircus*	domestic	Valais_Blackneck	Europe
103856	NHMB	na	*Capra*	*hircus*	domestic	Valais_Blackneck	Europe
103861	NHMB	na	*Capra*	*hircus*	domestic	Valais_Blackneck	Europe
103860	NHMB	na	*Capra*	*hircus*	domestic	Valais_Blackneck	Europe
K15442	IfH	f	*Capra*	*hircus*	domestic	White_goat	Europe
K18719	IfH	na	*Capra*	*hircus*	domestic	White_goat	Europe
K28513	IfH	f	*Capra*	*hircus*	domestic	White_goat	Europe
K28514	IfH	f	*Capra*	*hircus*	domestic	White_goat	Europe

x1	y1	z1	x2	y2	z2	x3	y3	z3
-6.188	24.0918	31.7274	12.9209	25.3264	32.3501	-7.193	32.9659	34.6927
2.0563	32.6666	35.1881	19.0564	32.334	31.9392	0.9809	39.2147	37.2192
5.7671	24.3557	33.5311	21.6362	22.1739	31.2546	6.2851	31.58	34.7692
-3.9393	34.1794	38.7297	16.3304	37.4266	36.895	-4.0085	43.1136	40.1239
3.0576	25.8792	36.763	24.2706	26.0478	34.9535	4.9676	34.9629	39.7953
5.9746	28.9434	35.7841	21.0478	31.7231	36.7103	-1.1786	31.9635	32.9233
-4.8455	25.1903	40.2364	26.6574	27.4798	41.0286	-8.9861	28.3945	37.1111
-8.6528	23.5675	36.2292	8.3454	26.6908	33.8586	-12.9805	25.5197	32.009
-7.2825	42.5913	48.6403	17.3358	38.0452	48.8575	-9.702	49.2701	47.0644
-2.4696	27.1974	48.9614	24.4719	23.7959	50.4857	-3.4071	38.2905	51.5071
-4.3044	33.4606	41.9626	14.096	31.1246	40.3935	-4.9514	43.4408	42.7599
-9.9034	30.1942	44.5261	15.4347	34.8833	46.5871	-11.7404	42.0252	49.5161
1.3725	29.9294	36.483	21.4639	30.0458	36.8407	0.716	35.3445	37.5584
-0.5829	36.79	43.5792	21.5463	35.7802	43.8023	-1.1816	48.5867	46.542
-3.9519	29.1876	34.7652	12.1516	28.641	34.6904	-6.1614	35.4039	35.8018
-0.4559	32.5997	41.3348	14.7575	31.6393	40.6681	-1.2642	44.5332	45.7827
-3.4659	34.798	38.298	17.0425	30.9235	36.1621	-4.6699	43.9125	41.1555
-3.3866	32.7412	38.2394	16.73	31.6588	37.588	-3.3122	42.2731	40.3891
-6.0444	32.1957	43.2254	22.3695	37.5569	44.6903	-7.5972	40.87	45.4324
3.6279	39.0139	48.468	26.8352	34.1684	45.4406	5.6489	51.3575	52.1721
-2.0131	42.0533	51.6067	22.3557	41.2647	50.0239	-3.5386	54.3942	55.5821
-3.2188	25.2953	42.626	18.8301	22.6784	41.8898	-4.092	29.3615	44.4951
-4.337	12.3328	41.4929	15.5251	13.1802	45.6534	-4.7868	13.8874	42.5719
-0.4195	14.9451	52.854	22.8124	14.1683	54.0161	0.2874	18.1228	55.2195
2.6794	3.8485	47.5135	25.8485	5.4692	48.2924	0.9123	6.5259	47.6769
-6.2657	12.4418	54.2324	18.5782	9.8433	53.3152	-6.7782	17.2675	57.6105
-5.1789	32.5122	39.2151	16.9917	33.6906	39.7276	-6.7192	40.3969	40.9685
-1.9311	34.7164	52.9075	21.3465	32.6664	48.2892	-3.0103	47.0549	54.5563
-5.5627	35.8066	54.1343	17.6223	35.9402	49.254	-7.2849	47.7622	55.009
-4.3031	30.5286	44.1345	17.2799	33.9588	46.0907	-5.5785	39.9297	46.3369
4.5039	36.7278	46.4029	26.5663	34.0789	45.697	2.9204	43.594	46.9571
0.5774	31.9917	41.668	22.5665	27.5918	41.603	1.7289	40.0709	43.2062
-7.0977	34.7873	47.7183	19.1355	35.6985	50.4992	-9.0871	48.7596	52.1283
-8.2471	32.084	49.1705	11.7579	35.9067	54.0394	-8.3672	46.6669	56.2317
-0.1367	38.471	55.4439	26.3214	37.307	54.8704	-1.1175	51.7124	58.7857
1.9435	26.0355	44.3468	22.7943	25.9517	45.1244	1.4505	35.2588	46.8188
2.5433	29.7322	37.9433	21.3327	31.0916	38.347	1.5737	37.4101	40.4402
-1.9945	31.4033	39.8317	26.1068	33.2969	43.1547	-2.3523	39.8756	42.2296
5.7432	17.6788	30.8868	21.9823	14.6028	31.8794	7.2443	25.3159	34.5241
-0.078	31.8073	44.6586	21.5274	26.9805	43.4029	-1.4675	37.7835	46.9487
-6.6886	26.3645	37.7457	19.3613	30.6705	35.2249	-5.8735	28.3881	37.3534
-8.1276	19.9673	46.0826	24.8395	26.4008	47.1755	-9.5002	26.5034	49.6969
-0.0544	-0.5157	-0.7017	27.8684	0.5036	0.009	-0.1617	4.5657	3.6514
0.1316	-0.5638	0.1664	27.6644	0.7668	1.7925	-1.2323	5.8485	4.7158
0.3676	13.6458	48.7254	22.0621	14.4316	48.646	-1.8199	31.7577	54.4342
-1.826	24.8634	34.1613	18.3055	26.2868	35.5883	-2.5265	30.2341	35.5951
-0.7719	22.9821	31.1218	18.5788	26.2184	33.3023	-1.6936	29.1565	33.1439
-0.5204	22.965	30.9404	18.069	23.1311	31.8876	-2.3994	38.3486	36.6685
-0.5895	19.385	28.5374	14.866	17.7021	28.5973	-0.5124	27.186	31.3917
-3.2289	28.2254	35.613	17.4473	30.2644	35.9347	-4.4584	32.1884	35.8703
-0.5073	20.5244	29.4783	14.59	22.1264	29.5302	-3.1686	26.5945	30.1824
0.7433	-0.6785	0.243	17.3241	-0.0285	0.1285	-0.4089	2.6814	2.2288
-7.7067	36.1193	43.6604	14.2942	37.8516	46.9327	-8.4672	44.8076	46.8611

-4.3437	34.7284	48.183	16.7624	36.8768	45.2892	-5.0416	44.0755	48.5838
-7.8367	38.1924	45.1444	16.2726	45.529	46.3013	-8.7083	49.2786	50.415
-10.5324	31.2816	46.7427	14.0739	35.1495	42.3194	-10.9793	36.9596	48.2043
-1.9251	40.0064	50.9168	24.5816	38.8102	49.7256	-1.1506	54.1265	55.599
0.2088	-0.1574	-0.6293	19.6346	-0.0008	0.5165	-3.0261	10.2549	4.8265
13.7531	42.4184	45.0814	34.1054	33.9354	42.9521	16.1413	46.583	45.9623
8.5687	35.8067	43.2773	31.591	29.5568	45.7372	8.6867	48.0287	48.2273
-11.8874	34.8387	42.9499	15.199	39.9165	43.0709	-12.2838	48.2628	46.6455
9.2855	30.8265	56.521	34.7086	25.2217	56.4121	12.658	45.7807	61.0178
3.8899	31.0483	49.7564	27.9098	26.2496	47.2939	6.6227	39.1933	50.8316
-0.9162	33.4389	49.6436	21.4118	34.4303	50.9664	-1.143	38.5507	51.2237
-3.7836	41.6824	51.4327	22.7431	40.7902	49.7359	-4.1052	48.6524	53.2381
1.319	31.2898	46.1958	26.0458	33.7497	48.1217	1.2951	42.2349	50.5619
3.6785	37.1431	50.0858	29.2732	36.1139	49.4052	3.009	42.5133	51.3093
-0.2611	34.2458	47.5568	24.0544	33.211	47.6076	-0.5653	45.9722	52.15
2.3743	40.6773	49.2748	28.8499	38.0733	48.0422	2.325	50.3794	51.5996
1.0817	32.7464	48.9993	24.3436	37.0565	49.1403	2.0944	46.4905	51.9543
0.7183	32.7314	46.7781	26.6384	31.5133	47.0096	-0.7757	45.3253	50.7
3.4036	32.7889	38.015	25.0517	32.7327	37.1973	2.9479	40.191	38.7985
2.3002	32.438	38.8829	29.4063	27.4313	41.761	2.6095	36.442	41.2742
2.6	32.2324	43.2415	25.3185	31.8188	42.9873	2.062	37.6912	44.6291
-5.7759	37.1519	41.0499	17.7008	41.158	42.7713	-6.8237	42.3492	44.4586

x4	y4	z4	x5	y5	z5	x6	y6	z6
12.0295	31.8453	34.7798	-17.4586	30.3367	9.7013	23.6965	33.9738	12.3351
17.9984	41.7637	36.1313	-19.0191	35.2698	17.3287	24.9862	38.6981	9.6052
22.3184	28.4923	34.4136	-6.474	37.2947	16.3725	31.6532	32.5401	12.4232
15.6818	45.25	39.0645	-17.3198	41.6021	16.937	26.4846	45.9551	12.4377
24.6035	32.2914	36.8508	-8.3804	40.2718	16.6905	31.5706	37.0528	9.6516
26.103	33.7902	33.8896	-7.5058	43.2342	18.9539	34.1195	43.2849	17.9135
30.7468	30.9038	38.7537	-13.4135	41.8891	22.4898	35.0444	44.5934	20.6195
11.9132	31.5934	32.0693	-18.6733	29.4014	16.5928	17.4678	38.5253	18.148
19.9475	45.9648	49.4714	-15.235	49.7023	23.6491	30.6931	50.3701	25.8753
23.824	37.4188	51.8243	-12.273	44.7202	16.6429	39.1178	43.0288	17.8591
14.1489	42.682	43.1352	-16.0022	38.0531	20.4299	24.9495	40.9156	22.0928
15.8628	44.3439	49.6853	-20.3474	39.0113	19.6329	26.3493	44.1154	22.5785
25.6886	34.1484	37.7716	-8.0966	46.6663	19.2707	33.6213	42.6557	20.2293
23.1896	45.7344	45.2713	-9.9958	42.0526	17.9975	32.8765	41.5371	16.8156
13.0764	37.3103	36.9468	-15.8331	36.4373	16.441	24.1248	38.7289	19.1395
15.8923	43.282	44.0878	-14.3406	43.0982	20.6195	28.66	42.5475	21.0989
18.1408	42.5403	39.3601	-14.6392	43.5304	15.8374	28.1983	41.9574	16.3255
19.0594	43.4549	40.4725	-12.9546	42.973	18.802	27.6328	42.3661	19.0199
21.6979	43.2309	47.1837	-17.3419	47.7045	18.5523	33.2376	52.7594	19.2491
28.8492	48.4442	48.0023	-8.8842	52.1464	22.9898	36.2951	48.1335	21.5554
21.8888	53.0052	54.8715	-17.8129	57.9572	24.7778	35.8783	59.4568	23.5734
19.0734	28.835	44.8922	-13.6481	35.7499	15.3099	28.817	34.852	19.1529
15.9478	14.1016	45.7218	-11.5548	25.9506	19.973	26.9212	28.3995	30.0239
22.2699	18.4373	55.1847	-13.2473	26.3143	21.4996	31.9948	26.5918	23.4705
26.7547	9.1188	48.624	-11.7709	21.7121	23.3758	34.3198	22.0029	22.2857
19.9943	14.3088	55.2825	-16.5477	26.808	22.0209	35.3435	25.9188	24.8597
18.3351	41.4287	42.3339	-20.4882	51.1248	15.4042	31.7075	54.3347	19.433
22.113	45.0074	51.9212	-17.9124	49.8093	18.6152	37.4844	49.0821	20.771
17.3384	45.9131	51.7906	-20.3773	50.6265	25.777	30.1032	50.9348	25.8407
17.3577	44.5663	48.5201	-18.5238	44.9461	23.0914	27.8796	51.6158	22.3227
28.2063	41.5359	48.3245	-9.3151	50.3938	21.4348	41.5124	45.6709	19.7032
24.0294	36.5397	44.3839	-7.7212	44.5549	11.8685	34.4377	37.7626	17.8565
21.7285	49.4562	52.5013	-19.3237	49.5375	15.7126	33.2593	48.0654	27.364
13.8959	46.4713	55.1086	-21.5903	51.9652	19.1614	29.8758	52.3796	24.5479
26.3398	51.7141	58.1534	-18.2991	46.5869	16.8375	43.4982	48.7128	19.9071
24.1441	33.1331	46.5789	-11.4769	34.6631	16.8278	33.8544	33.6421	15.4567
23.3631	36.4945	40.5342	-8.3419	43.2256	19.3264	31.8461	43.2383	16.557
27.1058	37.2601	44.218	-11.343	40.6129	14.5722	39.0955	40.7965	16.3548
24.1632	21.397	33.4279	-4.3698	28.5382	11.8079	33.6791	22.343	8.4312
22.1194	38.6822	47.5799	-12.5969	41.0173	17.6771	36.6474	37.0968	16.0559
18.8753	31.4009	35.3251	-19.3072	33.9269	17.6029	27.0571	38.9709	15.4309
25.4121	30.0964	49.1559	-20.2736	38.3435	20.4712	32.3414	43.4074	20.6349
27.6592	4.0881	2.935	-11.3042	22.2229	-20.6788	37.2247	23.2669	-20.6252
27.0452	7.0154	6.293	-14.1262	20.0955	-24.8216	39.9568	25.4826	-24.0131
19.8839	33.7093	56.4882	-7.7625	24.9366	15.5046	34.7952	28.0709	20.2878
17.1364	30.8936	37.7114	-13.0119	29.2048	8.9747	30.076	30.757	12.4653
18.3787	31.1531	35.1064	-11.1744	29.7174	8.3109	29.715	33.6697	10.9604
18.0858	37.3722	37.9869	-10.3757	31.4466	9.2992	27.4611	32.4526	10.3602
18.2464	25.9457	31.2292	-5.7475	26.1975	9.7997	25.6179	24.8528	10.5832
17.7989	34.6567	36.2141	-15.4893	32.6276	10.0797	29.103	36.6736	10.6245
15.0284	27.7883	31.0208	-10.832	27.9261	9.9941	25.5497	27.9702	13.3007
16.8782	3.688	2.3248	-9.3791	9.1079	-14.2718	25.9353	12.975	-14.3149
16.1533	45.8771	49.3392	-17.6715	46.0725	12.0887	28.8129	45.4165	18.2345

16.9021	46.2606	46.136	-19.6339	45.1924	18.9371	27.6061	49.3322	21.8586
16.209	51.1634	47.8261	-22.771	51.51	16.612	22.6624	56.9452	15.7497
12.8131	43.1805	46.8686	-23.8682	45.4249	18.3989	23.8381	52.7919	22.0777
26.5562	49.9437	50.8726	-13.0178	54.2858	14.1368	34.3379	50.3255	22.1289
20.8651	12.6008	6.0993	-11.9428	22.2648	-22.4429	35.9869	26.1714	-16.0923
35.8623	40.1564	44.5119	2.5862	58.7988	18.4267	50.5319	43.8929	16.1399
35.8517	44.834	50.9618	-6.5001	54.8451	19.6689	52.4379	42.5276	19.8183
11.859	53.2741	44.955	-22.7129	46.9919	12.2573	27.0973	54.5298	18.1211
39.262	38.6756	59.3972	-3.4831	55.933	24.5495	52.474	42.1021	24.128
30.2177	33.5937	50.7434	-5.1109	50.9782	16.5563	45.4634	39.4436	17.8994
21.5693	39.6411	52.0837	-19.5409	46.9464	17.0652	37.4242	48.1324	17.5086
24.8324	50.2253	51.6192	-15.643	53.1131	21.9842	33.3603	48.6722	18.9701
26.6235	43.1973	52.0252	-10.5484	45.0398	18.7623	38.5812	43.0291	18.7667
30.1197	41.6471	50.7123	-12.4284	47.1024	17.6973	43.939	43.1081	17.1484
25.1802	42.1175	51.0012	-13.9159	48.1824	14.396	39.1612	44.6098	15.7733
31.4766	48.1883	52.6123	-11.0778	55.6982	20.345	44.7903	50.0305	16.0344
25.5144	48.0893	49.9153	-14.578	47.146	21.5423	36.8053	46.365	17.8999
28.4623	42.5815	51.0647	-14.4165	48.9361	23.1379	40.7091	46.0911	20.8022
25.408	37.1765	37.3747	-11.2818	35.7822	12.1051	35.8612	34.2602	9.6865
29.8591	33.1867	42.5618	-7.6806	46.307	14.0734	44.3759	40.6957	15.2689
25.5837	35.7878	44.1763	-12.9571	41.7627	17.3528	40.1881	41.4029	16.2899
16.9656	47.6953	44.5741	-23.8578	50.2622	14.7237	32.9571	56.9736	15.1749

x7	y7	z7	x8	y8	z8	x9	y9	z9
-37.4448	86.044	6.7023	23.3292	93.7309	18.5316	-5.217	69.3863	51.9599
-45.3156	86.3832	20.2369	21.9093	99.0848	14.5894	1.1979	84.7965	63.8054
-15.8362	91.3186	12.2763	45.9216	88.5568	16.1207	17.6396	69.8617	57.3953
-43.0416	101.2708	18.8547	30.0112	112.0165	21.557	-2.7499	86.9807	64.8929
-21.3724	95.4879	16.6247	43.6501	87.8894	13.9733	13.5846	73.7741	60.4761
-24.137	93.7846	16.6579	51.7132	90.3464	12.657	15.3311	84.6722	67.2343
-33.5119	102.2709	19.062	50.3662	108.4726	17.1556	10.2249	91.8526	76.7937
-43.763	68.0766	10.2997	20.0575	85.1013	13.8549	-9.5892	57.1426	63.4504
-31.017	108.3826	21.5407	46.4222	105.524	26.8878	3.427	88.8033	86.9144
-23.5233	121.0244	20.879	56.6297	115.7199	25.2994	10.3176	87.6572	91.8826
-29.8126	80.704	16.2869	34.3223	88.5091	17.2848	2.9885	74.697	77.293
-43.4104	98.772	21.0303	34.3428	97.6366	21.0355	-2.3412	74.5221	77.9308
-17.4381	103.3995	16.8915	52.8183	95.325	17.184	15.1504	82.9357	66.7627
-24.6318	99.1559	16.6684	50.6178	98.1364	15.8604	12.5611	76.6341	70.4181
-35.8701	86.0492	13.309	37.834	93.8202	17.0351	0.1957	76.2512	60.8124
-28.0472	83.6869	14.4206	42.2148	84.1881	13.3118	6.8047	81.486	67.4599
-27.2523	97.8502	12.773	45.2334	95.1649	10.8278	9.576	85.1659	64.0147
-26.8589	94.9764	13.9908	43.1295	93.6022	14.4436	7.6892	80.0374	60.2373
-38.3428	116.0492	20.2093	40.2324	120.0608	22.2404	2.2825	86.0032	90.6835
-16.281	115.4563	22.987	59.3142	108.3138	17.5748	22.4258	86.6876	76.4279
-32.9831	119.3624	22.0573	46.5384	118.5625	23.0113	8.3575	85.2754	72.7847
-25.0013	93.5682	12.8924	42.3706	94.1188	16.0018	5.1626	69.6057	68.2088
-21.8884	70.266	11.65	41.9766	76.8154	20.8649	0.081	63.7814	80.5511
-27.7513	82.4876	16.3994	43.5929	81.2514	14.4103	8.7595	78.13	76.7026
-29.9713	69.3797	18.0432	48.3429	74.6065	15.7665	11.957	73.3262	82.1339
-28.733	79.2059	15.2875	51.1183	73.1377	15.2569	5.8768	60.9756	81.4307
-37.6132	100.5868	13.1138	44.807	108.8981	16.6472	2.1646	101.2657	71.9846
-31.1738	108.1629	11.1009	52.331	103.1745	13.2449	7.7811	96.1514	76.5464
-36.3447	109.2606	9.7905	44.9436	105.9427	12.4929	1.9018	95.5202	76.6976
-39.6866	93.8742	9.0562	36.8494	99.9603	8.9089	0.043	88.0304	74.7296
-18.5296	115.6824	15.9338	62.7642	106.2184	17.6105	20.776	97.2454	71.5529
-15.9521	105.8678	6.3648	64.4292	96.5475	11.2665	17.579	89.3804	67.9572
-35.1629	117.1446	12.9826	51.7769	119.0448	19.8581	3.5482	104.1734	76.2842
-35.181	117.0777	14.9396	45.3722	118.5748	26.6319	-0.9514	101.544	75.7273
-33.7356	113.6187	14.8142	57.3846	114.485	14.0083	10.5784	104.6549	78.86
-23.2784	85.9934	12.8977	46.0786	82.361	11.1784	12.3128	75.8667	79.4648
-21.4819	92.127	10.2907	45.8267	92.2505	8.497	13.6277	86.2361	68.6187
-24.8795	104.1377	4.2122	52.1184	101.863	8.2071	9.6307	87.2842	69.8347
-9.1136	75.7365	8.3202	51.4011	66.6168	4.3632	21.9358	54.2671	55.7892
-21.9266	103.4995	16.7576	53.856	99.9888	15.0819	13.5371	78.9096	75.7725
-45.0957	93.5619	17.7967	27.0842	105.5448	13.7772	-4.1843	78.326	65.4604
-42.3591	104.3441	9.0693	37.3587	111.2508	9.1913	3.1965	74.2736	79.9819
-29.2359	72.8591	-10.4984	50.4747	73.3522	-8.4252	11.3739	48.4988	38.7785
-36.9035	72.8336	-16.9186	48.0156	82.0312	-17.3943	7.0224	43.3583	40.6371
-25.1211	83.8161	13.4228	41.8137	93.2853	18.8562	3.9228	60.5682	78.5685
-24.6067	69.3906	2.4235	39.9234	71.1075	6.1236	6.2399	50.6349	52.9743
-28.8994	73.5785	1.3737	38.7222	81.603	3.1476	3.2755	65.4807	50.1374
-23.7494	82.5844	4.2038	37.8177	82.1319	4.8224	5.7996	66.9193	52.0317
-15.7639	68.6295	2.8609	39.0831	62.9758	3.7324	9.732	47.1609	49.1258
-34.315	79.6938	5.5831	37.0907	86.7897	7.6355	1.4465	64.9174	53.3526
-20.4227	64.8754	4.6614	34.3116	68.0242	8.5561	4.846	53.117	48.0455
-24.6544	40.1229	-12.6598	34.155	47.9311	-11.562	6.2015	20.2794	25.2533
-33.949	102.5067	9.8978	47.1811	105.8051	19.469	1.1801	92.3148	67.9979

-39.8586	108.7025	18.3155	35.8417	116.9408	16.6956	-0.2099	100.1754	70.5534
-49.2378	115.6661	10.004	35.6012	127.0257	6.3948	-3.6998	108.3266	76.1064
-43.6222	101.7992	12.1662	34.3468	107.368	15.9009	-8.6254	93.4744	71.5571
-21.3786	123.1779	15.4021	59.3949	114.9147	18.0538	14.6665	103.0171	78.6368
-32.5583	70.5581	-20.4643	46.8499	80.8305	-6.6295	-0.4207	53.5804	38.4706
5.2351	114.7181	14.0132	80.61	94.1433	12.1503	42.5572	95.655	71.9667
-7.6346	107.4006	20.7482	72.8709	90.6556	17.2403	33.2131	84.7044	75.4635
-46.206	104.4102	9.4679	34.2026	114.2825	15.7004	-8.4523	92.9232	67.7786
-2.642	129.4573	25.7653	86.8668	109.0915	20.5781	37.7162	92.7772	92.2
-7.9116	109.7026	15.7399	79.281	91.21	16.743	30.1464	87.6118	76.4352
-34.9251	98.1322	16.8306	50.5548	108.5662	18.7694	7.7933	87.6807	78.3768
-31.3217	115.8308	16.0595	52.7353	114.642	16.0028	11.5503	103.9742	77.0194
-23.4133	113.9884	18.3851	57.1036	108.0685	17.6331	14.6225	93.8769	76.8085
-15.1879	113.506	17.0089	59.306	106.3829	16.664	18.2436	88.6508	81.6025
-28.9706	103.678	14.9116	57.754	104.4847	16.3554	12.9435	88.0084	73.1
-18.9883	113.6466	10.8177	67.9171	103.0805	10.2815	26.1136	104.8844	81.5189
-28.3467	106.0271	17.046	53.3512	98.3669	10.9754	16.5756	96.6305	79.0562
-27.2548	104.2657	16.4128	62.8061	102.9946	14.2074	18.7329	93.6194	76.1605
-21.1449	79.402	8.6435	45.1573	79.8896	5.2032	14.5112	69.395	54.121
-16.9484	109.6111	7.7849	67.5898	97.1024	7.4568	19.9914	84.6217	65.8804
-25.9623	105.6755	11.9852	52.032	105.2717	10.0147	13.9724	85.392	68.4375
-40.1442	107.8555	8.744	32.5611	117.2251	10.8598	-2.4841	96.024	66.6787

x10	y10	z10	x11	y11	z11	x12	y12	z12
-10.8188	40.6323	37.2128	11.7396	45.425	40.7417	-45.3356	65.0637	34.6727
-5.5486	52.6147	41.9404	17.3925	56.804	39.6084	-41.7188	70.2259	47.5357
4.2265	47.855	39.2164	28.5835	44.1438	39.1162	-21.1755	77.519	41.8836
-13.5245	61.7736	44.5288	18.5798	65.8014	43.8518	-42.1241	77.8019	46.2457
-0.2451	55.3896	45.3882	26.1836	55.1152	43.2683	-24.6623	73.9752	45.6233
2.2785	56.9338	45.2119	25.3068	55.653	43.7474	-26.9418	83.8765	50.1171
-4.3384	59.3822	52.9419	27.9819	60.3112	51.992	-36.6839	87.6244	56.0596
-17.1017	41.0098	38.9498	8.2203	47.0644	40.0802	-48.7809	54.8204	39.0472
-4.4936	60.6234	52.6304	17.5813	59.6226	54.043	-37.392	92.6807	54.3525
-4.9168	61.4541	57.96	28.3716	59.4722	58.9598	-39.3551	101.1723	57.8622
-7.8761	48.8665	43.9087	16.0647	50.478	44.9386	-36.4091	72.379	49.5718
-16.1137	50.0126	49.1729	17.017	53.5499	50.0117	-49.2078	75.4461	52.4894
1.8893	60.5979	46.4555	27.0117	58.4877	46.4744	-26.2205	86.5029	46.5555
-3.7459	55.5747	45.1602	26.3042	54.4333	45.2091	-30.8804	82.5587	49.096
-7.6617	50.7938	40.783	14.1901	50.3334	40.7067	-40.0051	75.7877	44.6856
-5.9604	53.7521	45.5656	19.9221	54.6983	44.6292	-33.8503	83.5869	49.7547
-5.6065	55.9551	42.575	20.3596	54.2602	42.2337	-33.9164	83.9634	45.3685
-4.6006	52.0544	42.3351	19.3583	53.2953	42.6026	-35.714	83.7661	44.485
19.5014	64.392	57.2895	-8.8664	61.026	56.8581	-47.4399	91.9819	56.2177
3.2996	67.4112	57.5101	33.304	65.0433	54.5116	-23.7911	95.2589	61.2902
-8.4072	76.8462	62.1164	24.4811	76.7563	61.3167	-38.8012	99.9041	61.9504
-8.5679	47.0265	49.4183	22.0307	45.3532	50.966	-29.9582	75.4852	44.9788
-6.5326	31.2725	60.5335	15.4879	34.2468	63.3671	-32.2603	55.009	47.8098
-3.9269	34.4727	60.4061	26.1911	35.802	59.6893	-31.7425	62.9206	52.8003
-1.7448	24.6799	65.0864	27.9226	24.7865	64.1875	-32.9113	53.0466	55.1236
-8.8162	30.2889	59.5745	22.4506	29.2082	60.1285	-34.0281	64.3071	54.453
-14.6405	81.7615	55.9141	21.3622	85.4521	57.3637	-43.6245	98.131	56.7691
-18.316	90.7352	61.9898	36.8981	89.9286	64.545	-38.5578	100.6098	52.2138
-24.7608	91.1273	64.373	30.2657	92.2882	64.9307	-47.3628	99.6193	50.2215
-18.6702	72.5856	58.4644	23.8423	78.7356	57.9283	-44.8107	83.8288	50.253
-0.6003	87.5824	61.8978	42.151	85.6801	59.8242	-23.9545	105.7995	53.9822
-1.6823	60.3745	42.8008	30.4924	56.1468	44.7715	-27.1611	89.6627	45.5323
-11.9722	70.5578	53.4567	25.7948	73.0638	55.4652	-41.8025	102.1493	54.3729
-10.5975	65.659	58.4701	15.5204	68.1591	59.1963	-45.9298	97.3005	54.8943
-6.9626	69.7153	57.2188	28.7946	67.8245	58.1908	-38.5217	101.1898	59.9396
-8.9502	61.7429	54.8447	36.2844	60.8037	54.2916	-27.5536	74.7498	43.8728
-4.4386	70.4189	53.1756	30.9426	70.4868	51.1916	-25.4158	83.5862	46.653
-9.6345	63.2531	45.2117	33.4354	63.2184	48.1233	-36.0142	92.9949	45.3977
6.2772	38.6161	36.0016	29.3962	34.5978	34.5718	-15.8225	65.4388	37.3565
-4.5021	55.7608	51.7704	28.4213	53.6429	50.0707	-27.2906	84.9727	51.9689
-12.8917	50.5227	46.4881	15.4815	54.4619	45.6458	-41.3115	69.9173	50.9611
-15.7695	54.3427	57.5433	24.6543	58.812	57.8337	-46.5916	84.7915	55.3473
-10.0768	40.2069	23.3954	32.6907	39.1905	24.4716	-34.7607	59.078	29.02
-16.9102	46.0597	31.0595	31.8193	49.4633	30.6111	-38.699	58.357	22.188
-3.5501	38.1144	53.7928	22.657	41.5225	55.7472	-34.2573	68.8693	45.9819
-9.5843	43.0747	36.5461	24.8455	45.0043	38.6909	-28.6987	58.4943	36.6452
-5.6065	44.943	35.7637	19.6474	48.6604	36.5601	-33.9871	61.8856	35.0506
-7.3345	47.6683	35.7923	22.2925	47.5625	36.2124	-29.0884	67.8457	35.277
-3.9634	36.6371	32.2905	23.5674	36.2813	33.2806	-20.8714	52.8508	27.5826
-8.7724	46.1485	38.7883	17.3621	49.5919	38.6499	-37.8926	61.9951	39.2517
-8.2169	39.2741	32.604	20.113	40.054	34.5081	-27.4228	57.1973	33.3403
-8.969	19.1959	14.0954	21.756	21.6242	14.3879	-26.8428	29.3037	9.8221
-13.6384	62.9301	46.5708	21.1726	65.4043	50.0901	-42.9905	88.0868	50.783

-12.6696	65.87	48.6916	18.1037	71.9903	47.7787	-38.3132	92.1013	55.0653
-13.2385	67.675	53.3024	15.8118	73.8921	50.0561	-49.1258	97.2161	55.7846
-18.8328	62.844	49.3242	12.9983	66.7292	50.2909	-48.7254	86.277	47.8599
-3.6608	76.6428	53.5286	30.9194	73.2343	54.8374	-32.4795	109.9057	53.9591
-17.4775	42.4	19.4553	24.2817	45.1224	25.1645	-43.1614	54.969	14.149
16.5173	88.0319	56.8568	59.9266	76.0994	54.3877	-2.2596	106.9439	54.7593
8.5005	80.3174	64.2384	51.7713	71.5258	62.5851	-14.918	98.3215	59.0066
-28.1996	78.9098	51.7186	16.8468	83.9685	55.0473	-51.5391	86.1571	46.6553
12.1571	88.6782	75.6105	60.4076	79.4722	75.3359	-13.5833	116.5506	72.8876
5.5416	67.0471	53.0758	45.2726	58.4927	53.2658	-13.9795	98.9865	54.9514
-6.7582	58.768	54.9124	24.8369	60.1658	56.1657	-40.3316	90.2175	57.8941
-4.1485	67.0712	58.6786	24.8455	66.3241	56.1156	-35.7825	106.5542	59.2001
-2.6046	62.7569	55.1756	30.6055	61.86	54.9931	-31.3988	97.6952	62.9116
3.5725	58.3931	57.891	32.6243	53.6209	56.336	-27.7385	98.2576	53.7781
-12.1824	79.7341	60.2565	39.9603	76.3925	61.5308	-31.6782	92.6706	59.859
-2.9517	90.4932	64.2578	49.0791	86.2138	66.1694	-23.1333	107.9199	56.2446
-7.9257	84.4433	65.2763	39.4416	83.2009	62.2864	-32.4746	97.1772	58.9363
-5.3815	81.3275	61.2773	39.2498	77.5262	58.6373	-31.903	96.8483	56.808
-2.2221	50.4368	40.8115	28.9615	49.3	39.2306	-28.873	72.7823	49.2965
1.8136	56.7284	47.6694	33.1669	53.4535	48.9094	-22.5233	91.2769	48.7908
-1.742	61.1183	48.384	28.7092	58.4299	47.7777	-29.1806	87.1632	50.9987
-12.4874	67.0501	48.8394	15.1808	71.8035	48.8327	-47.8201	87.8281	55.0215

x13	y13	z13	x14	y14	z14	x15	y15	z15
26.2091	75.7858	51.135	-46.8372	76.7327	51.2869	23.3909	84.2588	64.1997
32.6118	82.068	41.3935	-38.8602	74.8108	62.9199	37.0531	86.9576	50.3451
52.8252	66.1919	40.4365	-18.2606	80.0896	55.8244	54.0831	70.3801	53.4615
35.9174	88.0194	52.3225	-43.1329	83.4063	61.8695	35.9741	93.9534	62.035
47.1667	70.8003	44.5098	-22.3847	77.4305	59.7628	47.4227	77.366	58.2444
56.658	82.8994	47.5606	-26.1584	86.595	62.4526	56.5195	85.7016	60.8924
57.0231	90.1554	52.3628	-35.4067	92.4366	70.9219	56.4766	95.4029	67.0992
29.1415	74.0924	44.5278	-49.0132	56.8813	54.7218	27.3684	74.189	58.4952
48.4832	90.8056	60.352	-38.1062	97.4136	69.3987	47.2996	95.2372	76.2318
66.7661	97.1101	61.9126	-37.6039	106.4102	74.936	64.8855	102.5701	80.6489
42.3898	78.6687	51.0456	-34.487	79.8394	66.2243	40.5015	81.5947	67.0397
42.8512	85.8711	53.2382	-49.668	80.2407	68.8435	42.2488	89.1153	68.6559
57.6673	80.295	46.9415	-24.9125	90.2974	59.752	57.1697	83.1189	60.7757
56.772	82.0581	47.1048	-30.7155	88.0396	62.5937	57.4289	84.8584	61.7038
40.3627	80.0236	47.3681	-37.6024	78.4631	59.7671	36.5866	83.6423	63.4211
47.7102	82.0275	47.2585	-33.5768	88.7204	63.6077	47.2555	87.1303	63.1233
51.3507	77.9369	44.2794	-33.0305	88.1987	59.6026	51.692	82.1791	58.6333
49.4306	81.1817	46.349	-34.3398	87.1599	60.5713	49.4765	84.8692	60.5159
50.5177	102.6699	57.6701	-48.2585	97.6042	72.0258	50.284	106.4136	76.7375
66.2448	84.965	50.4574	-23.5244	100.097	73.2091	69.0287	90.213	63.6355
52.4155	102.6441	59.9882	-41.2246	108.4378	79.2368	53.3775	109.0503	77.1803
44.6431	73.1074	47.2573	-31.0547	79.4854	55.9491	46.2942	78.3181	59.6276
42.1392	61.5812	55.2745	-33.2548	60.6303	60.1888	41.6179	66.6671	65.8779
48.7934	68.1155	51.7322	-30.8374	69.8638	63.7248	47.9108	74.9115	63.4926
55.1768	58.4804	51.7648	-31.2953	65.0097	71.9591	55.0221	68.52	69.2445
53.7851	55.7775	57.7268	-32.2962	71.2588	65.4522	51.6777	63.5003	67.6046
48.8428	104.8557	58.6574	-42.8231	104.8304	70.6586	45.7028	112.2643	74.6993
59.532	99.3566	53.3697	-39.4678	106.6859	67.4807	57.8686	105.5316	70.522
52.5416	102.6944	54.2244	-46.065	106.0235	67.801	50.9007	108.2791	70.953
45.2996	95.4295	50.2484	-43.5547	90.0131	68.3583	43.4479	101.681	67.5927
67.4837	97.0403	55.0957	-21.4765	110.435	70.1097	67.1527	103.0187	70.041
61.9978	78.409	51.6773	-27.5947	96.456	61.8552	59.1317	82.8514	67.066
48.4016	103.0195	62.8755	-42.7587	110.4617	70.4264	47.7639	113.3379	80.6853
42.587	99.2046	66.1582	-48.2493	107.3897	71.6494	41.3609	108.8671	80.5655
62.7977	98.3899	60.2957	-36.0716	108.0303	79.8685	60.0229	103.8066	73.646
54.0002	73.0663	42.2127	-29.8211	80.2749	60.9797	57.321	80.2292	64.2614
54.2399	85.7427	44.3349	-25.2477	89.4605	61.9951	54.5728	90.6639	58.5002
58.0371	91.3648	50.1376	-38.3102	99.0027	58.0519	59.4983	96.4155	64.6212
58.759	53.776	31.0811	-11.835	68.7392	50.4671	58.0426	57.5716	44.811
57.7306	76.9784	50.4123	-26.3353	92.0186	65.4994	56.8268	86.2379	65.3336
38.4942	84.0098	47.7703	-42.8516	75.3663	66.3037	38.4738	88.9425	61.8579
49.8292	98.2609	53.0114	-47.1263	91.9085	69.6424	47.5549	105.0591	68.7644
58.7787	59.0481	29.148	-32.8684	61.1858	38.816	57.1073	62.532	38.2919
49.9992	64.3008	23.219	-38.8556	60.8286	36.6344	49.2433	67.7324	36.7683
47.6163	76.0551	51.2545	-35.156	73.111	63.2728	43.5155	80.5763	67.7961
42.1855	60.5718	38.4279	-27.1662	64.2443	47.3441	37.8839	65.1639	51.4504
44	69.9986	36.7316	-33.0128	65.7688	44.8956	42.1212	72.4399	49.0151
42.7788	66.915	36.3481	-27.0352	73.6014	53.1662	41.1246	70.7976	49.4409
42.3447	50.4809	28.7863	-19.8961	54.4435	42.086	40.7415	54.5905	43.1129
41.6128	71.0077	40.4119	-43.2784	71.4193	55.2496	38.8462	75.6589	50.6613
39.864	57.7143	36.2402	-27.0474	59.8425	44.1226	37.8854	62.0756	47.7986
38.505	33.825	11.1399	-28.3168	28.6495	21.5065	38.6078	34.4224	23.9363
46.2801	90.3672	60.5734	-43.238	93.9429	65.4362	40.8613	97.0831	76.4118

42.8639	98.8675	50.8626	-42.2824	98.3198	69.9456	39.0956	106.0766	70.2043
40.307	110.1276	54.5165	-51.0392	102.1957	71.5356	40.8688	119.1077	73.4265
34.7853	97.5403	54.1882	-51.0663	91.7689	62.039	31.0347	101.696	68.1676
58.7595	99.5984	58.0738	-32.4209	115.8086	70.6324	60.9157	109.0083	76.1045
48.0676	62.862	29.4607	-45.3511	57.5499	30.2635	45.1191	65.1519	44.2806
86.0356	80.1316	48.9891	1.4579	113.6544	68.7663	86.6239	88.9876	65.9987
80.4262	79.8492	56.375	-11.3643	104.2333	76.5123	81.2252	86.6465	73.452
40.8718	100.3715	51.3278	-53.4992	92.7477	62.3033	36.7805	105.5895	68.485
93.8839	93.5528	67.5118	-9.8335	122.1005	87.3515	95.4171	98.9304	81.8675
80.9217	78.7323	56.6083	-15.1765	105.0041	71.4443	80.2501	85.3995	72.9368
57.0443	93.1033	60.6003	-38.8299	95.4677	71.6366	53.8487	98.2064	74.0669
55.542	101.3496	59.831	-34.3648	112.3721	73.7252	54.7083	111.3027	77.9833
63.946	93.299	63.0304	-30.746	101.6725	71.7776	63.9981	99.2827	74.543
67.9461	88.6786	53.9766	-28.1553	104.0172	72.401	69.9626	93.6123	72.1337
62.8846	88.135	57.3224	-34.946	98.7387	72.3358	62.8243	92.9767	70.4867
74.6992	97.9486	55.6743	-21.6697	112.7225	72.2992	74.9608	103.1334	72.6685
62.8436	93.4384	54.3596	-28.4577	102.4012	77.5431	62.5603	99.2097	71.0426
67.4042	88.6325	54.2746	-28.8028	103.4618	72.1445	67.7378	96.3172	70.0054
57.2313	74.1844	44.3076	-23.465	77.717	60.6415	53.1893	78.599	56.1172
66.2237	79.3221	50.2059	-19.9452	99.0236	63.9022	65.7842	86.2783	64.2273
58.5853	87.0815	45.4414	-29.8909	92.0204	61.4312	58.5458	91.8202	60.5264
43.6866	101.1154	56.0783	-46.9658	92.7746	62.8939	41.2593	104.0313	63.8908

x16	y16	z16	x17	y17	z17	x18	y18	z18
-35.4028	88.9935	61.6414	6.8855	96.6287	69.9918	-56.2694	107.4355	14.2199
-21.5516	88.6985	70.5432	22.469	97.409	66.5225	-60.5281	104.1352	30.3712
-4.936	84.7723	65.6688	34.3994	83.6347	69.6442	-28.1823	116.7436	25.5717
-26.9882	100.3235	72.4532	16.5193	105.1759	72.8646	-57.2177	123.3238	27.0604
-7.2084	90.4884	69.4573	36.1641	85.6967	66.0544	-31.9032	118.2316	31.8859
-7.4495	95.4199	72.3152	39.2582	95.3424	71.2438	-33.8991	121.2273	31.7808
-13.7631	103.9776	79.698	35.2939	105.3464	77.9097	-49.1507	128.5696	36.8229
-35.6689	65.0127	67.7271	11.1725	75.6375	69.9531	-60.7181	88.2431	25.1525
-20.0057	103.3559	83.259	26.4782	100.501	86.8622	-44.626	128.5182	32.9271
-18.2457	108.0611	90.0145	39.5009	105.5176	94.6214	-39.2502	142.2182	30.7662
-17.8175	86.6261	80.5249	22.9296	89.2185	80.4118	-46.2311	106.2947	29.9568
-31.2148	88.3503	80.3794	22.3029	94.0661	80.4018	-59.6917	116.6582	32.1842
-5.4542	97.6597	70.6279	39.0696	93.7449	70.8585	-29.5928	124.036	28.7704
-11.4644	94.3872	75.1469	39.979	93.0948	75.2136	-35.8602	124.9952	28.1655
-22.1512	90.8087	67.3212	18.2627	94.0605	68.9845	-48.3559	109.3702	27.4854
-14.1068	97.3819	73.1826	28.6752	97.4287	72.3366	-42.808	119.6138	29.5364
-13.4219	98.3406	68.6151	33.9197	94.5272	67.3463	-41.2074	119.1448	26.8195
-12.8749	98.6138	69.3128	28.9866	97.386	68.5304	-40.2553	117.9393	29.359
-29.5171	101.4867	89.0051	29.7957	107.1165	89.8641	-58.9091	134.9748	33.6045
-1.0947	105.7697	83.9824	50.992	97.5412	76.8929	-30.6851	135.1128	37.5883
-19.9055	108.6022	86.2402	34.646	108.201	82.8125	-52.5499	137.8999	35.3639
-16.187	91.6023	67.5513	29.6681	91.7181	69.2664	-35.9172	109.2722	28.6363
-20.3131	73.9291	72.5151	24.6987	77.1825	77.6317	-39.919	86.8637	27.1935
-15.3879	78.1827	72.8458	31.4086	80.6652	71.6243	-42.4338	96.6557	27.7762
-14.0091	75.3182	78.7253	35.7777	75.6491	76.6834	-43.2811	91.4442	32.8921
-15.1421	76.0805	74.8784	32.5328	74.2451	76.6699	-39.4595	99.0963	29.7705
-19.2057	118.9829	80.8603	21.6649	123.0494	82.1319	-56.4343	133.9274	33.1459
-18.9084	117.6617	80.5028	33.4763	115.4768	82.7469	-48.1481	136.4192	26.089
-25.821	117.6415	80.9221	26.7639	119.2395	83.4047	-55.4544	134.9708	27.094
-25.8137	103.3538	79.5652	22.3402	109.7082	79.3739	-58.2737	117.7541	28.381
-0.0813	119.6037	80.532	48.1645	116.3059	80.8132	-33.3022	146.2007	32.5676
-4.9482	107.9287	72.0956	43.2753	99.5145	74.848	-31.6655	132.9234	25.2016
-25.7597	119.5845	81.1037	31.7441	119.2636	85.7645	-51.298	146.3392	32.9238
-30.4698	117.1577	78.3775	22.5357	119.1655	86.8649	-56.5231	137.7858	30.0401
-11.8994	114.9479	81.2281	36.1111	118.1037	82.5396	-49.8188	141.7721	38.837
-10.2014	89.5521	79.4048	40.1298	90.4268	78.7874	-33.0369	109.7886	25.9767
-8.018	99.0472	74.8392	37.9933	100.3709	73.2494	-35.056	117.2073	26.5891
-18.8305	109.4637	72.8027	41.1349	110.6355	76.941	-40.8169	128.7386	23.6316
1.6901	74.3728	63.5527	47.8156	66.9214	59.6571	-16.3164	94.342	22.5754
-8.7419	99.2645	78.5519	40.7271	96.2343	76.9234	-36.3939	122.8488	29.697
-25.2316	83.1056	71.7426	13.6547	91.0841	68.3147	-61.6776	104.6438	36.4387
-30.2516	95.7946	84.4584	29.4785	103.4389	86.3781	-61.7805	124.9002	32.9667
-14.0155	61.651	50.1942	34.2688	63.2589	49.4034	-45.7022	97.5048	10.8547
-19.1026	61.0982	56.4506	30.8648	62.673	52.3748	-55.9216	100.0575	6.6343
-21.6722	89.9091	79.5081	24.1111	92.8062	82.5826	-42.8281	100.8505	26.2738
-16.4778	68.3993	59.6781	24.0807	70.1016	64.5175	-39.0613	91.2792	17.7122
-17.2851	75.7344	53.8113	23.6016	79.2072	54.3727	-46.7645	92.3056	19.2252
-12.83	79.1002	57.4219	26.2633	79.8414	59.0842	-36.0024	101.0433	20.1445
-8.7795	58.728	55.4619	28.5604	58.6624	56.5195	-25.416	84.3874	10.53
-21.5459	77.7511	58.924	18.9936	78.0554	56.7699	-48.4433	95.5852	21.7113
-15.6782	67.5948	54.7627	22.9843	66.4558	55.8211	-34.3532	84.3597	17.4077
-15.0882	31.0651	35.4392	24.4053	33.3637	35.8243	-36.9526	60.6686	1.9048
-23.6964	109.6737	75.6822	21.0452	110.2171	81.3452	-52.3898	127.3074	27.8225

-24.9166	111.7628	76.1413	19.8464	116.4882	77.6985	-56.4081	129.4034	32.2457
-33.0753	119.8477	82.6368	21.2304	127.517	80.7359	-68.8231	135.72	32.193
-34.5924	99.7556	74.8512	13.8529	105.3038	78.4579	-61.3004	123.5307	23.8038
-10.1367	125.0827	81.0794	40.4548	120.2237	83.4488	-36.4943	149.371	30.5394
-28.9537	67.464	44.8103	23.6286	70.4129	52.6263	-54.0656	96.9917	-1.1884
21.0589	118.3192	77.7	72.0442	101.4	73.2224	-1.4556	149.7331	34.2672
12.5453	106.7158	83.5998	60.8339	97.9163	82.0093	-20.1519	139.2568	38.1603
-33.5824	102.207	70.8179	11.9161	108.2981	74.6212	-69.4954	126.6268	24.9625
17.9657	122.3508	99.0654	71.6312	110.1101	96.2256	-12.2851	165.6864	50.7471
8.0046	115.1668	82.8519	61.3624	99.0566	84.0091	-16.4912	140.4474	33.7081
-19.1126	100.3768	84.12	33.1739	102.7291	85.6157	-51.2451	129.7454	36.4962
-14.1102	121.4807	84.9495	35.6205	121.1308	84.9628	-44.4304	146.0043	36.8381
-8.1909	112.8748	82.6093	41.5756	109.933	82.18	-38.6248	135.31	38.967
-3.9206	109.3468	85.0894	46.8574	102.9085	83.6695	-34.1917	134.8882	36.8277
-14.7753	106.1021	79.1267	39.4324	106.1842	81.4206	-40.9481	131.4103	35.0629
0.0345	122.9178	84.6841	56.1167	118.1375	85.9955	-29.8486	148.2765	32.8919
-8.3941	112.8464	87.9798	44.5667	111.4401	84.622	-44.6961	135.1654	35.9783
-5.5765	109.924	81.909	46.4848	106.5437	79.6339	-42.6407	137.5617	39.2578
-7.1403	84.0829	61.6953	36.8218	82.8487	58.1037	-34.2419	107.7348	27.9183
-1.9703	103.002	75.2503	47.2083	95.1684	75.2439	-27.8761	149.724	24.2597
-12.0457	99.5625	71.5387	39.2578	100.6549	70.3752	-44.6971	126.3763	32.1433
-31.7013	101.6406	72.9049	21.7766	108.0881	72.9519	-65.0766	127.0096	31.5889

x19	y19	z19	x20	y20	z20	x21	y21	z21
33.2954	115.8063	31.5748	-55.3072	124.8317	14.0942	28.6395	132.2558	29.8655
32.3704	119.4375	21.0859	-63.5164	116.2603	27.7701	26.2684	133.1427	17.8904
62.3198	105.8124	30.5631	-24.0063	129.4792	23.555	61.4287	119.2414	28.9294
38.0154	131.1176	30.2467	-55.6628	138.2459	22.6172	32.4132	145.3593	26.1238
57.0688	107.9272	27.674	-30.6128	128.8088	27.9779	57.1661	118.3445	25.3129
63.4163	119.5818	29.7337	-29.7364	134.9059	25.6227	59.874	133.1124	23.35
62.9646	133.2183	31.5384	-49.3148	144.0866	29.0619	60.1597	150.0768	24.6547
24.8396	109.9009	28.5789	-62.5243	103.6642	22.1274	19.4085	124.3177	24.6456
59.2125	125.9587	40.0081	-42.2654	142.1267	28.7805	58.6672	139.9065	34.6112
72.7838	137.8002	37.2517	-32.7154	160.1464	27.2318	69.6488	155.7757	33.0905
48.2246	113.3822	32.2171	-47.1169	119.0869	27.5495	45.8065	128.245	28.8848
45.3376	126.9507	34.5343	-61.0477	128.5853	29.1552	45.0814	139.7171	31.4963
67.2466	116.6513	29.1228	-26.1038	138.6692	26.3237	65.5161	131.3598	26.2018
63.9593	121.2763	25.9958	-34.83	137.8171	24.6864	63.1843	134.3551	21.9816
45.9667	116.4496	30.2524	-48.1952	123.3447	23.0226	43.0789	130.0675	26.4725
56.5763	119.1872	28.5557	-40.0137	133.4073	23.4259	54.8984	132.2269	23.5365
61.8845	113.4364	25.1457	-34.0486	137.2908	24.597	57.787	132.9777	21.607
56.3236	117.293	28.9752	-37.6255	134.4411	23.5453	52.5525	133.0764	23.4064
52.9051	147.3673	36.16	-57.4352	151.8884	25.5676	50.5834	164.8685	30.2975
77.0333	124.3839	25.8354	-26.5245	149.2985	33.5487	77.2947	137.1147	24.4501
62.7191	147.1923	33.2878	-50.4232	157.6331	29.5635	61.4896	164.252	29.9136
53.1515	109.1657	31.7293	-31.773	125.4572	22.0146	49.8418	126.5072	25.2737
49.7082	94.0381	36.8149	-35.2455	104.3967	20.0658	43.825	110.7722	27.1704
52.9457	103.1215	28.077	-36.8713	117.2043	20.8733	46.3515	123.0856	22.3229
59.819	95.6577	29.5091	-37.0033	110.4389	26.0647	52.4313	113.136	22.0645
64.6953	90.4255	31.8375	-33.4114	112.0449	22.4396	63.9568	104.7066	24.5533
57.222	142.9164	35.4667	-52.3132	152.9454	28.6413	49.9074	161.3604	32.6515
68.0983	137.0607	29.964	-44.7282	149.9954	23.3104	66.5534	152.2844	26.4109
60.7954	140.4763	29.7737	-52.069	149.5302	22.7709	56.747	156.1604	24.4923
48.6749	132.2213	28.5229	-58.7939	132.4175	24.6347	45.3465	147.6089	25.1763
83.7422	133.9631	31.7065	-26.5656	164.5393	28.1174	81.5068	153.0903	28.8034
83.637	115.404	32.3241	-22.4939	151.4119	17.5206	83.3014	135.8049	25.4216
66.9171	147.1461	43.9848	-46.4966	164.0065	24.3569	64.2233	164.7936	33.9311
60.6167	142.0453	44.1874	-49.6604	154.8909	24.5455	57.1807	158.394	37.3407
75.4909	143.2399	36.0183	-44.2116	160.6181	30.1723	69.4921	161.9543	26.9775
60.3861	107.0795	22.3944	-31.3537	123.9392	23.3757	59.7855	120.9473	20.6849
61.0583	118.6075	22.6595	-33.8477	131.7312	23.1266	58.5985	133.2714	21.095
68.5045	126.9654	30.6987	-34.4005	145.2906	19.7446	62.7548	144.4405	25.3877
65.2995	81.3411	16.9821	-13.0012	108.6721	20.1825	66.6241	95.553	13.0845
68.2021	117.3637	28.9472	-33.1138	139.6975	24.2285	67.7339	135.0138	21.883
38.8966	126.3845	30.1059	-61.688	122.7039	28.4243	31.7897	141.4099	23.1957
51.3435	139.232	29.5848	-60.6655	141.6954	27.2784	45.7548	155.0275	24.1404
67.6188	102.0992	13.0707	-40.7313	117.1454	11.5215	60.0745	122.2533	13.4511
58.0769	108.2361	6.4957	-53.7198	119.3865	8.0462	53.3484	125.4324	8.3355
52.2021	110.7347	32.7139	-42.3258	120.5039	19.3145	46.6829	132.1538	25.1764
51.7011	93.1138	22.7411	-37.6538	101.7681	14.1415	51.2208	104.7355	19.6354
50.1329	103.2525	22.1658	-45.4098	106.2396	15.6947	47.893	115.8149	18.166
49.5425	100.0193	22.2339	-36.424	108.7284	15.78	50.8202	108.6756	17.1653
48.7532	80.677	13.0069	-24.2258	90.2258	13.6396	49.2249	85.9615	15.3869
46.8846	106.5051	25.1986	-48.9522	108.9455	16.1808	43.5855	118.7962	20.4668
45.36	86.0722	21.8949	-33.5401	92.5218	12.4392	45.2088	94.7284	17.5002
43.413	67.0802	3.9732	-36.5064	71.578	2.9721	40.4113	76.7091	5.0038
59.6528	132.4478	39.1776	-45.2623	148.1134	22.3349	56.4891	151.7287	32.5694

49.3449	142.5957	30.626	-56.1334	146.4717	27.0759	44.4513	157.097	25.6072
49.6035	154.2019	29.5471	-68.8844	155.2287	26.8726	44.335	171.8543	23.3319
45.7191	136.4584	33.718	-58.9638	141.003	20.533	40.1616	153.294	28.968
76.7329	136.5523	37.2719	-27.6769	170.0279	24.7756	76.8329	158.9842	30.0742
55.5334	105.1824	17.3495	-51.4649	114.8578	-2.6093	50.1474	124.2828	15.4196
105.9671	117.7208	30.932	7.9638	166.7199	28.5177	107.2434	136.1783	25.344
98.8469	115.8998	33.8886	-14.0629	153.2925	33.5694	99.1015	132.7998	30.5408
46.1005	145.0102	34.4798	-68.8102	142.3254	20.0605	42.6485	159.9182	30.3974
112.5678	135.5458	42.8619	-9.8655	180.89	39.1222	113.6287	153.1641	34.2591
99.9547	111.9002	35.895	-8.6759	153.4382	29.9824	100.2894	126.7129	30.5829
65.0285	134.7262	39.9662	-49.032	145.8327	28.6424	62.0257	151.1223	32.3458
68.9779	142.9868	35.9239	-40.8193	163.8208	31.4719	68.4469	158.4492	29.4381
73.9398	130.3785	38.7169	-36.0256	149.5143	30.7785	73.5165	144.2711	31.3274
82.5893	123.8767	34.9661	-28.4486	153.968	28.867	80.7495	143.1872	26.8736
72.817	127.1411	36.2011	-33.402	152.591	26.897	70.6773	144.797	30.3195
89.9271	136.9605	32.7145	-24.7038	164.6896	28.2074	87.7257	156.3831	26.4959
74.8828	131.0697	31.0431	-41.6144	151.6079	33.8317	72.832	148.4899	28.721
82.8305	130.8041	32.8947	-36.1731	158.0349	34.5633	78.7263	149.0041	29.2338
60.2301	108.4084	23.1592	-31.576	121.3515	22.7691	57.2101	121.0859	17.2669
78.049	138.9032	21.737	-27.1477	143.0776	20.8958	75.2375	133.5039	19.182
70.3542	127.4696	29.231	-40.6823	142.2752	28.5034	68.7104	145.1641	25.0299
51.2682	143.0235	34.3009	-61.3727	144.1926	25.0614	45.0666	158.0801	26.7578

x22	y22	z22	x23	y23	z23	x24	y24	z24
-61.367	106.5876	27.1552	32.8766	116.7692	43.9811	-56.3184	118.4045	11.6483
-63.5741	102.1971	44.614	36.0895	121.3421	33.627	-61.1785	109.4104	25.3562
-31.7672	115.4824	35.4383	65.2835	105.0867	41.2237	-23.9269	125.8806	22.3859
-63.4765	118.2176	37.6125	43.1563	129.912	42.3744	-55.4305	132.2524	22.0675
-37.4685	116.1353	39.8483	62.2637	107.0424	38.6658	-31.7871	119.6286	27.1967
-39.547	121.7072	43.4131	68.3541	121.8308	41.7548	-30.5688	124.3323	24.0716
-51.9628	129.8509	48.5999	68.7975	133.3821	41.8864	-50.0451	137.6732	27.3349
-67.6224	87.0385	34.7026	28.9868	111.7987	39.9285	-59.1428	94.8691	19.4332
-51.2006	131.867	45.6609	65.8384	128.2731	55.6843	-38.9914	135.2487	26.5624
-46.7951	141.4685	43.7817	79.2338	137.1037	54.1115	-33.7824	152.9494	25.227
-51.8464	111.8804	44.4935	53.5069	118.1164	47.424	-42.5701	107.0693	23.3504
-68.4994	115.1243	46.794	53.8619	128.6221	48.2757	-56.3596	122.9931	25.9302
-33.4684	126.8542	39.2439	70.1415	119.673	39.7281	-26.6806	127.8265	22.9897
-43.3447	127.1004	43.2551	71.1118	123.4858	41.7443	-32.7379	129.9429	21.9689
-53.3675	113.0918	39.6037	50.8465	118.5196	43.2635	-46.0552	115.7801	20.1056
-45.8781	122.0435	40.695	60.1865	121.6441	40.8997	-40.1387	125.5305	21.9061
-46.2108	121.9634	40.8531	67.1082	116.7088	40.8303	-36.7606	128.549	21.6688
-47.0716	119.9954	41.225	61.0303	119.9429	40.6883	-38.0538	123.6145	21.4137
-65.2624	133.673	46.7846	60.0791	147.6583	51.2625	-55.6408	140.6104	25.9328
-34.6586	138.1863	52.7267	82.7062	125.2439	39.0386	-25.3321	138.5297	30.1932
-55.0907	141.2096	51.8488	67.1972	144.1815	46.6652	-47.4818	148.0757	26.7021
-41.6326	109.1523	35.2763	57.0162	109.6421	41.3163	-30.8504	120.3604	19.5663
-44.2244	90.8327	35.8645	50.2101	98.3357	44.1022	-34.5298	91.9826	18.1264
-43.2352	100.6686	36.7637	56.7784	106.3494	36.4279	-37.1181	109.8494	18.2702
-44.3026	93.8474	42.4195	63.2913	97.0372	37.15	-37.6427	99.4438	22.3007
-42.6645	103.4675	40.9273	68.84	91.8383	39.6427	-32.7388	105.104	20.9092
-58.8557	136.0256	45.7713	58.3649	144.8291	48.6953	-52.5336	138.8759	24.6433
-51.0425	142.4819	43.356	70.8618	138.6003	44.1059	-43.2616	140.4084	18.2949
-57.8633	140.9212	41.452	61.3681	142.5208	44.2849	-49.9108	141.0551	18.2847
-60.0742	120.5946	40.2383	51.3416	134.2326	40.9312	-55.1497	128.1075	21.6641
-33.3413	145.6724	45.0627	83.3094	134.0385	43.7341	-28.3495	152.2498	24.4742
-32.6165	135.7794	38.8549	84.7358	119.6884	43.9915	-26.9334	138.4833	17.3481
-54.3535	147.7642	46.4024	69.1578	149.5979	60.5544	-44.8096	155.1314	23.7274
-59.5237	136.8759	40.6298	62.5609	141.5741	56.5537	-49.6564	143.1656	23.1925
-49.6207	142.7592	53.4675	76.1578	143.8527	56.5393	-44.3399	149.2838	27.3361
-37.5508	112.669	42.5161	65.7243	109.398	34.8532	-30.4471	116.6736	19.4267
-36.1702	121.9048	39.6568	61.4725	123.3282	36.3082	-30.9164	126.235	20.2142
-42.2186	130.6616	33.7534	69.5727	126.6659	42.1748	-34.8946	136.21	15.5992
-19.4056	96.6843	33.6096	71.0077	82.8559	26.3456	-11.4671	104.4536	16.7949
-38.2292	120.94	40.4033	73.5658	113.1327	38.5499	-30.9243	134.7214	21.4168
-61.5337	105.9566	45.8105	40.9108	127.2343	42.5579	-58.8403	118.0496	26.6792
-60.9958	126.4084	42.6132	50.2012	142.1089	40.8498	-58.4637	135.9377	22.0672
-45.8046	98.0333	23.4314	67.9231	100.5609	26.34	-37.5454	108.9551	5.1541
-56.1271	99.2343	21.5851	61.7249	107.5347	22.2018	-51.5079	110.014	1.153
-46.0061	105.4652	40.535	53.4005	114.5173	48.2477	-40.1392	115.9925	17.2365
-42.2213	90.5515	28.5264	54.8389	94.2329	33.2924	-33.8737	98.0879	11.0754
-48.6165	96.3467	27.1474	51.8227	105.887	30.5142	-44.3671	100.3895	13.0234
-38.9079	102.9621	27.1128	51.9274	102.7668	29.4102	-30.5789	106.6898	12.8296
-30.021	84.1623	24.5636	52.8491	81.8545	26.2788	-22.8401	87.1271	8.1366
-52.1723	96.5438	28.0484	48.8657	107.3563	31.6248	-45.9965	106.1526	13.6749
-37.6122	88.3363	27.51	47.1508	89.8591	31.3332	-29.7887	88.071	10.5496
-40.1955	60.4402	14.3152	46.5016	66.2869	17.9517	-32.8997	67.4441	-1.7498
-57.5138	128.4252	40.793	62.0595	132.1641	52.2605	-46.8168	134.8378	20.1734

-60.4566	130.046	45.474	53.7184	143.0539	45.8464	-54.6809	134.0684	25.7853
-69.1386	137.1197	47.8337	50.2934	156.2489	44.6056	-63.0873	140.6595	23.1818
-64.4042	125.4473	36.4552	47.2478	139.3178	46.5532	-56.9795	124.5724	18.8663
-39.8117	150.1692	44.5472	81.1174	138.5024	52.1141	-31.4618	154.4103	22.9627
-58.7425	93.7542	11.6336	57.9147	103.2658	31.0415	-49.0374	103.5971	-5.6352
-2.5271	149.5045	43.2133	108.9771	115.2727	40.6768	4.0615	155.1159	26.6631
-20.3106	140.018	50.7271	102.3474	115.3972	47.3017	-15.9131	141.1465	30.1102
-71.2112	124.1586	37.0327	47.2191	143.2047	45.917	-65.1387	131.483	16.7221
-15.6999	160.8987	60.562	113.8752	131.5681	55.1078	-10.1079	167.4321	39.7252
-17.3696	144.2232	45.3374	103.2204	113.7254	47.6073	-11.91	142.8903	27.31
-51.663	131.9983	45.7183	65.9894	135.2765	50.5692	-47.6696	136.7128	26.1438
-47.2864	149.7208	49.7372	72.784	149.3574	50.0399	-40.798	149.8883	28.9356
-40.3783	136.3861	48.2059	75.7747	132.0586	47.8779	-33.8604	142.0664	27.7348
-33.8564	137.6783	45.4918	86.28	128.4838	49.7277	-26.0744	147.7467	26.446
-43.6248	132.1436	49.8495	74.9866	127.9266	48.4347	-36.1089	138.5844	26.3918
-30.2563	150.3179	45.5816	92.0955	136.3869	45.3166	-24.7071	155.8353	24.1744
-43.4437	139.121	53.9806	77.3087	134.8807	47.6207	-40.8424	143.4699	28.5952
-42.1626	139.7676	47.712	82.9192	131.5249	43.6701	-36.8281	147.4136	29.0157
-38.3422	108.7746	36.2487	65.11	110.7101	31.5938	-29.0782	110.3522	18.4627
-33.7198	132.7734	39.8278	79.6559	122.8564	40.5998	-29.061	140.9718	21.2512
-44.9234	129.837	40.3549	72.0023	129.1616	36.907	-42.5465	130.06	23.2662
-64.8913	128.3835	37.8455	48.86	145.7446	42.2586	-61.5431	139.0305	22.4717

x25	y25	z25	x26	y26	z26	x27	y27	z27
29.6655	127.5789	28.5208	-56.3377	129.5303	21.6101	26.4317	133.578	36.1829
26.2191	127.5808	17.3059	-62.2552	123.3759	32.4746	24.9626	138.4149	23.7623
59.0753	116.2502	27.1665	-22.3649	132.1001	29.5398	60.2051	122.184	34.1554
31.7793	140.7534	25.4371	-55.765	143.6178	29.5626	31.3451	150.1841	32.6241
54.7168	112.1472	23.7443	-28.2435	135.4881	34.8782	56.0458	126.6791	31.5724
60.2752	124.0876	21.2964	-29.9394	135.9441	33.6425	58.3043	139.5617	31.0824
62.141	144.9864	22.1599	-44.881	152.3598	36.7745	55.9343	156.6553	32.6984
19.1853	114.8755	21.9709	-62.513	103.3755	29.833	18.3541	125.0056	32.4074
56.457	134.3965	32.4681	-38.9859	150.7597	37.8989	53.701	146.28	42.5839
69.6996	147.7325	30.5082	-34.4248	163.297	37.3485	67.6838	159.8969	44.0628
43.7834	116.7221	25.0138	-41.3802	127.5946	34.6031	40.7557	132.7826	35.6595
42.1095	133.6984	26.4198	-59.255	135.663	35.7994	39.5391	148.1879	38.134
64.1699	121.9122	23.6649	-24.2669	143.4987	33.4216	64.3218	136.5923	33.9496
60.8523	128.562	20.1229	-31.5776	145.2641	31.6731	60.7237	142.7547	30.6176
42.1682	122.2486	23.0005	-45.6899	127.2336	30.8062	39.7138	133.0639	33.4088
54.5121	125.8899	21.3158	-39.0592	134.0025	31.77	51.7355	135.3434	31.4355
58.4908	124.8107	18.9502	-33.6486	136.2829	30.2915	57.7353	131.8398	27.6022
53.3422	125.0005	21.5525	-38.2877	129.5582	31.0782	54.3318	129.5337	31.1423
47.1942	152.6184	27.9192	-57.2265	158.2551	37.0671	46.2475	171.1364	38.0467
71.8958	127.3363	20.417	-20.0458	159.5183	42.49	74.0446	148.6219	31.2774
58.7785	157.1011	26.3275	-46.922	162.5382	39.8418	60.0912	164.4956	37.5997
48.3456	120.2022	22.1058	-30.8908	127.2292	28.4259	48.0354	127.9995	31.202
45.6381	101.6914	25.6705	-36.2623	105.7695	29.05	43.3181	111.3704	36.6846
46.536	115.1453	18.9291	-36.5052	119.6488	30.5933	44.3259	125.404	29.1667
52.4272	104.715	19.1304	-35.2573	113.6939	36.0033	50.1811	116.7569	32.5667
61.4284	99.9288	22.1544	-31.5787	116.1841	31.7038	59.3778	109.9123	34.3128
52.4606	147.7494	26.4337	-50.0428	154.0463	36.7091	48.7292	159.1039	39.4892
63.005	139.0445	20.3893	-39.6147	156.8276	35.516	61.449	159.2372	35.9695
56.8316	146.6159	21.5527	-47.3093	156.9911	35.9767	52.6354	162.2942	36.1639
46.4176	140.1037	21.723	-55.9848	139.7426	34.0812	40.9913	152.9123	33.9323
78.8644	140.6941	23.9747	-24.8296	165.0862	36.4503	80.2025	154.1233	36.662
81.4861	125.5071	23.198	-22.3558	154.4081	28.1377	80.1195	145.7098	36.2055
64.0936	156.1526	33.6897	-40.8587	170.9019	31.7172	57.2625	172.0555	40.9437
55.6495	149.5122	35.4849	-46.3674	165.5096	32.7378	47.8028	171.8688	45.2385
68.4584	154.3378	26.0527	-39.7952	163.937	41.0864	64.0774	166.8465	39.4739
58.113	113.9529	16.389	-27.3931	133.537	31.8563	57.2608	130.2981	30.1463
55.3376	127.2246	18.684	-29.8469	136.9232	32.8521	55.0122	141.2937	30.2388
62.9165	138.9152	22.9939	-33.2034	145.7745	27.535	60.3238	145.7302	33.7193
63.092	91.3619	10.4527	-10.1562	111.8661	26.3551	65.5104	99.5952	19.8524
66.4343	129.2594	19.8269	-27.9571	142.7664	32.695	63.9438	138.296	29.6928
31.5335	136.9118	21.9234	-59.5747	128.3123	34.2723	28.4641	145.4664	30.0163
41.5875	148.7869	20.6481	-57.4143	152.2773	37.8685	40.7349	161.9205	35.5669
61.0828	110.414	7.339	-32.8456	133.278	24.6833	53.4829	129.3012	24.0793
52.229	120.022	2.3675	-50.737	123.0023	18.9745	49.0147	130.9029	20.1769
48.5286	126.8774	23.3416	-40.7156	122.5171	27.8781	42.5858	134.9996	33.7955
48.4503	100.6623	16.2255	-35.4723	111.0432	21.9313	48.228	108.0075	26.5224
46.2545	111.8856	15.4124	-43.1318	110.5454	21.982	41.697	121.0655	24.1846
47.9146	104.7578	13.7463	-30.9804	120.4536	23.8518	46.6254	118.5947	24.5734
46.0728	84.5056	10.4997	-20.1856	110.8625	19.2357	44.2802	108.3943	22.3707
42.8566	113.4381	17.0937	-47.0464	110.7852	22.746	40.8278	118.4163	26.3594
42.8154	87.2673	14.2916	-31.1554	104.7127	20.3886	42.2068	105.0037	25.2246
38.6994	71.8475	-0.058	-33.0825	76.4683	10.7591	36.8688	79.3507	11.9122
56.7559	141.4474	30.3711	-45.342	150.8559	30.8901	51.8357	155.5243	40.7083

45.2961	150.1024	23.2986	-53.7848	152.1544	34.1308	40.6752	163.7506	32.7411
44.0181	161.8737	20.7052	-64.6563	166.9854	39.5705	36.0162	182.93	34.9876
40.9658	142.0618	26.9681	-58.0223	147.4708	28.2605	36.9301	157.384	37.1731
73.9596	145.2175	26.6075	-26.0788	172.9883	33.7262	72.5034	166.5753	39.6951
51.1533	110.8885	10.3302	-48.9125	122.7011	10.9735	43.2059	128.6901	26.7002
103.5655	125.1182	23.0065	12.4803	169.3276	37.5683	106.8134	139.2637	34.054
94.5265	120.7548	26.5766	-7.6132	160.1871	41.7974	96.6326	137.9557	37.6419
43.7269	146.7561	26.5199	-65.1404	149.6624	29.4618	37.9778	161.5712	37.2615
109.6683	143.0112	31.424	-0.9651	188.9811	50.1742	111.5633	164.4131	44.4877
100.2706	120.4002	28.7106	-0.3359	160.9944	37.2325	95.724	140.6848	38.4723
58.8063	144.3597	28.9938	-42.5382	154.7532	39.9187	53.9815	160.6433	42.3137
67.2423	154.2377	27.1796	-35.0386	175.8893	39.9161	60.6348	173.3389	39.6784
69.9466	137.7736	27.9028	-30.9524	153.4831	41.2069	69.0008	149.0418	41.057
77.9465	139.3486	24.3901	-24.6902	157.3326	39.5587	76.6745	149.7169	37.1977
68.0133	137.072	26.9557	-34.7378	147.8516	38.8705	68.7511	140.9774	39.4767
86.4128	146.4342	23.5927	-19.6251	170.4947	38.053	84.5872	159.1554	35.751
69.8009	140.3896	22.5222	-36.9375	159.5075	41.5144	69.1742	157.5304	36.0539
76.8128	138.3831	24.0582	-32.2066	158.319	43.6408	75.7534	148.8896	38.8128
55.4508	114.294	14.5214	-27.5761	125.6244	30.4992	54.3961	126.4671	25.9306
76.1908	132.9585	19.658	-24.0714	152.7821	35.9833	72.4553	145.8925	33.0655
68.1105	133.3388	20.378	-37.6746	149.3067	35.6183	61.4582	154.8035	31.8868
44.4311	152.339	24.286	-59.1694	146.5316	35.9196	39.3291	158.5122	37.1365

x28	y28	z28	x29	y29	z29	x30	y30	z30
-48.4788	155.1922	19.0474	12.8437	160.0554	30.0207	-22.2369	138.646	67.2296
-56.6269	154.0233	25.7334	6.9972	164.6639	20.2823	-16.0079	138.1583	70.7569
-8.5772	161.8252	21.7713	53.3487	152.3669	26.1356	17.9513	132.9869	75.048
-48.3804	173.6221	25.1513	17.1248	177.2805	26.9384	-13.0622	152.4689	74.5871
-14.2814	167.0499	26.2097	47.3574	157.6193	24.3665	16.1842	137.9883	74.519
-18.291	168.3608	29.372	50.156	167.5001	26.5019	16.505	151.0937	74.9241
-33.252	185.7028	31.332	42.9013	186.4807	28.656	6.999	169.0169	77.229
-60.1423	133.8075	28.6042	1.9294	150.5269	30.6331	-25.3699	123.1294	74.0978
-29.0211	182.1519	35.1625	45.4133	178.5181	38.309	4.7148	165.4039	83.9925
-21.7877	200.2222	34.0981	61.082	198.1237	39.9117	17.6976	183.2603	86.6326
-33.2355	157.07	29.9769	29.6392	160.75	29.9212	-0.1551	144.1208	77.2706
-55.2623	172.8176	29.6073	29.2814	178.1261	32.9697	-13.6828	158.5825	80.6602
-13.1997	169.7502	26.2607	57.4701	164.8124	27.4601	20.5572	150.4687	76.3393
-20.2928	177.6729	27.0664	54.6888	173.5936	25.4397	15.4954	154.409	77.6649
-38.0619	156.6038	22.8296	29.2551	160.9262	25.6599	-5.4191	144.3726	71.3369
-27.965	164.9686	27.9811	42.0674	164.9617	27.7648	7.8146	151.6259	75.2547
-20.7549	170.592	25.3134	47.003	168.3959	24.9602	13.6817	151.9626	73.2382
-25.424	164.7166	27.3157	42.6672	164.3647	26.9999	8.2058	149.7023	72.1417
-53.4502	193.2233	32.9286	34.0039	206.1114	36.3144	-7.9147	181.4433	86.5904
-10.5131	187.3002	36.8646	67.6075	177.3624	29.4078	30.8885	165.7611	81.2752
-40.5916	200.4976	36.744	39.905	211.297	38.3409	3.8437	201.818	75.7498
-27.9342	138.7381	24.7544	45.3467	139.0789	28.3364	4.5339	144.3761	67.0234
-30.992	131.0416	19.3911	35.0162	136.4007	27.4371	-1.4701	128.9131	65.5245
-35.41	137.5302	26.9613	40.0123	140.1249	25.7531	4.9016	137.5325	66.4353
-31.9654	134.6067	29.2944	47.3656	135.7956	25.8464	7.5121	144.7601	56.5695
-25.486	132.6234	28.2165	57.304	125.8122	29.9549	15.8311	151.8035	52.9583
-38.1015	192.9399	36.7907	32.7075	194.4854	37.2317	-4.22	177.715	78.8325
-28.3691	190.1835	30.54	49.4954	192.9081	31.419	10.0388	176.1718	75.6761
-38.3364	189.3247	30.7318	38.8479	194.4079	31.7813	-0.0679	176.3348	74.7106
-48.4096	172.9594	29.1919	25.3369	182.1645	29.2134	-11.0762	164.0424	71.2634
-9.1015	198.6855	31.5058	71.9387	188.2434	32.6019	28.8514	180.7099	73.7745
-4.676	184.4674	24.6755	74.845	174.6943	30.2679	28.3184	161.4581	71.4978
-31.5757	205.068	26.9174	48.5561	205.9193	34.9639	3.6112	187.9108	79.3049
-39.2286	191.4003	30.4654	39.4171	199.8059	40.055	-5.9633	179.696	77.4752
-29.7156	201.5672	39.6363	52.7161	202.995	36.7002	9.8811	181.1905	82.1243
-19.5915	161.4293	27.7998	49.3471	158.2083	26.892	15.6875	142.8725	68.4546
-21.0102	171.1685	24.1651	44.6453	171.4522	23.4662	13.5867	154.4279	67.3769
-25.9167	173.3012	23.5155	54.5218	169.0416	28.0084	13.0382	164.4182	70.4575
-2.1385	133.261	23.5685	65.3185	118.4743	18.4138	31.5297	119.3903	58.9881
-23.1841	159.0256	28.0768	61.3175	156.5891	26.2291	19.4577	162.7812	65.2074
-55.9603	151.9734	29.0418	16.2877	164.4079	25.2592	-15.0898	144.8436	75.3806
-57.0665	181.4618	30.9809	31.6153	191.0946	29.7531	-13.2896	194.2564	62.893
-29.9316	150.455	22.9258	43.6675	157.5531	24.3692	9.1512	125.6441	64.4011
-41.8773	162.0072	27.0204	36.111	163.5001	26.8108	-1.1928	133.0101	64.1959
-40.0408	136.7924	23.1676	40.3736	148.697	29.6	-2.9007	141.6351	73.6111
-31.3562	132.7079	19.5232	40.5506	133.6013	24.8434	2.003	121.4558	61.645
-37.6292	136.1904	18.5985	33.4847	143.2166	20.537	-2.2853	126.5365	58.551
-27.2814	135.7209	24.249	42.6018	136.1787	25.2502	5.7749	126.8692	60.3154
-18.0754	121.3196	18.2149	42.1636	118.6292	21.3021	9.2126	109.4368	56.1578
-44.4211	125.4713	20.9826	36.1421	130.88	24.3656	-4.5337	128.5322	59.8083
-24.4402	123.4527	16.8812	33.8651	123.0905	22.7384	4.1196	114.8945	57.9689
-26.4348	96.1639	15.0214	26.1656	100.7553	16.112	-0.3054	74.0685	46.2564
-36.1131	183.7695	25.9576	43.3215	186.2034	34.9095	-2.055	167.7714	78.3691

-47.9021	182.843	27.6374	28.2394	192.6482	24.3545	-8.3693	171.1138	71.3571
-62.1151	196.9502	30.3518	21.9933	214.0856	27.7209	-13.5533	186.0486	80.703
-49.6919	175.9233	25.1475	23.9435	185.1719	31.517	-15.9753	161.9387	75.915
-9.9079	206.2553	26.8029	67.6946	198.0455	31.0413	21.7891	185.2361	81.0747
-45.3929	148.7849	11.7655	35.8117	152.9234	25.9408	-10.612	128.7069	57.5412
31.1335	198.4459	28.8155	107.425	174.296	27.3403	63.0243	168.0471	73.9259
11.8283	188.9498	37.9655	89.9659	170.6693	35.9721	48.6089	164.4831	80.454
-60.2268	186.1953	31.0659	17.8932	198.2409	37.1561	-21.1005	167.4876	74.1677
14.9187	216.9769	42.0932	110.2269	192.19	38.2947	60.0188	187.467	93.0988
15.8902	185.9261	30.3543	92.7762	167.5353	32.285	50.823	167.3493	76.6442
-32.7642	188.6487	33.0744	41.0211	190.7246	35.9345	3.6383	164.8222	82.8968
-22.0124	206.807	34.8399	52.6129	202.9882	36.1912	14.9435	185.4154	81.4516
-17.9085	185.9417	33.8836	59.5773	177.2185	34.9539	18.8999	170.3936	78.461
-19.3825	170.2032	34.3824	72.7817	161.8678	33.0693	29.7835	168.996	80.2122
-15.9465	194.319	33.7665	55.7666	187.874	35.1517	15.4796	166.7356	77.513
-6.5344	204.6681	33.7074	78.3937	197.0673	30.8692	34.5891	183.593	80.1211
-25.9252	196.6039	37.2819	60.2565	190.9837	32.9021	18.8837	176.6724	80.7123
-14.5845	195.7377	34.0204	64.7814	185.0937	31.5956	21.449	172.6861	78.28
-26.1337	137.3506	28.5317	52.5993	136.5033	24.297	14.3636	136.2874	60.1596
-16.0919	176.8942	29.0605	68.4925	168.4122	28.5691	23.8218	129.146	81.7316
-32.5701	162.4189	33.3956	58.6554	163.5837	30.406	12.6994	163.8714	68.6927
-54.9712	161.0567	33.5856	33.7867	171.9587	35.6717	-13.5357	171.8699	74.0192

x31	y31	z31	x32	y32	z32	x33	y33	z33
-21.6136	177.7741	24.7153	-42.6649	166.3953	24.847	-0.1713	172.0394	31.781
-29.0172	179.8079	23.3693	-50.8029	165.4963	26.5712	-6.9176	176.3456	24.072
22.84	180.2586	24.9733	1.3086	173.9047	29.3581	43.1325	168.597	30.7295
-19.3293	200.6507	25.9739	-41.4217	187.2718	28.9353	5.862	191.1963	28.9355
16.4021	187.1635	23.5682	-4.5455	177.5719	29.7284	36.0151	173.904	28.0192
14.3203	193.7226	27.1344	-7.4454	182.5571	33.2011	38.7141	182.6039	32.6158
2.6415	215.6461	30.9766	-20.9856	203.2047	36.1209	26.6179	207.5042	35.2237
-34.2453	165.343	28.2851	-54.9899	146.0797	32.0245	-7.4177	158.4532	33.9055
7.4471	213.4115	39.5001	-18.6312	199.0432	38.6035	31.6551	201.001	45.5834
20.9762	236.3242	38.419	-8.4414	222.0287	39.4048	46.3135	221.3218	45.1596
-3.778	183.5997	32.5079	-24.4267	173.3279	34.8065	20.2467	174.1301	33.426
-16.3753	208.9018	33.1662	-42.0376	194.2336	35.8143	12.7499	197.3102	39.2193
24.0131	195.7923	28.9777	-1.6369	184.7307	30.1885	48.0678	181.7238	29.7996
16.7497	204.7343	33.514	-8.4225	190.7943	30.6582	42.2472	188.476	29.9577
-5.8645	184.1983	28.5718	-30.6709	170.3415	27.2774	19.2138	174.2759	30.2329
7.8099	191.4704	28.79	-16.7108	180.1484	31.6696	27.8304	181.5216	36.2181
13.4291	196.1402	29.3272	-10.5359	185.1223	30.5717	39.0246	181.0587	28.7136
8.2341	189.5209	26.6888	-14.7714	179.9681	31.8267	31.8593	179.3	31.463
-13.4557	235.4438	36.109	-40.5025	214.6291	41.1315	18.1141	222.3182	41.7839
34.9156	215.9655	37.6592	2.5263	202.5532	42.2285	57.6231	201.3445	37.3953
2.9542	240.7832	40.2537	-25.3997	229.6416	37.3522	27.7883	233.5841	39.1525
7.817	181.2845	25.7151	-16.0173	173.1627	24.6427	32.3891	172.942	27.7828
1.9093	159.0272	22.944	-21.4292	146.8973	22.3855	22.5612	152.2251	28.7552
-0.9219	176.7614	32.3039	-24.5684	164.2865	29.5592	27.0337	167.56	29.3918
6.359	168.6874	32.3497	-19.9269	157.4442	34.9929	33.5747	161.1302	31.845
16.6024	169.9427	32.3905	-5.9357	168.0813	30.0033	41.1564	166.4398	31.4291
-6.7601	217.9784	35.4146	-29.4943	207.5735	42.2427	19.0376	210.1022	40.9152
9.3053	218.7827	34.6827	-15.6825	208.8412	37.2855	34.0647	210.1667	39.3051
-3.1193	219.9615	32.2215	-27.3924	207.6136	35.7172	24.4302	212.2794	36.4026
-15.5519	201.5543	32.0105	-37.26	190.4399	34.7875	7.2047	198.0663	35.0259
33.28	225.4745	33.1833	6.54	216.537	36.8092	55.9277	211.7359	40.592
40.0311	210.1892	32.9846	11.2034	203.4135	29.8142	64.5113	196.6673	34.7272
9.2799	238.7301	30.6446	-19.2822	226.3439	34.0074	33.9593	227.9906	37.005
0.6499	229.978	35.6997	-26.641	215.368	36.431	28.3427	218.623	43.9243
9.5978	234.3023	34.0489	-17.0404	221.4817	43.7834	37.0898	226.5248	39.577
14.8386	185.06	28.8733	-8.1621	175.776	33.3561	38.1232	175.4189	32.5803
11.4997	194.4273	20.9674	-10.7715	185.4861	28.0407	35.1487	187.1784	24.163
14.7567	206.0222	30.4577	-11.1159	195.3249	30.07	36.7356	198.4002	32.5903
36.9179	154.2757	27.2924	12.5912	149.4888	28.4984	57.1102	143.482	25.9381
21.1318	202.2902	30.8602	-11.5273	190.0105	33.5481	51.2876	186.8889	34.759
-25.5193	183.8898	22.8448	-45.1286	171.1243	28.8607	-0.8024	180.2414	26.5512
-16.6436	215.7785	39.7342	-44.0522	203.6434	39.1525	17.014	210.4186	36.2771
4.552	180.6807	34.4651	-18.717	170.9363	33.3595	33.8456	172.6561	30.5552
-5.8348	186.6549	38.9704	-32.4032	174.6708	33.9232	23.3646	177.2444	34.4861
-5.6501	189.1081	31.4548	-32.1652	175.6043	28.1502	23.8804	183.0282	32.289
4.1126	162.1651	23.557	-17.6231	151.5659	27.5882	23.5943	154.5183	33.0407
-4.6185	164.8376	19.9842	-26.8064	153.0338	21.5878	19.2636	157.8903	21.7894
7.0319	165.4714	23.295	-13.6095	156.0555	26.7612	28.8289	156.2021	29.1636
13.5708	143.8432	25.5999	-9.1556	133.3159	23.5493	34.7957	131.9044	26.066
-6.8147	169.8233	20.3536	-30.0096	156.567	22.0919	18.2811	159.7754	23.9468
4.2549	145.7115	20.9464	-16.2808	135.5092	21.1726	23.8839	137.1578	24.2087
-1.3457	112.8539	23.1301	-18.6781	106.2748	22.4395	15.9162	108.2585	24.2123
3.881	217.7134	35.0873	-22.1493	205.8248	34.9752	29.2597	207.8412	40.0255

-13.011	220.5006	33.5526	-37.0582	206.6895	35.2445	13.4098	213.2187	31.5838
-23.1385	240.2719	33.6912	-49.8613	223.0248	40.7238	7.0948	231.0728	38.8878
-17.5751	213.4713	31.8116	-41.747	196.2853	31.4703	10.6462	204.4384	34.9517
32.2514	236.617	35.1271	2.0539	224.4442	33.3864	58.765	220.2927	37.4168
-10.4495	185.4066	33.7564	-33.0823	170.2596	27.989	16.4714	175.1324	35.3721
76.9465	213.9167	34.1586	47.9344	210.388	35.3683	98.2231	195.0346	34.7072
53.6624	205.4882	37.4685	29.4636	203.0463	42.1174	80.2431	191.2207	39.9177
-26.3239	216.2612	38.0368	-51.5403	201.3605	36.8566	1.7653	210.4082	40.3116
72.0584	244.6282	50.9956	39.5828	238.117	51.2722	95.9814	223.7881	50.0868
62.9085	210.1845	34.0956	35.0904	205.0917	35.5163	85.639	192.5699	36.4614
3.6929	216.3103	34.4119	-24.3302	204.424	34.1047	29.5799	208.1317	36.5336
17.0045	231.1601	34.9111	-10.2824	222.1031	37.6579	43.0865	219.7462	38.7438
21.0593	212.9619	32.7712	-5.0392	204.5349	34.4248	44.379	202.7849	36.4964
32.9808	216.2511	32.1327	5.2887	207.4182	35.727	56.3281	203.0582	33.4478
18.5042	213.9125	31.9412	-1.1279	207.7605	38.5167	39.7557	206.2252	38.7447
39.4032	233.535	36.9104	11.1977	224.9735	40.5429	65.0446	220.3777	37.7353
17.7468	226.9861	36.2232	-10.6374	216.2887	40.9566	44.4759	215.0619	39.1932
25.0214	221.0486	32.852	0.7512	213.076	37.9314	48.4925	210.9894	37.4131
11.6154	178.2146	23.3358	-21.5841	157.1808	24.6552	46.2214	159.6353	21.0955
29.5198	207.3048	32.745	-1.2071	197.2337	34.4717	54.5061	197.0531	35.816
13.1121	208.8366	27.7085	-12.1712	198.7097	34.9213	38.5588	200.2428	32.8947
-17.4758	215.5107	31.0498	-44.5364	197.766	32.5962	10.948	206.8087	35.4506

x34	y34	z34	x35	y35	z35	x36	y36	z36
-15.4154	168.5654	-0.647	5.3889	-25.5158	-5.4101	-0.505	2.1399	0.546
-30.5871	171.1365	-0.3612	10.691	-22.7906	-2.0225	-0.5757	-1.492	-0.0623
21.9911	173.4577	0.0934	6.3937	-20.7451	-0.4169	-0.6727	1.4529	2.6267
-19.0516	193.2365	-0.004	9.0671	-23.9371	-2.7172	0.3111	-1.3841	0.7734
12.6556	178.1541	1.5774	11.7929	-19.4614	-0.7229	2.0723	1.1803	0.2854
14.7878	184.3485	0.0177	6.9812	-28.3451	-3.1906	-0.4772	0.9385	0.6192
2.3046	205.1264	0.103	13.2813	-34.0005	-7.1989	0.2815	-0.6149	-0.3791
-31.7322	157.3477	-0.0068	14.7523	-19.9567	-0.4205	-0.3745	-0.0922	-0.0142
10.2663	201.2146	-0.398	7.4852	-23.1385	-0.5018	0.5495	-0.6573	0.7082
23.4084	226.6519	0.135	12.2946	-36.798	-10.4574	0.2413	0.3284	0.3562
-2.8323	173.0853	-0.1335	7.0487	-25.0364	-4.314	-0.4867	4.0031	0.8853
-14.1315	199.0233	0.5068	8.4821	-26.274	-4.8242	-0.8724	1.8606	0.9289
24.5694	184.6885	0.2571	6.0574	-23.2014	-1.6458	-0.2521	2.4273	0.6103
17.277	194.6012	-0.0332	8.6023	-25.0576	-2.195	0.1343	0.7693	-0.0024
-5.0622	175.4124	-0.0038	8.1404	-25.9653	-3.9364	-0.7197	-0.5906	-0.2943
7.781	182.6832	0.0114	7.7781	-24.1378	-2.1412	-0.1244	0.024	-0.0563
13.8629	185.1992	0.0806	6.1527	-26.4051	0.5814	-0.4994	-0.2099	-0.0547
7.6831	180.1352	0.0221	4.8181	-22.5766	1.0663	-0.4442	0.221	-0.0206
-10.971	226.4368	0.0756	13.4745	-28.3534	-6.5158	0.2513	-0.1003	0.095
23.4677	209.0305	-2.5765	6.129	-20.4399	-2.7785	0.5738	0.1895	-0.0026
1.1818	234.5631	0.2899	11.4285	-22.5137	-2.539	0.082	-0.7211	-0.2358
8.9509	176.6667	-0.1177	10.1002	-21.0416	-3.915	0.1068	-0.8493	-0.0906
5.4823	151.8534	-0.7351	10.8188	-16.4074	-7.2571	0.2431	-1.0618	-0.2293
-0.8796	175.1466	2.2364	6.9137	-21.0033	-9.7453	0.6238	1.1521	0.681
5.8138	168.0689	0.363	12.5031	-23.7538	-16.9787	-0.1588	0.0073	0.1015
19.9821	170.8647	0.6739	10.7523	-20.8589	-11.1514	0.2853	0.6846	0.2368
-6.4812	210.948	-0.0604	13.219	-20.3348	-3.2581	-1.1236	0.0821	0.1645
8.654	216.9081	0.8645	7.6654	-30.3496	-3.3934	0.0594	-1.7365	-0.4795
-1.4888	216.8135	-0.0848	12.3648	-34.8738	-5.7082	0.959	-2.6113	-0.8437
-14.7079	196.8506	-0.7891	11.3002	-27.1648	-3.1534	0.9629	-1.404	-0.7341
34.4149	222.8506	-0.1282	8.2815	-32.3803	-2.7643	-0.0285	2.0895	0.3584
41.2358	205.2482	-0.1043	8.0635	-33.2915	-3.0467	0.094	0.0633	-0.265
13.6691	228.2689	-0.3146	13.6687	-33.2638	-6.1727	0.9201	-0.7984	-0.6792
4.1275	222.8648	-0.5314	8.9064	-37.3907	-7.0765	-0.0068	-0.031	0.243
7.6768	226.8166	0.4662	10.0145	-32.8054	-4.0199	0.0912	0.1334	-0.197
15.4273	183.7568	0.6026	5.1385	-24.9231	-6.3509	0.4605	4.2265	0.9417
10.1887	188.1163	-0.6427	9.1986	-24.2955	-1.6869	0.1755	1.137	-0.6215
16.462	199.2414	-0.2885	12.2505	-30.9658	-3.3896	0.6883	-1.9568	-0.8772
32.9946	151.6153	0.3711	4.065	-24.7797	-4.2128	0.0293	-0.7169	0.089
21.0317	200.7604	-0.6045	9.4985	-25.0662	-4.4781	0.2997	0.031	-0.0905
-24.3056	177.551	0.2455	9.3835	-18.8648	-1.3785	-0.0429	4.2867	2.3494
-17.5469	218.7763	0.0952	10.8545	-31.2313	-7.3151	0.0014	0.0815	-0.1998
6.61	181.6014	-0.2422	17.4941	-50.9179	-57.5825	5.1546	-22.0603	-48.2686
-3.8891	196.1376	0.4819	21.2675	-44.1295	-67.0626	8.852	-16.551	-53.8873
-3.8946	188.3637	0.8738	18.3169	-29.9788	-13.7521	1.9525	-1.2598	-1.7892
5.744	158.6794	0.1607	10.8394	-20.7608	-4.216	1.2492	-1.4173	-1.2621
-3.3734	159.4119	-1.1467	10.1612	-23.1337	-3.9507	0.8762	-1.4854	-1.1613
9.0728	160.4415	0.3182	8.3874	-20.7038	-1.7303	0.277	0.4905	-0.7748
14.3705	140.4284	-0.0073	7.7238	-18.9246	-0.5295	1.0145	-1.0423	-0.1038
-5.9196	164.3334	-0.4097	10.5784	-16.7377	1.1878	0.1707	0.1782	-0.0251
5.6669	140.1096	-0.2313	7.7724	-15.7414	-1.9169	1.1865	-0.4558	-0.0521
-0.8649	115.6383	0.0081	13.2315	-27.4352	-35.7475	3.0033	-13.5538	-33.0258
8.2402	211.0741	-0.1058	7.7231	-27.4957	-2.7113	-0.1801	-0.1605	0.163

-14.25	213.7269	-0.1192	9.6458	-32.284	-5.8797	0.4311	0.2804	-0.4031
-24.0737	239.0726	0.0408	9.9085	-35.6294	-2.0443	-0.4084	0.4595	0.2346
-14.1853	209.2015	0.3921	10.8438	-23.0539	-3.8934	0.4201	-0.0032	0.0615
33.5766	229.6104	-0.1674	11.4788	-35.3459	-4.325	0.0031	-0.3008	-0.0893
-4.6429	194.8252	0.177	9.189	-50.5286	-51.5372	1.0001	-20.2729	-44.7879
77.5504	214.2812	-0.7055	8.9547	-30.6239	-3.7761	-0.4513	0.3294	0.5506
51.9125	201.716	0.7672	7.6313	-28.1703	-3.9179	1.1291	-0.3847	-0.5347
-24.0489	217.0459	0.0804	13.254	-23.0915	-1.9307	1.1768	-0.8415	-0.7422
69.7108	245.4952	1.2336	4.3684	-35.4981	-9.1263	-0.1222	-0.7933	-0.1557
61.9187	200.8575	0.265	5.8785	-34.6926	-7.1026	0.1706	-0.5424	0.9545
4.933	213.171	-0.3158	13.8861	-31.3345	-5.1589	-0.059	-0.6076	0.0685
18.5933	223.331	-0.121	9.0721	-32.0665	-2.5415	-0.0145	0.8009	0.4908
20.5605	209.655	-0.0596	11.3461	-33.4793	-5.231	0.6114	-1.3373	-0.7186
30.9035	209.3589	0.1511	12.1662	-32.7917	-7.5382	1.9247	-0.0177	0.2948
19.6902	209.3807	-0.2142	10.5887	-30.8729	-2.1447	-0.9476	-1.1465	0.3718
36.4037	233.9408	1.5676	10.0793	-31.0658	-0.0757	0.1495	-0.2056	-0.0779
16.2432	224.2986	-0.6608	11.8256	-30.7177	-4.7831	0.7651	-0.8869	-0.5866
23.8171	219.2827	1.4671	10.476	-28.8381	-3.3352	-0.5795	0.577	0.0205
10.4247	172.2789	-0.2422	10.9382	-24.6734	-3.8162	0.5781	0.4348	-0.1983
28.8609	204.2979	0.1819	12.9068	-32.7448	-5.3536	0.9789	0.6877	-0.2295
11.2348	204.6033	-0.0747	13.8984	-27.8137	-1.042	-0.5875	0.3004	0.3164
-16.47	209.861	0.2338	12.1895	-24.5059	0.7848	0.5649	-0.7369	-0.2494

x37	y37	z37	x38	y38	z38	x39	y39	z39
13.0871	1.7641	0.2381	-6.2186	24.7606	-1.1134	18.3047	23.3336	-0.8877
16.494	0.0891	0.2741	-8.8535	26.838	0.0973	17.8679	30.3992	0.4467
14.1797	1.5177	1.0398	-5.2933	31.7078	4.967	23.049	31.1845	0.2883
18.0083	-2.2852	-1.0749	-8.5065	37.0742	0.7866	25.9417	36.4382	-0.9271
17.2524	1.5414	0.7973	-4.9963	32.0182	0.0523	19.1837	34.1344	1.8096
19.1272	-0.169	0.8364	-2.4853	28.2518	-1.0698	24.3751	27.3937	-2.0862
24.7174	0.0742	-0.2578	-6.3128	26.8945	-0.9868	27.2531	28.9553	-2.3673
13.9517	0.6178	0.1123	-9.2419	22.5229	-1.4007	14.2056	26.5265	-0.9979
13.1399	0.5348	0.0889	-6.001	34.0391	4.7414	16.875	35.9065	4.9435
25.4146	-0.5778	-0.0529	-4.1863	35.0839	1.4833	33.4646	30.0773	1.6776
16.6764	1.7607	0.1523	-2.3883	27.5154	-1.5031	23.574	26.4591	-2.1585
18.4652	1.9559	0.9613	-4.3116	31.1733	2.8942	21.4716	30.5182	2.0958
17.829	-0.3199	-0.156	0.2582	34.2521	0.1017	25.6339	30.1684	-0.6686
17.0809	-0.0744	-0.442	-1.6863	32.0222	0.0006	21.1872	31	-0.4112
13.4387	-0.4654	-0.2861	-7.072	26.923	-0.8843	17.5131	27.8963	0.7658
15.2909	0.4004	0.0492	-4.995	28.9193	-1.7679	20.6237	27.4753	-1.0009
13.4427	-0.9458	-0.2468	-4.9233	28.1683	0.027	17.989	28.1779	-0.2651
12.836	0.1458	-0.1723	-4.3903	32.575	0.7201	20.0416	30.6799	0.6712
22.401	0.3378	-0.1337	-10.6563	34.6674	4.2228	24.7909	38.1145	1.9609
14.4128	-0.2981	-0.0812	-4.691	37.796	3.5329	23.5132	36.0936	3.9981
19.5017	0.2648	-0.0098	-11.9478	37.1484	3.2436	23.1514	40.5342	3.2768
16.0149	-0.0848	0.0559	-6.3481	21.8612	-0.553	18.4165	23.6378	-0.7681
19.8426	0.2188	0.6776	-2.3812	16.4198	9.6663	20.9127	16.741	7.8987
19.8879	-0.0124	0.0064	-1.3738	18.6616	5.2467	25.9274	18.7076	5.3225
23.7403	0.1751	-0.3069	-1.6598	10.3642	4.2323	25.1893	11.8873	3.9639
21.8265	-0.6148	-0.0706	0.4922	18.2002	4.8696	26.0762	15.0192	6.5406
20.2711	1.8441	-0.4798	-15.8915	30.7758	-5.8423	18.7752	37.94	-5.7995
20.5982	-1.4084	0.1899	-6.4357	38.1007	-4.3004	31.0873	37.271	-2.6578
23.457	0.2998	0.673	-7.9994	31.7757	-4.7853	28.8548	33.8803	-5.4605
20.8544	-0.9229	-0.3015	-7.8216	31.008	-3.5297	24.4959	32.8096	-5.9864
21.0941	2.204	-0.1733	-10.9662	42.1339	-2.2442	27.5661	34.5052	-6.6181
19.2995	-0.5852	-0.1188	-9.7109	30.6205	-6.8593	30.1096	29.7431	-7.6783
22.4133	-0.2474	-0.0127	-12.4513	36.3805	-4.345	34.0827	37.1185	-3.4689
18.0074	-0.2567	0.1298	-10.551	37.8629	-6.2871	29.826	34.1191	-4.5946
21.7375	0.0242	0.0505	-12.0467	34.804	-9.9134	36.9459	33.3868	-8.6788
19.221	2.1901	-0.5014	-2.2931	29.0055	-3.2629	28.4415	21.1139	-5.4762
19.2	-0.8206	-0.1462	-6.0593	31.4678	-1.112	26.5883	30.082	-3.6919
23.2652	-0.4915	0.0753	-6.8615	30.2938	-4.4026	30.6363	29.5023	-1.9185
19.7491	-0.7703	-0.3384	1.919	20.6753	-1.7046	24.3381	15.0988	-5.0241
19.879	-0.1031	-0.301	-7.2887	30.386	0.3853	27.2524	30.8855	-2.3174
18.6561	5.1237	2.9433	-11.2738	35.1296	-2.7752	26.9398	36.1292	-2.3201
23.4124	-0.4848	-0.5891	-7.9525	30.7879	-0.9754	30.6496	30.7306	0.9734
26.9432	-25.1533	-48.7709	-0.5172	11.8418	-43.9209	32.2518	11.9014	-42.1877
30.491	-13.929	-52.727	1.3133	16.63	-50.679	34.1429	17.147	-49.555
27.5815	-0.6081	-0.2275	-3.5784	15.2192	-4.1026	31.8561	16.9034	0.1559
19.0444	-0.8337	-1.6699	-5.5832	18.1069	-5.7738	22.9907	19.5971	-3.7231
22.1675	0.0719	-0.7023	-2.859	27.4222	-2.3492	27.5744	24.7348	-4.2174
17.4449	0.1904	0.4875	-4.1529	26.9359	-3.1722	21.2863	26.9177	-3.3417
16.0514	-0.8827	-0.3921	-2.8157	20.8692	-2.7328	20.4955	20.281	-1.6731
18.9422	1.2188	0.1106	-10.3754	33.8543	-3.4182	24.347	36.2407	-3.9705
13.163	-0.7666	-0.4035	-4.5991	20.124	0.4623	19.9351	20.3226	-0.7869
19.3684	-11.7797	-31.6176	-2.0265	6.6527	-31.8671	20.3907	6.5588	-32.285
19.1604	-0.4524	-0.033	-8.4572	42.9899	-5.1692	37.3768	38.4784	-7.8601

20.8393	-0.9181	0.007	-7.7842	32.8179	-6.6603	32.2022	31.734	-6.6639
18.8634	0.0653	-0.0919	-16.5882	37.3907	-10.4353	34.628	38.7815	-8.9566
19.3722	0.5596	0.0772	-10.1809	38.8988	-2.6573	28.23	40.2848	-3.5976
23.3984	-0.0343	0.0125	-9.0789	40.0151	-4.0339	33.9537	37.1378	-2.3675
20.4125	-20.8336	-44.7789	-7.7084	15.5359	-45.8927	29.0828	16.1896	-45.896
20.507	1.2957	0.0908	-8.7589	37.1479	-2.539	27.9984	37.1063	-4.9444
21.9753	0.0315	-0.0854	-6.2128	39.1282	-2.9678	38.5177	32.1284	-4.0965
19.2118	1.0058	-0.4298	-14.4989	40.1975	-7.3631	20.6322	45.7184	-7.0436
25.6313	-1.1837	-0.569	2.0145	43.8915	-2.3226	41.918	34.8355	-5.1766
21.0945	-0.0681	0.7265	-7.6329	34.0013	-1.9005	33.5103	29.6147	-4.4786
23.4299	0.6632	0.1441	-6.8003	33.0357	-4.7964	27.4294	34.6553	-4.2048
22.4008	-0.259	0.1294	-6.6668	39.5407	-3.9304	33.9661	35.8594	-2.0883
21.2409	-0.5318	-0.5613	-8.6949	33.4329	-0.0031	30.1828	33.7541	-0.4693
23.5079	0.0761	0.486	-4.5217	32.9328	-2.5691	29.6014	32.705	-2.6919
18.0168	-0.2832	-0.0029	-7.5552	32.206	-2.8475	23.1726	32.5017	-3.3568
20.2938	0.8386	-0.1008	-8.5527	37.4248	-4.6511	30.1507	38.2138	-3.4509
20.5583	0.3723	0.7242	-11.0214	30.5373	-5.0626	27.3972	31.2917	-4.3097
17.3366	2.4195	0.1499	-16.6882	33.2712	-4.7844	26.7355	38.8578	-7.6169
22.874	-0.262	0.2816	-3.6788	28.3504	-2.804	30.2816	26.9644	-2.3645
27.6707	0.5043	0.2095	-1.7305	31.6464	-3.6639	36.4808	28.7595	-1.6527
22.6998	-0.1359	-0.4967	-10.0194	34.495	2.2636	28.0308	37.6068	-4.7655
20.5985	-0.8534	-0.0391	-13.3091	38.5479	-6.3019	29.5923	41.68	-1.4236

x40	y40	z40	x41	y41	z41	x42	y42	z42
-16.9711	91.6318	-0.2434	32.0137	91.9279	-0.2889	5.9133	6.2112	-0.7796
-30.6989	97.5863	4.9278	16.1798	106.0444	6.9625	5.0501	11.0061	1.6831
-10.7666	104.9375	9.0779	33.7853	103.586	3.8892	8.2589	15.7137	3.7216
-19.5961	108.6554	1.0136	35.7768	107.1466	-3.048	6.8994	14.372	0.4811
-29.5681	97.9672	2.2554	16.2878	103.3025	8.434	6.1552	12.042	3.3547
-10.2882	103.1022	5.88	40.2917	101.6501	3.497	10.2631	10.1637	0.7095
-26.3765	98.8937	0.2768	32.1779	104.0148	-3.1493	11.102	5.1111	-0.9472
-31.709	78.4756	3.4381	12.7782	88.8761	3.4024	5.1856	8.772	0.829
-19.045	109.1819	10.0662	35.0312	109.3579	8.6102	5.3308	19.3723	2.5331
-7.2083	114.8206	2.3157	50.8767	108.3906	-0.1281	12.7229	15.2448	2.194
-6.2574	89.4128	7.2609	36.7464	87.3446	6.6535	9.3335	7.6001	0.6957
-20.3443	101.9936	5.0156	35.9761	103.1727	2.7208	8.4358	11.1093	2.455
-2.2407	105.1253	4.2898	46.9379	100.505	1.2591	9.6744	11.1003	0.7702
-15.7929	101.831	-0.6341	36.1786	102.6243	-1.5434	9.144	11.14	0.9164
-23.0926	93.7534	0.7639	25.5208	98.0581	3.5562	6.135	12.8207	1.1422
-14.2149	100.6834	3.8382	31.2387	101.4049	2.6151	7.4513	15.8331	1.398
-17.4574	99.6156	-1.1051	33.2325	101.3509	-2.7304	6.4852	9.405	0.5843
-13.4329	99.3103	4.0538	37.5977	98.7741	2.1598	6.5652	15.1881	1.3612
-29.2823	113.0968	5.5716	28.4706	119.4115	3.7792	8.4189	21.4393	3.0854
-12.2054	107.9562	4.4257	41.634	105.8424	1.7278	8.6896	16.6129	6.3168
-28.7564	110.104	1.8129	25.9746	115.9952	3.5394	7.7954	17.8226	3.816
-23.9758	86.2201	-5.0837	27.4842	90.0285	-3.9543	8.4016	6.9764	3.0646
-14.4423	75.95	6.3424	37.9176	77.3162	5.9755	10.533	10.8237	5.6561
-12.2106	88.1855	4.5288	42.1038	78.0949	1.1449	10.1461	7.1595	4.9711
-18.1614	68.8823	-0.5308	40.5448	70.7274	-2.7925	11.912	5.303	3.9999
-7.1907	84.5584	6.8286	48.1891	77.3165	12.1046	11.2816	9.3642	7.3999
-42.7988	100.4173	-1.5233	16.7182	113.5793	-2.4287	7.2869	10.4663	-0.9623
-10.8861	110.6266	-4.9745	45.5908	107.5631	-1.2551	12.4875	16.1323	3.5132
-19.922	102.0937	-5.5832	39.4237	105.3241	-3.1862	12.0218	9.1294	0.5956
-19.5198	104.2748	5.3866	38.4171	103.6308	4.0536	9.6347	9.374	0.56
-11.8824	111.2352	-2.9814	46.6582	109.1575	-5.2683	10.3638	5.0083	0.3139
-22.5861	102.0731	-2.9923	47.1173	97.7419	-5.1592	9.3574	2.9417	-0.175
-23.6295	108.4724	-7.0774	38.1778	110.6131	-2.8875	11.5325	6.9	1.0632
-19.7587	112.793	-3.2373	41.36	112.5163	-1.3338	9.3392	5.707	2.9377
-22.3598	103.7938	-8.6839	48.3802	101.0458	-7.3086	11.129	4.9359	2.4693
14.6077	98.4955	6.9589	54.541	88.9305	1.8841	10.1288	4.4616	1.3303
-9.7082	99.2346	-0.9946	41.5551	99.0202	-3.9564	10.8006	12.0999	-0.1317
-17.0314	102.1065	-6.6911	43.8123	100.0095	-4.562	12.4984	9.0865	0.2817
7.8824	76.4091	5.1151	41.3808	69.0559	-1.4181	10.795	3.0742	-0.1219
-19.6403	102.7147	2.4125	35.3018	104.4879	-0.8489	10.0444	10.1286	2.6862
-24.4557	93.5761	-1.8187	33.4293	96.2167	-3.9091	8.5591	11.4995	2.2623
-19.9095	109.1795	-1.5005	40.7143	110.4548	-1.4695	12.0749	11.1349	4.6523
-11.9139	81.8325	-30.1617	43.434	82.6755	-25.8177	16.0492	-14.8707	-45.7379
-16.3178	90.1555	-28.9995	40.5796	92.9115	-26.6925	18.6771	-5.454	-50.7268
-15.072	88.1748	5.8961	33.0217	91.9121	6.2899	14.4008	-2.354	-1.5153
-14.2528	75.2283	-0.2867	29.6333	76.1423	-1.1935	10.0084	-2.2142	-0.4672
-11.1827	82.032	-3.9918	43.2148	81.0418	-6.7017	11.8979	3.928	-1.211
-13.8225	80.3304	-4.0198	30.5165	80.1943	-5.803	8.1914	9.7651	1.4227
-9.0362	66.1048	-0.9595	31.331	66.5075	-1.0588	9.6148	6.5972	2.5115
-25.6254	82.9289	-5.8094	31.1734	86.653	-4.973	8.0382	10.3491	0.8895
-10.6656	71.4805	5.5546	27.2102	71.4954	3.5172	8.0152	7.0287	1.8852
-15.9738	54.9111	-13.7284	24.7992	58.8491	-14.7331	9.6279	-3.7278	-30.1879
-9.3108	111.1668	-3.8039	50.5966	104.7822	-3.6044	10.1271	10.352	1.0978

-15.9291	107.9056	-2.8569	44.227	106.0787	-4.2234	10.6444	4.6664	0.7937
-22.1983	107.1852	-15.7585	40.4703	106.5742	-16.4448	9.4727	8.0965	1.2965
-23.7267	109.6182	-1.14	37.1828	111.1994	-2.0329	9.1731	16.8412	4.2605
-17.1716	112.9105	-8.3639	45.2402	111.0442	-6.4544	11.7907	6.5434	3.8797
-18.8176	84.8962	-28.2311	39.7365	85.8587	-30.2264	10.7911	-15.5163	-45.6875
-17.3395	110.2915	0.7416	39.7237	109.3856	-2.1577	9.5515	7.1257	1.0601
-4.3137	105.5579	2.6476	56.531	97.5541	-1.7906	11.9022	9.5687	1.7997
-36.9075	112.3219	-6.3051	18.9166	120.3943	-3.5676	7.0197	18.6425	-0.8274
5.3158	117.1198	-4.8867	71.2682	102.5703	-8.466	15.4014	8.6158	-0.9618
-12.3506	105.9682	1.1385	56.3385	98.3046	-3.0395	11.5929	2.6728	0.5986
-24.3442	104.868	-2.1342	38.3966	107.8393	-0.1745	11.6542	6.9017	0.8004
-10.8975	110.8278	-6.8717	51.348	107.1619	-5.6418	11.5567	9.4196	3.0538
-20.6069	100.8869	-3.9383	43.7672	99.9531	-2.7571	10.6802	5.2321	1.0399
-18.7993	105.8821	-2.2255	45.8569	104.6358	0.4976	13.5896	10.3344	2.5698
-27.6212	103.9203	-1.7591	32.8105	105.9637	-4.4924	8.7906	5.3049	2.0965
-19.0011	112.8176	-4.4453	43.8539	112.9297	-5.5641	9.3832	11.4345	2.4419
-23.9348	105.5	-2.3588	34.6881	109.5674	-4.1534	9.043	7.518	0.2946
-32.6585	100.4293	-6.8528	34.8954	107.7846	-7.9794	7.799	8.7084	2.7832
-12.5903	79.7308	3.3943	38.9551	77.1814	3.2487	13.1292	7.8357	0.7273
-13.1474	104.6251	-11.2979	53.2221	101.6574	-8.2045	14.1711	-5.3198	-4.0332
-31.3976	99.3965	-0.1411	33.9247	105.9159	-2.6921	10.6592	14.1713	0.8115
-26.4941	107.1106	-3.9816	38.2513	109.3577	-2.9849	10.195	15.1448	2.2812

x43	y43	z43	x44	y44	z44	x45	y45	z45
5.9469	13.2618	-0.1446	6.5374	78.5954	1.8714	-1.7317	97.1152	-3.1705
3.5705	22.3308	2.5452	-5.3777	86.1353	3.2982	-13.7947	105.3537	-4.5941
8.4706	19.2786	4.7249	10.9307	90.5653	5.8792	4.8392	107.7848	1.6071
7.2633	20.4116	1.1084	7.0221	93.9405	6.0501	-2.3843	117.0978	0.1698
5.4536	19.9725	4.0815	-4.7668	91.6096	6.4838	-11.4746	103.5299	-0.0344
9.7833	18.356	1.4988	13.5212	83.5797	5.1658	6.7657	105.6321	-0.4788
10.9349	12.9076	-0.9305	4.5055	92.3434	-0.1881	-13.2681	121.1912	3.9615
3.5569	17.7667	1.9472	-7.6807	71.4729	4.4344	-16.502	85.8724	-0.1425
6.3722	29.2328	5.0009	7.8554	96.6607	10.5503	-2.6976	117.6189	3.3532
12.6586	25.9683	0.85	19.9136	102.6403	7.4876	12.7496	130.1345	-2.1957
10.2134	18.7681	0.6767	14.1153	79.708	4.7791	8.33	100.0539	-3.2833
8.4307	19.8632	3.1862	6.8055	92.6255	6.098	-1.548	114.0581	1.1561
10.7359	23.2256	1.0049	19.0143	89.1093	4.7987	14.9272	109.2261	0.1956
9.3962	19.2784	0.7231	10.4361	88.9179	3.9418	1.4762	114.3064	-5.0345
5.0159	20.6697	2.7732	1.3568	81.7471	4.5296	-6.2683	101.2764	-0.2844
6.9109	24.0211	1.531	6.6059	83.5341	6.487	0.0059	106.4771	-2.181
6.6514	18.6281	-0.0388	7.108	86.5432	1.1423	-0.7477	108.9545	-5.6572
7.493	23.4052	0.8722	9.9217	86.76	4.4123	2.4964	113.718	-3.9772
8.079	31.2091	2.2241	0.1181	105.0282	6.1726	-11.9949	129.1011	0.8624
9.0669	26.9493	3.6806	13.7639	93.2635	5.4859	5.5391	119.3636	-0.2217
6.832	26.7212	3.3882	-0.7565	109.3391	5.9929	-14.3839	130.5889	-2.0094
7.7871	11.3277	2.8915	2.9712	75.9744	4.2522	-5.4844	99.1433	-7.8022
9.8572	18.0297	7.7041	8.1574	72.312	9.5789	4.729	86.2671	-3.413
11.5737	11.2988	6.7414	13.0301	74.893	4.3641	5.1566	98.9433	-10.7509
12.124	9.6786	5.2053	10.0664	64.9198	4.0732	1.7859	91.5111	-13.7543
11.2702	12.3722	8.7066	19.5749	69.434	1.9056	11.4123	97.7899	-5.5651
4.0983	19.2776	-0.7172	-12.4725	106.6273	2.7816	-25.0279	127.8773	-8.8108
12.2097	25.1534	3.5817	17.3658	106.2614	-2.4065	6.2464	134.6029	-13.3163
11.6627	17.8941	1.5919	10.0249	101.8493	-3.7151	-4.4412	129.0786	-12.9477
9.503	21.1377	0.6484	8.7968	93.1689	5.0022	-1.6889	113.2135	-4.9167
10.9523	13.7472	0.6608	17.2513	109.1406	1.6025	6.7694	134.6499	-7.7579
9.285	10.0563	-1.2229	11.6236	97.4774	-1.0353	0.042	125.0601	-15.1624
10.883	13.7155	1.2596	6.4437	111.654	4.3903	-4.5224	139.8567	-7.0498
9.3389	14.8513	3.5379	10.687	107.0476	1.5992	0.2813	133.2583	-11.1981
10.1314	11.7286	2.1287	14.0608	107.9626	1.6524	2.3283	138.7806	-11.9424
12.0495	12.4928	1.6648	28.79	79.0946	1.2951	25.8926	103.0281	-2.9503
10.785	18.5643	0.1219	14.2124	96.8004	1.3115	3.7167	115.4464	-3.4388
12.3404	19.8776	-0.2631	13.5781	95.6251	3.7416	1.4428	130.4789	5.7929
11.1106	7.034	-0.0625	22.5172	66.1544	-0.0585	17.8406	87.6135	2.4815
10.0329	16.16	2.2975	7.8341	95.4923	5.1035	-0.7976	115.3572	-5.6495
8.3046	15.7299	2.6775	4.743	91.3044	4.9238	-6.7331	113.4688	-4.7916
11.2077	20.6022	3.997	9.2868	107.402	-0.827	-2.392	136.1726	-15.3291
16.0174	-7.3475	-43.3876	15.5144	77.8468	-23.6728	5.9954	103.355	-30.3227
17.6208	7.385	-46.4242	12.969	79.3101	-28.7256	2.9761	109.1131	-33.6755
13.3695	4.8835	0.4232	10.1392	79.9946	6.1051	-1.9831	106.0246	7.114
10.3386	8.8011	-0.8731	8.5963	68.8395	-2.0421	0.6328	93.243	-7.8824
11.5365	12.8772	-1.7116	13.7185	78.1866	-6.8228	1.4044	104.6185	-6.1129
8.2412	16.6513	0.8932	9.0027	77.9131	-2.4153	-2.2637	103.2439	-2.2457
9.4876	14.0443	1.9731	8.9232	63.7978	-2.5454	0.0464	85.1998	-6.6463
6.9215	18.4629	0.338	2.8345	84.4218	-5.1483	-14.0495	110.0794	-6.9062
7.7346	13.4001	2.6258	8.1931	62.9666	1.2838	-2.186	85.1112	0.9468
9.2239	2.7958	-27.9019	5.1956	48.1073	-19.3057	-3.5873	64.7208	-20.2719
10.9659	15.5575	1.0941	20.8147	111.4868	1.5348	10.5396	136.7725	-10.5766

10.7075	14.0061	0.7338	14.4599	104.7622	-0.1138	4.0123	132.1759	-10.9632
8.9642	18.8688	1.342	9.0269	115.3186	-3.0362	-2.9444	142.8004	-15.0448
8.9861	29.2615	4.211	6.7678	103.9757	1.7922	-2.8413	127.4887	-8.1118
11.7365	16.7117	3.8741	13.0794	106.0651	2.7921	4.8804	136.4097	-10.722
12.1926	-3.6647	-42.7365	10.6389	84.2171	-26.6443	2.3011	108.3066	-33.3563
10.2787	15.0728	0.6927	10.8323	108.2768	-0.6955	2.0498	132.6969	-8.583
14.0877	19.5744	1.9967	25.7413	106.1961	2.9998	17.0355	129.9836	-6.7481
5.9582	28.6697	-0.2395	-7.704	110.4769	-4.0899	-22.5393	135.949	-16.0014
17.8786	20.205	-0.1132	40.261	121.4234	0.6406	33.6653	154.8395	-10.0933
11.447	11.6951	0.6434	21.1389	97.7885	4.1176	12.3137	127.4595	-7.4529
11.7044	15.9808	0.836	7.201	100.3129	2.786	-4.4189	134.5811	-10.7663
12.5242	19.6503	2.6607	19.9097	113.0864	-0.1675	11.2797	141.5376	-10.2565
10.9553	21.0995	1.1044	11.123	102.5473	2.8881	-1.7599	133.3435	6.912
13.5684	17.0565	1.9492	13.0841	95.2341	4.5869	2.1717	131.982	1.9346
8.3278	16.6323	1.988	3.9358	100.9046	2.3022	-5.0798	121.6112	-3.0903
10.7035	19.3747	2.6273	11.6212	110.1786	-1.999	-1.6037	140.3268	-16.1201
9.0984	16.1508	0.133	5.1569	104.4198	2.5615	-7.7599	130.2283	-7.1206
7.7191	16.4358	2.7078	-1.1973	103.0687	-3.0422	-15.282	131.5847	-14.3433
12.6565	9.5294	0.1512	13.6621	87.5023	-3.7737	-1.4615	108.7054	-3.2738
14.6537	-2.0028	-0.3313	19.073	102.1914	-8.0575	6.0159	128.8907	-9.1563
9.8669	18.236	0.4055	1.1354	105.2434	-1.0436	-17.3501	132.3185	-3.1013
10.2001	22.4892	1.3691	5.6749	107.9652	-1.9313	-9.1481	135.0862	-3.0396

x46	y46	z46	x47	y47	z47	x48	y48	z48
13.6353	96.3046	-4.3803	-5.7136	122.4893	16.2373	19.6996	124.7896	17.6346
-1.1967	108.8416	-4.7646	-20.1367	128.5867	14.369	2.778	129.071	13.5233
17.3223	107.8369	-0.2687	1.1234	129.9273	19.8201	26.2882	129.4933	19.1944
15.2619	118.4178	-2.5216	-3.2521	141.6817	23.0894	25.0104	140.967	21.601
5.2024	106.8234	1.7673	-20.0741	125.3084	17.81	4.9381	126.7793	18.8302
21.0365	106.4714	-1.8748	2.5884	132.0063	18.4915	27.6737	132.0068	16.9307
13.9587	124.4174	2.5469	-14.3803	144.0733	20.61	14.3719	147.5629	18.0148
-4.1859	87.3701	0.8178	-27.2947	107.4641	17.0216	-2.0755	111.7169	17.9194
15.7746	117.0783	1.4203	-5.7583	143.5845	24.3109	21.0298	143.3674	22.9046
33.0569	127.442	-2.4647	13.4032	164.9257	20.1559	40.845	160.9987	18.6739
23.0134	99.5026	-3.1199	5.9733	124.5131	18.2137	29.285	121.5345	16.9729
15.0844	113.6673	0.0221	-8.1473	142.5054	19.8058	21.6439	142.8541	18.9776
28.2077	106.1017	0.8005	11.2028	135.5411	18.8809	37.4646	134.8483	17.4303
18.1209	114.9375	-4.6356	-2.0742	138.2784	14.6116	22.887	139.4451	14.6721
7.818	102.8684	-0.14	-15.5922	127.6968	15.6167	9.4877	128.0095	17.4268
14.2933	107.5474	-1.7608	-5.803	129.8163	17.1748	19.124	130.8333	16.621
14.6617	108.8195	-5.8454	-4.7447	135.3622	14.7163	20.7944	134.679	14.2401
20.067	114.1128	-5.278	-0.438	132.4756	15.7851	25.4828	130.6143	16.4447
8.0746	130.8775	3.4823	-20.6822	163.1422	21.4458	10.6285	165.6757	21.0841
23.6566	118.4688	-0.7453	5.1012	153.7874	19.0503	31.1244	152.3419	17.0464
9.4441	131.1461	-0.0854	-23.8402	169.4374	20.5881	9.5567	171.0792	20.0755
8.9063	100.2594	-6.7199	-11.2499	122.6497	14.4008	9.2092	124.5911	15.0632
16.8255	87.6313	-3.0493	-0.7083	104.0066	16.3839	16.3165	105.4758	18.8906
21.4051	100.2356	-11.0441	4.0064	123.0385	17.0908	25.3005	121.4011	13.4729
17.8225	87.4184	-12.3762	-0.8749	108.619	12.501	18.9311	108.7583	12.9323
38.8292	94.0722	-3.8882	16.056	115.145	9.2449	37.2735	115.5595	9.4381
-9.1567	129.2125	-9.475	-33.6914	152.2672	22.553	-9.8639	156.675	23.4015
30.748	140.152	-12.8059	7.0557	158.3186	15.872	32.4172	159.0431	17.9964
21.2427	136.2508	-13.1535	-4.1892	152.2168	15.6693	20.8724	154.9449	16.9034
18.7572	118.6168	-6.9444	-2.59	141.748	18.0266	21.4569	141.9226	18.4062
29.2073	133.1304	-7.4397	8.2807	165.7022	16.6485	35.0889	161.4817	16.6639
25.0622	123.7049	-16.2028	2.7747	148.8922	17.0615	27.6172	148.1299	15.83
16.1488	142.7384	-5.4338	-12.1903	169.6333	25.7098	15.2593	169.496	25.5033
21.1433	129.8025	-9.7656	0.7752	166.5578	22.0378	23.2597	162.5515	22.2288
26.6631	137.7267	-12.8853	5.7298	164.3273	22.6943	28.0992	161.9307	21.2909
41.2465	99.6836	-4.7083	32.0515	127.1013	12.9669	54.3305	121.3498	13.434
23.7716	115.4293	-4.8771	4.498	139.1591	16.6342	30.4758	139.2785	17.4137
27.1973	131.1832	4.1738	3.1645	149.0172	17.95	25.3948	148.9911	17.7044
35.5872	83.4377	-1.0921	21.0472	102.8032	12.3221	42.2464	99.5656	9.9327
15.5978	116.4398	-5.1961	-7.4094	146.1687	16.5022	22.4422	146.1775	15.2986
13.5969	114.1747	-6.4922	-10.8822	130.6637	19.7398	14.3002	133.6424	20.8736
17.665	136.1324	-16.9324	-3.3889	160.7882	16.9017	21.2077	158.7882	15.0697
25.7708	104.9391	-28.7885	2.9723	122.7609	1.2838	24.7369	123.5093	9.7616
20.395	108.115	-34.2305	-2.4002	131.9699	-0.9606	20.3409	132.1698	-0.4851
17.5234	108.4086	7.1114	-4.8625	129.0719	19.6991	16.4656	131.7692	21.3968
21.5249	95.4371	-6.0639	-0.2797	106.4275	3.9411	20.848	108.1285	5.9598
29.9176	103.4863	-6.3229	3.064	117.5697	12.4323	29.2386	116.0121	12.5388
20.8092	102.1483	-1.9204	-2.2274	113.8889	9.1452	21.0399	113.5902	8.2959
20.8975	84.1445	-6.4105	0.0168	96.7685	5.7553	21.1396	96.3613	6.5659
16.1855	113.2503	-6.2951	-12.6139	121.9767	8.8725	11.084	123.5261	9.6969
18.7536	85.0799	-1.3011	-0.2398	98.0935	8.8454	18.7829	98.5604	8.2208
12.8179	65.0321	-20.4404	-6.3502	73.7368	-1.6191	12.0414	76.2772	0.1669
34.1603	134.9945	-9.2538	13.398	162.6821	23.491	38.0247	159.7686	21.434

25.7492	130.476	-12.183	5.9337	160.0204	18.0779	29.335	156.389	14.726
17.9896	141.3113	-14.8574	-5.4916	173.0354	18.8094	19.1236	172.6895	18.5193
14.5246	127.6262	-8.421	-4.9574	158.4169	19.5746	16.6981	158.2206	16.485
27.2354	139.4963	-10.4823	2.4073	169.0251	21.7039	28.4765	166.0557	20.0002
20.0884	108.9405	-30.8749	-2.7641	131.2277	0.3375	22.4034	131.2835	1.3051
21.8837	134.9355	-9.5337	0.1048	160.0767	16.3092	25.8704	159.2185	16.8838
40.9484	125.2773	-8.7818	21.1926	150.2357	23.8306	47.2018	146.8911	22.2024
-0.3773	136.9463	-13.5711	-28.9347	161.9318	10.4923	-1.8657	164.9265	13.5751
61.7492	149.1546	-12.6351	40.797	178.1569	21.5517	68.899	170.5737	22.003
30.1865	118.0649	-12.0608	18.1086	151.4806	25.3994	41.9211	150.1347	21.98
17.6551	131.4697	-10.1768	-9.2665	153.6987	19.3059	16.6008	156.2161	19.9614
34.7483	138.4926	-12.2467	13.7551	169.3846	25.9331	34.7163	164.6151	23.726
26.2432	131.7197	6.9457	-0.2749	152.1402	21.3832	24.5193	151.602	22.3263
28.852	133.5876	3.4938	2.1617	153.4441	19.2616	26.0459	151.6125	19.7454
12.0177	122.9172	-5.2238	-12.8319	149.5757	21.7289	15.7726	153.5727	23.6889
23.6311	143.5381	-16.615	0.0998	171.1296	15.5326	27.0086	172.0311	16.5294
15.2739	131.5275	-8.418	-11.8131	162.4315	19.4102	17.0298	161.4756	20.2567
9.849	132.2656	-13.6731	-19.0912	158.7238	17.6366	9.3278	162.7486	18.0796
28.455	109.0728	-4.7311	3.6291	125.244	10.3512	24.9169	125.0034	10.4567
36.7462	130.0555	-6.0076	9.8649	150.5926	10.5682	34.0822	147.9116	13.0331
13.7684	134.5444	-3.0233	-17.999	150.3499	13.3708	-14.0785	151.8859	14.8133
19.5002	133.6702	-1.2718	-7.596	155.3864	16.6149	15.8622	156.1292	17.8304

x49	y49	z49	x50	y50	z50	x51	y51	z51
4.3838	97.2145	19.7296	-11.7382	129.8838	23.4714	25.6768	129.5099	23.353
-4.6096	98.7425	21.0876	-27.0635	135.5319	18.8578	8.1452	138.7726	18.8395
10.793	115.2453	17.714	-5.6965	139.1513	26.4227	32.6129	136.4983	24.2584
8.0064	104.9938	23.19	-6.6003	155.9593	24.9785	29.2016	155.3302	20.3416
-6.1417	89.5855	22.4482	-29.0549	131.6401	22.176	9.2411	134.9115	24.9713
16.1189	99.1176	23.0253	-2.8948	139.947	23.3117	32.9377	138.3912	21.5289
3.7187	106.7731	25.5328	-19.6994	152.7155	23.8566	20.358	155.2523	22.3314
-7.6774	78.5833	19.8483	-35.5833	113.193	22.2869	0.8551	121.3739	21.807
6.823	108.8515	33.4645	-11.7287	151.7725	30.9787	26.341	152.4937	29.5837
21.1163	110.7265	33.1714	6.3579	168.0227	29.5328	48.0408	165.3302	28.2018
16.5228	88.0299	21.6138	1.127	129.4601	23.5517	36.5769	126.177	22.7049
7.3146	106.4318	28.4818	-14.3992	148.0092	26.5899	29.5837	146.9766	25.4775
19.5007	99.252	23.1455	5.445	142.5972	23.4556	45.8199	139.6632	22.6939
9.8631	98.5071	22.9329	-8.0694	143.4473	22.1216	28.5305	143.9879	20.7206
0.4351	90.2483	22.8992	-21.6633	132.7211	19.8816	13.8536	135.5387	22.0239
6.1456	91.516	20.6957	-11.8072	136.6808	22.5567	26.9852	137.2863	22.9494
8.2739	103.191	19.0425	-9.2059	142.1106	21.0182	27.2054	142.5406	20.1864
10.6099	101.1405	20.9217	-6.5902	140.7035	22.231	31.6845	139.5887	21.361
-2.2019	136.2379	22.2204	-27.7995	171.1189	27.2681	16.3762	173.2815	26.5949
16.0388	113.2493	27.3595	-0.5264	159.3834	26.0115	37.3145	156.0367	23.0214
-2.3387	125.7437	32.0075	-28.6045	166.3913	24.1916	16.7111	169.6905	25.5589
2.4416	86.4851	23.6197	-17.1708	130.575	19.179	14.1606	132.2382	20.7869
6.8144	77.7564	33.9267	-6.4383	109.2561	19.9716	21.3353	110.6086	23.5627
17.8256	85.4413	26.9604	-0.0657	132.3314	19.9048	31.8515	131.0418	17.8292
11.0964	74.6525	30.0994	-5.2367	116.8077	18.2406	22.4449	115.9371	17.2178
19.5649	78.5622	28.8533	10.9648	124.7831	19.4791	43.1627	120.9285	20.1275
-13.209	108.3519	26.4306	-39.6294	154.544	26.4941	-5.8067	161.1325	25.753
16.21	110.4664	22.9055	1.167	162.8476	22.7869	37.9849	162.4373	24.4441
8.5036	105.9978	23.2732	-10.7544	157.3325	23.1016	26.7806	158.7835	23.2666
9.761	104.4344	27.0522	-11.6536	147.625	25.4341	26.4812	147.5024	22.9496
18.5762	117.9546	27.1796	4.3959	170.2436	24.7186	40.793	165.0162	23.4356
13.2455	107.5708	22.988	-4.9336	154.6079	23.0318	35.8399	154.5664	21.1188
3.9813	121.8048	33.5803	-20.2385	176.803	30.0246	21.5351	177.7323	32.8744
11.3439	117.5761	30.7018	-7.9275	171.5491	28.348	30.7786	170.9353	29.9681
13.6496	120.5939	31.727	-2.0258	170.3511	27.637	34.1853	168.0441	27.3601
33.171	83.856	26.9766	27.7196	130.7546	22.7918	60.4222	120.31	19.6375
17.2358	100.7567	27.7922	1.0838	143.7525	22.9469	33.6308	143.1908	21.6694
12.452	109.4806	24.467	-2.0298	153.7581	21.7475	31.6593	153.5415	22.466
26.944	66.1661	26.9228	17.0194	110.5192	17.2192	49.0285	103.6989	12.8749
8.3101	108.4448	27.9328	-11.5404	150.8484	21.1733	27.5469	151.6082	19.3045
3.7404	102.2389	27.6791	-19.7681	141.6003	25.6152	20.7423	143.4527	26.0561
12.1948	113.8825	33.3807	-5.1098	163.0574	19.9593	27.6326	165.9948	23.5062
13.6433	86.8227	2.5833	-3.0986	125.2117	7.7261	30.3657	128.833	11.328
9.5574	95.057	2.571	-10.8528	138.1337	9.5516	27.1866	137.7709	10.4378
6.7859	98.4041	31.618	-10.3684	133.679	23.6168	19.9606	136.4951	24.8291
9.1941	70.746	14.0587	-8.8171	109.9989	13.6272	24.7961	109.5201	13.6015
15.3584	85.9032	12.7027	0.2309	121.5146	15.1012	34.1201	119.4045	13.3682
10.1248	83.1334	17.3393	-5.8818	120.6883	13.1305	27.4567	119.7977	12.9729
10.2392	63.9983	11.0399	-5.2094	102.9098	10.4874	27.483	102.3896	11.5066
1.2475	85.252	14.5195	-17.323	124.9876	14.4221	16.6538	125.9468	15.3043
9.3883	69.0182	14.143	-6.8139	101.2657	15.2706	25.2591	101.2156	13.8705
5.2974	48.7137	-4.2896	-13.1453	77.1048	3.4944	17.2308	78.2203	3.5881
23.0105	121.9412	29.4284	8.0527	169.6017	30.2426	45.0237	166.0541	29.6761

18.2923	115.8371	29.7024	-1.2916	167.7087	26.4064	37.2371	164.5265	24.9354
8.2009	129.5653	30.5856	-14.2276	179.7492	28.7258	26.1368	181.7109	27.1721
8.8607	113.8285	29.3799	-12.8315	164.1309	26.5318	23.8624	166.048	25.1689
13.8165	130.2871	36.2507	-5.2781	175.0794	27.9009	35.0543	173.8431	28.6898
9.5372	79.0968	-1.1727	-7.491	130.7989	6.1296	25.8874	130.6693	5.9089
12.912	108.147	25.9646	-7.484	165.0789	24.8296	32.498	162.8963	24.0938
26.8717	101.6761	27.233	17.7767	156.3033	27.8746	52.3116	149.4812	25.3103
-9.1025	117.9519	19.768	-35.3455	161.6536	16.2867	1.6931	169.0315	17.7571
43.4762	120.9879	37.0729	36.3577	185.0368	30.1519	75.9634	177.4202	28.2925
26.9082	108.4107	32.0816	10.1493	159.1524	34.2236	50.1486	154.3414	29.367
5.0071	111.436	27.9434	-15.1114	159.4908	25.7226	22.0109	159.1777	26.9602
20.0513	119.7408	35.2238	5.7344	173.1975	31.7931	42.2934	170.0144	31.6531
11.2836	112.1294	32.0464	-6.9706	158.495	29.2353	31.1598	158.4516	29.5016
13.0264	116.3926	30.3471	-3.8393	160.9579	24.7629	33.629	158.4126	26.0556
3.6362	111.1771	28.4029	-18.6018	156.5748	26.8404	20.1318	157.3014	27.303
14.2946	125.1061	26.644	-6.8429	173.399	22.8726	31.3346	176.0139	21.4006
3.3719	114.2213	31.0705	-18.2287	165.7955	25.445	21.7219	166.2601	25.0931
-0.4769	121.2262	27.651	-27.5084	163.6666	23.5261	17.0874	166.3043	23.823
15.2991	91.189	16.0173	-0.1134	127.8677	15.9496	31.2314	129.4679	15.9701
18.1506	105.7844	21.262	4.1575	155.536	17.9877	39.6696	153.9611	19.7582
0.9808	109.9816	22.8727	-25.015	158.4987	20.8582	15.0225	167.3264	19.1058
5.4018	119.2242	22.312	-14.6276	160.8553	21.7745	21.8898	163.2669	22.5569

x52	y52	z52	x53	y53	z53	x54	y54	z54
-2.9613	159.0804	-1.471	16.9588	161.7272	-1.4353	-17.2789	158.9625	-18.1906
-23.1311	161.3678	-3.937	-1.5516	166.1167	-3.7633	-36.3146	160.0187	-22.4939
1.7212	167.9817	-1.5961	24.8028	166.5377	-1.084	-14.7136	167.9276	-16.8712
-1.2857	185.2348	-1.3294	20.1955	184.966	-2.7048	-21.3176	186.6508	-15.0378
-20.4336	160.2312	0.3322	0.9504	161.8222	3.1626	-29.9365	155.7294	-18.6872
2.617	167.9708	-0.5234	28.0106	167.1193	-1.267	-9.1468	164.2671	-19.1407
-17.2303	184.7885	2.3073	11.0356	187.6024	0.8921	-32.1439	177.7137	-21.8979
-35.0089	141.3546	0.7744	-10.4505	147.1924	-0.4397	-46.7352	133.452	-14.9479
-5.2042	180.5298	1.0397	19.5488	182.8387	0.0156	-21.2556	179.7911	-22.5281
15.9505	207.4472	3.8024	46.7115	203.8524	3.1899	-9.8313	215.1215	-24.6938
6.424	158.6271	1.0487	33.5541	156.8832	-0.5647	-7.1343	153.196	-17.8685
-6.704	181.0638	1.4755	20.9677	180.8633	0.5549	-25.4549	178.7147	-19.9311
14.3797	169.1371	0.7469	40.9281	168.4104	-0.3885	3.128	168.3273	-17.9226
-2.7235	178.9995	-0.0177	22.5222	176.334	0.2285	-16.5711	175.8566	-20.6287
-16.1049	161.6264	-3.5037	8.2874	161.8711	-1.7923	-27.3028	158.3917	-21.2061
-6.3893	165.2892	-0.1757	20.8549	164.0693	-0.5564	-18.8999	165.0285	-18.6137
-4.8373	169.5476	-0.89	20.4186	169.5929	-1.4347	-18.9016	168.6799	-20.4129
0.6445	167.5079	-0.2415	25.4192	165.6222	-1.7338	-11.173	165.5583	-19.0083
-24.6086	203.6294	3.2478	5.7064	207.7364	2.7628	-43.4874	200.2116	-24.8858
6.8452	193.0353	-0.4115	32.216	192.1569	-4.2087	-11.5326	194.86	-24.1346
-26.7154	209.4917	-4.7661	4.0754	211.7614	-3.605	-42.375	209.4879	-26.6173
-15.4105	155.0708	1.2003	9.1867	158.4738	1.7796	-22.6801	153.5583	-23.9087
-4.3141	135.2105	-0.1912	19.1738	137.5467	4.7947	-8.6991	133.086	-21.1816
3.9506	158.9318	1.3775	25.3501	156.4073	-0.9259	-8.8092	159.576	-23.5026
-2.8394	150.8292	0.7699	20.5462	151.1531	-0.3855	-16.4256	153.3355	-27.3959
20.2905	151.9415	-2.0946	46.0603	149.0192	-0.1499	8.6479	160.9727	-29.77
-50.1257	183.068	7.0779	-9.6424	190.5938	4.3842	-60.2515	181.8435	-18.4036
5.6083	196.2678	1.5822	36.2467	197.9721	1.4935	-8.7517	195.1602	-26.8015
-14.3319	190.6641	1.165	27.2812	194.8756	0.2335	-23.2748	188.3405	-25.7937
-13.4289	176.0801	2.4291	27.1629	176.5492	1.0503	-24.9612	171.8809	-19.7749
5.8912	207.8782	3.465	45.8912	204.8827	1.9369	-8.5741	204.7168	-23.1638
0.8525	191.2827	3.4703	30.9975	188.9492	1.4025	-15.5815	182.2021	-24.3177
-11.0611	213.2822	6.1045	13.3039	212.7627	5.6049	-26.2714	203.4981	-28.1028
-0.9932	207.6786	3.5425	26.1864	204.3459	3.1342	-20.0926	207.7775	-22.667
2.6218	205.3762	4.5726	30.8846	204.2327	4.48	-13.7918	203.6749	-25.9888
33.1774	164.3333	1.3247	67.0238	153.7644	-2.092	20.2771	164.6529	-18.8606
1.5032	173.3306	2.8793	33.6541	173.3534	0.5639	-9.0603	172.0596	-18.3156
2.6314	179.473	2.2722	28.6665	179.7208	2.6709	-11.5339	178.3407	-19.871
25.5221	136.1469	0.462	47.1132	129.761	-3.4336	12.8458	137.9882	-17.9371
-6.9623	178.0647	0.7658	19.8521	179.0608	0.2808	-24.8555	180.7943	-25.1522
-14.0076	165.1791	2.2934	13.0732	165.7923	3.6772	-26.1078	162.9406	-18.0468
-5.1274	206.1178	0.7881	24.5543	203.2807	0.9883	-26.2365	207.6724	-24.7255
-4.0704	151.2933	-4.9268	29.0931	154.4214	-4.0258	-16.2433	162.1787	-29.7515
-7.5976	163.7156	-6.2112	27.5361	164.8022	-5.6964	-26.2223	183.4068	-30.922
-10.796	165.326	0.2342	13.6872	167.7234	1.264	-24.7663	168.1262	-26.2159
-4.3118	138.8839	-4.7158	19.952	138.4416	-3.177	-19.8386	141.0438	-14.2086
6.4153	145.6388	-0.7109	28.2609	145.1858	-1.937	-7.9302	143.7421	-18.6835
-1.0967	145.6195	-2.8113	24.5508	144.8531	0.1792	-12.6119	144.5053	-18.807
-0.0908	126.2871	-3.0631	23.4072	124.8779	-1.5177	-9.9663	124.5745	-18.8916
-12.0306	151.7584	-3.0813	9.2577	154.7186	-2.614	-24.8796	148.0368	-21.629
-1.3099	124.9302	-1.9514	18.8214	124.2318	-2.6426	-12.7551	124.9902	-15.049
-9.1388	94.3954	-7.5581	12.258	95.9685	-7.5051	-19.7799	108.4786	-17.1087
15.0933	204.2031	3.4574	43.433	199.2073	1.6807	-0.3756	196.8265	-22.2605

5.5541	200.2063	5.5877	29.8231	196.932	1.5201	-13.5084	191.223	-18.6593
-11.5524	221.4726	1.3911	22.6434	219.4557	0.0038	-26.1281	216.5018	-31.5912
-10.0825	195.7403	3.8358	16.9415	195.3155	1.6965	-25.7104	189.707	-19.059
9.9756	214.735	-2.4284	32.4788	210.0386	1.3885	-8.499	200.9963	-28.5144
-8.866	174.3594	-1.6829	28.6228	172.5873	-3.46	-20.401	176.6024	-30.0868
-1.6344	199.788	1.844	31.1848	197.3844	0.967	-15.5209	199.0944	-22.9125
13.8568	185.8025	3.9118	60.8354	180.4477	-0.9642	4.5089	193.3793	-16.7077
-43.4724	198.8913	-0.8575	-4.0306	205.1717	1.6667	-56.2353	199.668	-23.515
47.2146	223.95	2.7506	79.2041	214.9506	2.2846	28.5894	234.9301	-26.5915
15.4279	189.7122	7.923	46.0852	187.3924	2.4251	-4.8791	189.4263	-18.4015
-12.1344	196.1863	-0.8966	17.5398	198.7546	0.2669	-30.1163	195.0117	-25.6592
14.8255	208.0538	3.6312	45.4252	201.133	5.1069	-3.2781	204.1557	-25.7656
-1.5194	190.1435	1.3792	27.6219	189.9252	1.739	-22.4456	193.7696	-18.8445
2.4915	192.476	0.5589	28.2709	192.2674	0.6426	-11.5213	190.2824	-28.9087
-15.4191	186.2456	6.5717	16.1417	187.3465	6.902	-26.7885	187.387	-19.6502
-10.8695	214.4394	3.2355	28.961	215.8683	1.344	-25.3664	216.5239	-25.1235
-19.5776	200.3618	4.6435	20.9881	204.1924	4.3958	-31.054	199.8303	-26.0474
-27.9365	199.5437	3.1107	11.3283	201.0393	1.8662	-36.8135	193.4994	-22.8887
3.1269	155.2344	0.7538	25.4506	155.1018	0.5156	-14.8016	149.5765	-19.1226
9.5976	184.2561	-3.5646	38.4903	184.3426	-3.044	-4.2842	192.4947	-30.3976
-21.1271	186.1806	0.2086	4.2384	190.5924	-0.9857	-37.893	183.3271	-24.9044
-11.35	192.3972	0.947	15.4708	194.6337	0.4758	-24.2783	190.0881	-23.2704

x55	y55	z55	x56	y56	z56	x57	y57	z57
31.4757	162.1303	-18.9363	6.6496	160.6859	-9.75	-4.2413	173.0338	-14.3039
11.2401	165.4029	-22.6494	-12.796	163.4354	-15.3762	-25.034	174.5892	-22.1456
33.2161	165.7091	-19.5036	9.8503	169.2688	-11.1384	-2.657	182.2487	-17.0453
32.8967	182.8611	-23.3984	8.6965	186.9795	-13.6305	-4.198	199.1767	-18.3372
15.9571	160.5297	-14.301	-7.6062	160.477	-8.8932	-21.696	171.5656	-16.1924
38.1904	163.244	-21.7963	14.4101	167.0218	-10.8808	1.7597	178.8354	-16.8923
23.9767	181.811	-24.436	-3.5555	183.2964	-10.1401	-18.4696	193.8996	-18.3547
2.4662	144.2685	-19.9841	-22.4483	142.3103	-9.8621	-35.8723	150.7936	-16.972
36.7072	179.8498	-23.3072	7.7707	184.2154	-11.1568	-5.1621	195.1376	-19.7181
67.7948	200.3703	-26.3388	30.5819	210.6228	-11.9165	18.3273	227.9288	-19.2795
43.6995	147.5148	-20.5648	19.3295	153.2357	-10.4794	8.4994	169.0955	-17.6251
40.9645	178.7078	-22.1559	6.4815	180.8707	-15.6208	-4.1815	194.1346	-23.9624
50.9259	165.6432	-19.43	26.4237	165.8792	-10.3459	15.0389	179.6027	-16.5169
34.8203	176.3631	-20.8173	9.9389	178.7461	-13.5425	-0.6441	185.2499	-20.1338
23.0579	161.5409	-17.2152	-2.5614	159.054	-11.5055	-14.6391	173.8767	-18.9171
33.6339	164.7263	-19.8182	8.1583	162.7825	-10.775	-4.013	175.8211	-18.6873
32.7588	167.3528	-19.9153	7.2231	168.2086	-10.3142	-3.444	183.4145	-16.5763
39.5925	166.0358	-17.0469	13.3467	164.7534	-9.2543	1.5327	177.3715	-16.7133
24.1912	206.3441	-24.5397	-9.3906	207.1347	-15.4437	-25.1572	220.478	-22.5827
44.9169	190.4709	-28.3956	18.3589	193.5981	-15.2628	5.7645	208.1861	-20.1346
20.8506	214.799	-28.626	-11.1054	212.7746	-12.7068	-23.6043	225.4636	-21.1713
19.3903	157.9845	-22.1313	-2.9866	157.7509	-8.5739	-14.9802	172.0955	-14.8906
28.4401	137.6165	-17.2926	8.3254	137.5894	-8.8756	-0.4251	150.8137	-17.4861
32.5794	156.8066	-26.5322	13.2962	161.8858	-11.0362	1.563	176.7662	-18.0015
32.1345	149.4508	-27.6093	8.5135	154.2682	-13.9295	-1.1647	166.7519	-22.5057
60.2822	151.2655	-25.5929	34.8949	153.6398	-14.3681	24.3199	173.3191	-19.9407
-3.0476	193.4654	-25.3585	-30.2946	186.7737	-10.7861	-47.0531	200.0325	-21.941
53.0216	196.972	-24.6977	21.8993	198.9428	-13.0677	8.9583	214.6581	-21.7846
38.6962	194.0614	-25.4876	6.2406	194.9717	-12.7886	-8.7556	208.4503	-21.2354
36.9082	174.6924	-21.9737	5.3487	176.6118	-11.2359	-8.1962	190.6313	-18.7852
53.4138	201.5517	-26.4262	24.4849	204.0951	-12.7351	12.1255	222.392	-20.5027
41.2057	181.4396	-25.9799	14.9861	189.5206	-12.1704	0.2689	205.0626	-20.3759
32.8622	203.7977	-20.3415	2.1834	211.6613	-10.0398	-12.0404	226.7952	-21.05
44.773	211.2795	-21.4933	11.8774	203.4401	-11.2283	-0.9824	222.1144	-23.0525
48.0374	204.5213	-27.0165	16.5484	205.7581	-10.0438	3.5799	223.4543	-20.1651
76.3639	150.6859	-24.497	49.1935	159.9103	-13.8815	41.3566	177.5567	-18.2661
40.9734	172.2715	-21.7295	15.6187	172.1235	-10.2953	4.7787	182.5246	-15.8009
42.2812	176.2556	-19.4184	15.8238	183.8391	-8.4816	5.0197	193.2369	-19.5093
54.9151	132.3448	-24.8939	33.8162	134.8469	-12.3187	27.6743	150.373	-16.902
37.0622	182.4628	-30.3274	5.9204	185.0063	-12.5456	-6.0625	198.5494	-20.6598
24.2673	164.6615	-17.9562	0.4085	164.9055	-8.8164	-12.2418	179.7432	-14.4036
42.1004	207.0661	-25.6216	8.084	205.616	-12.5259	-3.9016	223.974	-22.6381
45.4419	162.7101	-29.3025	0.5523	180.2027	-23.5085	25.6535	181.1818	-21.6704
49.1677	185.1698	-29.4079	-2.3137	201.9466	-25.2589	24.376	201.6473	-24.0842
30.4123	170.4156	-24.226	1.4966	165.6966	-11.5329	-10.5542	183.768	-22.2125
36.082	144.5761	-11.0342	8.0388	143.3371	-13.4826	-0.97	153.9119	-19.0904
40.903	142.3785	-22.1993	17.0388	145.9458	-10.9705	8.1206	157.7077	-15.7217
36.0176	141.5481	-18.4561	11.2899	144.9162	-11.7442	3.9861	156.4708	-14.9542
37.099	123.0806	-16.544	12.0753	127.5705	-11.8918	3.5414	139.3264	-16.2718
24.9108	151.0687	-21.1182	-0.6529	153.8419	-12.8994	-10.3496	165.0058	-17.2181
32.9964	127.6715	-12.7412	8.2101	126.1857	-11.6868	0.8315	132.7919	-15.9945
22.6266	111.6828	-16.245	-8.8955	117.0045	-14.2289	8.7762	118.4338	-14.171
51.4385	186.6469	-24.6363	28.5781	201.0637	-10.8777	18.4555	217.0249	-20.4826

45.3092	190.9561	-20.9434	14.4376	198.4914	-11.5733	2.5173	212.5279	-19.2869
37.1663	216.701	-31.5453	4.4118	222.4996	-12.8091	-8.5933	242.2114	-21.7006
32.4784	193.222	-19.8346	3.0519	196.5906	-11.5093	-9.7584	210.0493	-19.6134
47.3675	199.7919	-25.4146	17.951	211.1945	-14.6379	4.4198	230.5215	-25.7536
39.5755	177.6226	-29.3398	9.4806	178.1763	-16.9018	-0.5256	198.2388	-18.4703
42.3543	199.5725	-23.5826	12.8284	201.4919	-12.8709	2.668	219.3168	-20.0991
66.9485	180.9949	-21.87	35.7616	179.9855	-8.8882	24.0783	198.552	-15.938
9.1933	214.9191	-21.4597	-23.1771	204.5875	-13.7472	-37.4081	216.5045	-21.2245
101.8548	219.4578	-30.6824	64.0205	225.791	-11.1491	56.0873	243.4408	-24.261
60.9434	182.346	-25.9598	28.5457	186.3776	-8.2954	15.6022	202.9007	-19.5323
38.4144	199.7714	-22.6058	3.4286	195.5657	-13.2594	-10.3889	209.9747	-24.0952
62.327	196.7759	-24.3561	30.2357	203.1703	-8.3238	19.2657	223.2531	-21.6374
46.1473	192.0553	-19.6482	12.6633	188.4719	-11.2951	1.4419	201.9988	-20.1507
45.9166	189.0019	-27.3252	15.6697	189.2706	-9.6152	3.9913	206.7842	-22.1898
24.4444	190.2544	-18.8153	-0.809	189.4871	-6.6752	-12.5091	201.4864	-15.6797
43.6654	220.5462	-27.2388	8.4609	215.6066	-13.2916	-4.6774	231.8445	-22.4741
30.0827	203.3053	-26.3361	-0.2699	202.7644	-9.7469	-14.4882	218.4576	-19.3417
23.0995	197.3942	-23.5169	-7.5576	201.3276	-11.471	-20.0613	214.5204	-18.7233
43.4599	150.4315	-18.6398	14.4541	156.9744	-11.5809	5.3754	167.9334	-16.0281
57.1481	189.027	-26.2656	25.6105	188.4991	-15.1419	13.2255	207.2646	-24.2336
18.6526	192.059	-24.0429	-10.6578	191.9538	-11.0349	-24.7277	203.801	-20.5566
29.2529	191.2723	-24.7275	2.246	195.8486	-11.2631	-10.6022	208.2373	-18.2903

x58	y58	z58
17.4751	174.2064	-14.9277
-2.7381	178.331	-22.4021
23.0175	182.6506	-18.4153
21.7474	200.3339	-21.1907
4.2212	174.9515	-12.3383
27.7612	179.829	-18.4959
8.4431	198.1467	-19.4549
-12.8301	155.607	-16.029
19.9207	195.2794	-20.044
44.6754	225.9093	-19.5449
33.4451	166.7135	-18.6133
20.1125	195.684	-22.6303
41.6055	178.6296	-17.9104
20.5459	186.2068	-19.3733
10.0906	175.5517	-16.4538
20.3607	175.635	-18.3346
19.9168	183.086	-16.9799
25.1856	176.8985	-15.9089
2.3547	222.932	-24.178
29.7957	206.9067	-22.435
-1.9031	229.6365	-19.7191
5.8657	175.3753	-14.1652
18.455	151.7555	-14.9838
21.4694	174.7351	-20.2863
19.6592	166.9564	-23.0285
48.5445	170.7551	-18.5122
-21.2947	206.7546	-22.9428
35.3374	215.4806	-20.7875
17.0201	212.6615	-20.368
18.0743	192.4749	-19.2956
37.1321	221.2504	-20.9861
27.0625	205.4471	-20.69
16.7445	228.3064	-19.7939
26.283	221.923	-21.8904
28.6587	222.063	-20.6574
65.6715	172.0626	-20.3964
27.0749	186.75	-17.7263
27.6433	194.3303	-18.4096
43.9182	147.2509	-19.5677
17.4776	199.0719	-21.3448
9.7179	181.4855	-14.2757
19.6402	224.1094	-23.6133
13.0835	164.1789	-16.8515
9.9902	180.922	-17.394
12.5183	187.2759	-20.3766
17.3711	157.376	-16.6482
26.8048	155.5467	-16.8734
19.9086	155.4342	-15.3278
21.7634	138.0728	-15.6838
9.0603	165.6679	-17.3069
15.8253	132.8093	-17.1261
0.2987	105.5113	-13.8673
40.6718	213.0597	-22.0822

26.0215	211.375	-21.5015
18.0078	241.4867	-21.5202
14.2054	211.5774	-19.4728
33.8064	229.8099	-24.1893
18.1676	196.2505	-18.1212
25.309	217.3689	-20.9026
50.4757	193.4301	-19.8521
-12.9151	222.685	-19.0096
80.6101	240.0558	-24.3379
42.2494	200.046	-22.2597
16.8659	212.2807	-23.3702
46.5282	218.3597	-20.8142
23.5368	204.7758	-21.3031
28.9319	207.65	-22.0537
10.4944	201.1773	-15.1171
18.3111	234.7329	-23.0177
11.6872	221.8728	-18.9168
4.0366	215.1007	-19.046
21.7873	169.2119	-15.4329
38.0093	205.2484	-24.5573
-0.7412	207.8512	-20.8035
13.0957	208.3196	-18.8748

S2. Landmark descriptions.

No.	Landmark descriptions*
1-2	Anterior-most point of the nasal-premaxilla suture
3-4	Maxillary-nasal suture, anterior-most point
5-6	Dorsoposterior edge of the infraorbital foramen
7-8	Anterior-most point of the zygomatic arch by zygo-maxillary suture, inferior edge
9	Nasion, nasal-frontal suture, midline
10-11	Lacrimal-maxillary suture, medial end
12-13	Zygomatic-lacrimal suture on the orbital margin
14-15	Lacrimal-frontal suture on the orbital margin
16-17	Supraorbital foramina
18-19	Anterior tip of the temporal-zygomatic suture, in lateral view
20-21	Posterior tip of the temporal-zygomatic suture, in lateral view
22-23	Fronto-zygomatic suture on posterior orbit, midpoint.
24-25	Articular eminence, ventral-most point
26-27	Highest point of the vertically oriented posterior margin of the zygomatic process
28-29	Posterior-most point of the posterior margin of the zygomatic process
30	Bregma, intersection of interfrontal and interparietal suture
31	Inion, highest projection of the external occipital protuberance, midline
32-33	Nuchal crest edge, by temporal-occipital suture
34 (REF)	Opisthion, dorsal margin of the foramen magnum, midpoint
35	Midline between incisive bones, anterior most point (ventral view)
36-37 (REFS)	Suture between incisive bone and maxilla in lateral view
38-39	Anterior edge of P2 alveolus
40-41	Posterior edge of M3 alveolus
42	Posteriormost point of the incisive canal
43	Posterior tip of the palatine process of the incisive bone, midline
44	Posterior tip of the palatine-palatine suture, midline
45-46	Distal tip of the pterygoid hamulus
47-48	Anterior edge of the caudal alar foramen
49	Posterior tip of the vomer at midline
50-51	Small fossa medial to mandibular fossa and retroarticular process
52-53	Fossa medial to the paracondylar process (deepest point)
54-55	Distal tip of the paracondylar process
56	Basion, ventral margin of the foramen magnum, midpoint
57-58	Posteriormost tips of the occipital condyles

* adapted from Heck et al., 2019, Balcarcel et al. 2021, Wilson et al., 2021.

S3. WILD VERSUS DOMESTIC ANALYSES

Skull size comparison

ANOVA:
Coefficients:

	Estimate	Std. Error	t value	Pr(>|t|)
(Intercept)	6.29935	0.01734	363.304	<2e-16 ***
classifiers$WDwild	-0.03872	0.03277	-1.182	**0.241**

Residual standard error: 0.1274 on 73 degrees of freedom
Multiple R-squared: 0.01876, Adjusted R-squared: 0.005322
F-statistic: 1.396 on 1 and 73 DF, p-value: 0.2412

Pairwise comparisons using Wilcoxon rank sum test with continuity correction:
domestic
wild 0.058
P value adjustment method: BH

Allometry: regression of shape coordinates on centroid size (CS):

Analysis of Variance, using Residual Randomization
Permutation procedure: Randomization of null model residuals
Number of permutations: 1000
Estimation method: Ordinary Least Squares
Sums of Squares and Cross-products: Type I
Effect sizes (Z) based on F distributions

	Df	SS	MS	Rsq	F	Z	Pr(>F)
CS	1	0.04385	0.043851	**0.09452**	7.6202	5.5139	0.001 **
Residuals	73	0.42009	0.005755	0.90548			
Total	74 0	.46394					

PCA: Main analysis, without brachycephalics (Fig. 3a)

(a) Screeplot:

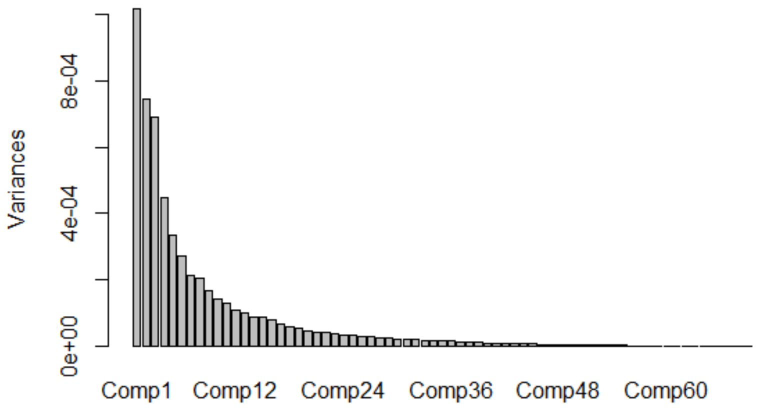

Ordination type: Principal Component Analysis
Centering by OLS mean
Orthogonal projection of OLS residuals
Number of observations: 70
Number of vectors 69
Importance of Components:

	Comp1	Comp2	Comp3	Comp4	Comp5
Eigenvalues	0.001015446	0.0007465222	0.0006913432	0.0004470716	0.0003357793
Proportion of Variance	0.179199424	0.1317414315	0.1220037918	0.0788963178	0.0592561629
Cumulative Proportion	0.179199424	0.3109408559	0.4329446478	0.5118409656	0.5710971285

	Comp6	Comp7	Comp8	Comp9	Comp10
Eigenvalues	0.0002730481	0.0002149519	0.0002049726	0.0001696389	0.0001424864
Proportion of Variance	0.0481857625	0.0379333313	0.0361722490	0.0299367801	0.0251450850
Cumulative Proportion	0.6192828910	0.6572162223	0.6933884713	0.7233252514	0.7484703365

(b) Broken Stick test*: brokenStick (k, 69): 0.04809494*, 6 significant PCs= 62% of the variation.

(c) MANOVA (limited to 6 PCs):

	Df	Pillai	approx F	num Df	den Df	Pr(>F)
classifiers$W_D	1	0.759	33.069	6	63	**< 2.2e-16 *** **
Residuals	68					

(d) Proc.ANOVA (all shape scores):

Analysis of Variance, using Residual Randomization
Permutation procedure: Randomization of null model residuals
Number of permutations: 1000
Estimation method: Ordinary Least Squares
Sums of Squares and Cross-products: Type I
Effect sizes (Z) based on F distributions

	Df	SS	MS	Rsq	F	Z	Pr(>F)
W_D	1	0.04869	0.048685	0.12452	9.6714	5.5788	**0.001 ** **
Residuals	68	0.34231	0.005034	0.87548			
Total	69	0.39099					

(e) Pairwise comparisons using Wilcoxon rank sum test with continuity correction

	domestic
wild	0.75

P value adjustment method: BH

(f) Disparity:

Procrustes variances for defined groups

domestic	wild
0.005708757	0.005298301

Pairwise absolute differences between variances

	domestic	wild
domestic	0.0000000000	0.0004104564
wild	0.0004104564	0.0000000000

P-Values

	domestic	wild
domestic	1.0	0.5
wild	0.5	1.0

PCA: with brachycephalics (for comparison, Fig. 3b)

(a) Screeplot:

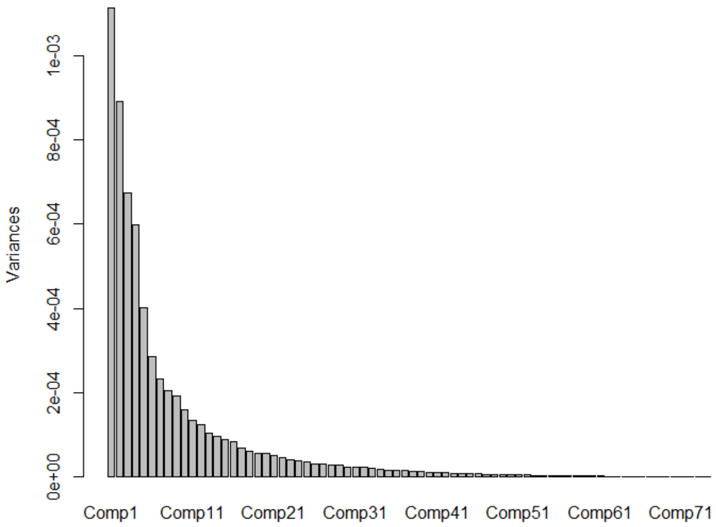

(b) Broken Stick test: brokenStick(k, 74)= 0.04578409, 6 significant PCs=63.2% of variation

(c) MANOVA (limited to 6 PCs):

	Df	Pillai	approx F	num Df	den Df	Pr(>F)
classifiers$WD	1	0.74628	33.336	6	68	< 2.2e-16 ***
Residuals	73					

(d) Proc.ANOVA (all shape scores):

	Df	SS	MS	Rsq	F	Z	Pr(>F)
W_D	1	0.04881	0.048808	0.1052	8.5828	5.4369	0.001 **
Residuals	73	0.41513	0.005687	0			

(e) LDA with mevolCVP:

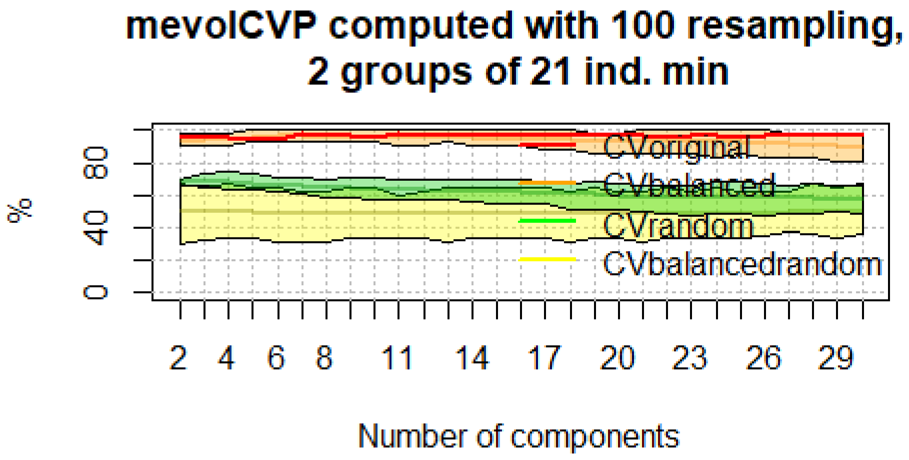

Cross-validation percentages:

	mean-CVbalanced	CI5%-CVbalanced	CI95%-CVbalanced
2PCs	93.59524	90.47619	97.61905
3PCs	94.19048	90.47619	97.61905
4PCs	94.97619	90.47619	97.73810
5PCs	95.88095	92.73810	100.00000
6PCs	96.42857	92.85714	100.00000
**7PCs**	**96.47619**	**92.85714**	**100.00000**
8PCs	96.21429	92.85714	100.00000
9PCs	96.00000	92.85714	100.00000
10PCs	96.04762	92.85714	100.00000
11PCs	95.85714	90.47619	100.00000
12PCs	95.76190	90.47619	100.00000
13PCs	95.54762	92.73810	100.00000
14PCs	95.14286	90.47619	100.00000
15PCs	94.69048	90.47619	100.00000
16PCs	94.52381	90.35714	100.00000
17PCs	94.47619	88.09524	100.00000
18PCs	93.95238	88.09524	100.00000
19PCs	93.19048	85.71429	97.73810
20PCs	93.38095	85.71429	97.73810
21PCs	93.61905	85.71429	100.00000
22PCs	93.76190	85.71429	100.00000
23PCs	93.23810	85.71429	100.00000
24PCs	93.35714	83.33333	100.00000
25PCs	93.09524	85.59524	100.00000
26PCs	92.42857	83.33333	100.00000
27PCs	91.76190	83.21429	97.61905
28PCs	91.02381	83.33333	97.61905
29PCs	90.07143	80.95238	97.61905
30PCs	89.07143	80.95238	97.61905

> zp$posterior (predictive LDA)

	domestic	wild
BZM68636	2.241070e-04	9.997759e-01
BZM68520	5.244450e-06	9.999948e-01

BZM68616	5.003249e-07	9.999995e-01
BZM8999	9.829532e-04	9.990170e-01
BZM68641	2.649352e-05	9.999735e-01
ZIN3820	3.420200e-05	9.999658e-01
ZIN439	3.949192e-01	6.050808e-01
ZIN3806	3.413450e-06	9.999966e-01
ZIN3804	4.872268e-06	9.999951e-01
ZIN1052	2.436095e-01	7.563905e-01
ZIN12479	6.468376e-05	9.999353e-01
ZIN12488	4.863932e-04	9.995136e-01
ZIN447	1.065725e-04	9.998934e-01
ZIN12490	1.811013e-02	9.818899e-01
ZIN6956	3.848645e-05	9.999615e-01
ZIN19042	7.009127e-04	9.992991e-01
ZIN19043	1.943374e-03	9.980566e-01
ZIN19041	1.503425e-03	9.984966e-01
ZIN958	5.151894e-06	9.999948e-01
ZIN18249	8.526007e-03	9.914740e-01
ZIN31215	2.867184e-01	7.132816e-01
Federer1	9.965599e-01	3.440078e-03
Spirito_Florian1	9.999999e-01	1.019003e-07
Florian_1	1.000000e+00	1.266335e-08
Florian_2	9.959204e-01	4.079590e-03
Florian_3	9.999947e-01	5.300118e-06
PIM10892	9.997997e-01	2.002614e-04
PIM10882	9.998081e-01	1.919353e-04
PIM10880	9.971725e-01	2.827452e-03
PIM10888	9.999988e-01	1.218906e-06
Venezuela_1237	9.801996e-01	1.980042e-02
Venezuela2_1236	9.980580e-01	1.942017e-03
K19506	9.999990e-01	9.721112e-07
K7558	9.775529e-01	2.244712e-02
NMW18639	9.966908e-01	3.309198e-03
NMW2069	7.459222e-01	2.540778e-01
NMW2559	9.999961e-01	3.861236e-06
Bern314	9.999990e-01	1.035997e-06
Bern301	9.999989e-01	1.074285e-06
K1556	9.991399e-01	8.600729e-04
K20591	9.999636e-01	3.640761e-05
K21179	9.999701e-01	2.988741e-05
K19238	9.999918e-01	8.155953e-06
K19235	9.998949e-01	1.051107e-04
K22336	9.999998e-01	2.066462e-07
K18059	**8.089362e-01**	**1.910638e-01**
Bern316	9.989676e-01	1.032359e-03
PIM10883	9.993597e-01	6.402993e-04
PIM10895	9.996512e-01	3.487668e-04
PIM10881	9.999979e-01	2.122082e-06
PIM10891	9.877116e-01	1.228840e-02
PIM10884	9.992473e-01	7.527361e-04
Naters1_Mayara	9.999996e-01	3.986625e-07
Naters_1568766	9.996905e-01	3.094717e-04
Aargau2_Elsa	9.998747e-01	1.252654e-04
Kaempf2_Breuninger	9.998133e-01	1.867108e-04
Kaempf1	9.999999e-01	1.350104e-07
PIM10893	9.994452e-01	5.548376e-04
PIM10903	9.852196e-01	1.478040e-02

PIM10879	9.998520e-01	1.480345e-04
K1438	9.995390e-01	4.609516e-04
K30447	9.995814e-01	4.186012e-04
Naters3	9.967423e-01	3.257693e-03
Naters_1936203	9.999996e-01	4.463296e-07
Naters_1666055	9.999787e-01	2.125287e-05
Naters_1666023	9.999840e-01	1.599298e-05
K15442	1.000000e+00	3.597017e-09
K18719	9.999985e-01	1.502733e-06
K28513	9.999991e-01	8.729161e-07
K28514	9.999944e-01	5.617073e-06

(f) Disparity:

Randomized Residual Permutation Procedure Used
1000 Permutations

Procrustes variances for defined groups

domestic	wild
0.006511389	0.005348637

Pairwise absolute differences between variances

	domestic	wild
domestic	0.000000000	0.001162752
wild	0.001162752	0.000000000

P-Values

	domestic	wild
domestic	1.000	**0.171**
wild	0.171	1.000

Domestication trends: rostral shortening and tooth row length reduction tests

(a) TEST for rostral shortening:

Pairwise comparisons using Wilcoxon rank sum exact test
data: data$rost and classifiers$W_D

	domestic
wild	0.073

P value adjustment method: BH

(b) TEST for tooth row length reduction:

Pairwise comparisons using Wilcoxon rank sum exact test
data: data$rowl and classifiers$W_D

	domestic
wild	1.7e-06

P value adjustment method: BH

Size effect on PC1: regression of PC1 scores on centroid size (CS):

	Df	SS	MS	Rsq	F	Z	Pr(>F)
CS	1	0.020275	0.0202752	0.28937	27.69	3.8324	0.001 **
Residuals	68	0.049791	0.0007322	0.71063			
Total	69	0.070066					

Signif. codes: 0 '***' 0.001 '**' 0.01 '*' 0.05 '.' 0.1 ' ' 1

Call: procD.lm(f1 = shape ~ CS, iter = 999, data = gdf4)

S4. Breed analysis includes only populations with at least 3 members plus all brachycephalic specimens (total n=42).

Skull size comparison.

Pairwise comparisons using Wilcoxon rank sum exact test

	Capra_grigia	Chamois	Mamberziege	Pygmy	Saanen	Stiefelgeisse	Thebener/Zaraibi	Toggenburger	Valais_Blackneck
Chamois	0.390	-	-	-	-	-	-	-	-
Mamberziege	0.556	0.600	-	-	-	-	-	-	-
Pygmy	**0.038**	**0.055**	0.469	-	-	-	-	-	-
Saanen	0.865	0.865	0.556	**0.038**	-	-	-	-	-
Stiefelgeissziege	1.000	0.918	0.682	0.075	1.000	-	-	-	-
Thebener/Zaraibi	0.075	0.099	0.600	0.091	0.075	0.151	-	-	-
Toggenburger	0.306	0.600	0.682	0.075	0.878	0.865	0.151	-	-
Valais_Blackneck	0.710	0.682	0.514	**0.038**	0.878	1.000	0.071	0.865	-
White_goat	0.257	0.151	0.878	**0.055**	0.159	0.257	0.391	0.151	0.078

P value adjustment method: BH

Allometry: regression of shape coordinates on centroid size (CS):

Analysis of Variance, using Residual Randomization
Permutation procedure: Randomization of null model residuals
Number of permutations: 1000
Estimation method: Ordinary Least Squares
Sums of Squares and Cross-products: Type I
Effect sizes (Z) based on F distributions

	Df	SS	MS	Rsq	F	Z	Pr(>F)
CS	1	0.040544	0.040544	**0.15535**	7.3567	4.52	0.001 **
Residuals	40	0.220444	0.005511	0.84465			
Total	41	0.260988					

PCA: Breed analysis.

(a) Screeplot.

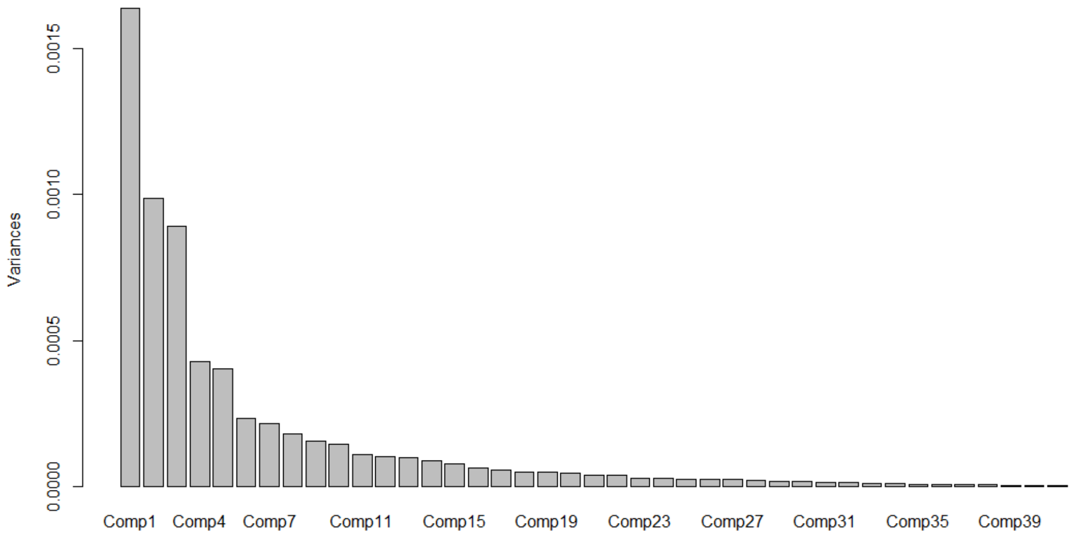

Supplementary figure: PCA: Breed with size-normalized scores

(b) PCA, size-normalized shape components:

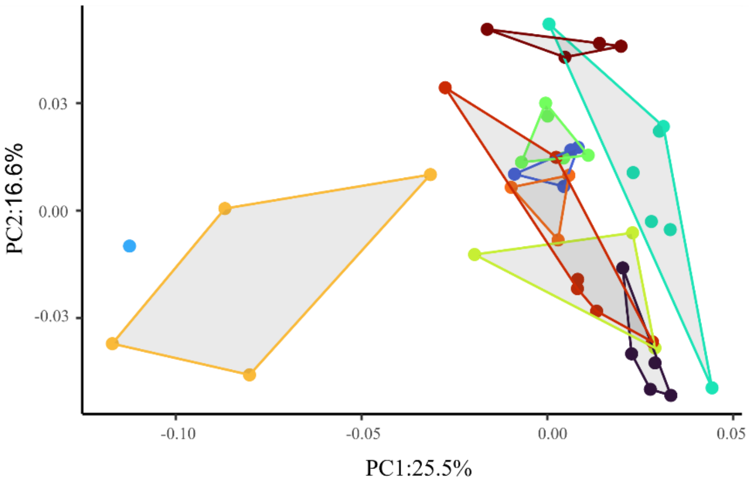

S5. Specimens in Figure 6A-K. Inst. abbreviations: NHMB = Naturhistorisches Museum Bern; ZIN: Zoological Museum of the Zoological Institute of the Russian Academy of Sciences; ZMB Mamm: Nehring Collection, Zoologische Sammlung der Königlichen Landwirtschaftlichen Hochschule zu Berlin, Museum für Naturkunde Berlin; ZMUZH: Zoological Museum of the University of Zurich; MdH: Musee de l'Homme, Paris,

Plate 1	*Genus*	*species*	Sex	wild/dom	breed/population	Museum/Collection	Field/Museum ID	locality
A	*Capra*	*hircus*	m	domestic	unknown	ZMB Mamm.	A14011	na
B	*Capra*	*hircus*	f	domestic	Sempione	NHMB	103848	Naters, Brig, private farm
C	*Capra*	*hircus*	f	domestic	Valais Blackneck	NHMB	103860	Naters, Brig, private farm
D	*Capra*	*hircus*	na	domestic	unknown	ZMUZH	ZM17744	
E	*Capra*	*aegagrus*	m	wild	na	ZIN	ZIN12488	Asia Minor
F	*Capra*	*hircus*	m	domestic	Capra grigia	NHMB	103843	Switzerland
G	*Capra*	*hircus*	m	domestic	Pfauenziege or "Taubenziege"	NHMB	103855	Althus, Ebersecken, private farm
H	*Capra*	*hircus*	m	domestic	unknown	ZMB Mamm.	#6658	na
I	*Capra*	*hircus*	na	domestic	unknown	MdH	AF0630	na
J	*Capra*	*hircus*	na	domestic	unknown	ZMB Mamm.	#6709	na
K	*Capra*	*hircus*	m	domestic	Sempione	private farm	na	Naters, Brig, private farm

Supplementary S6_RScript_Main
author: "Ana Balcarcel"
output: word_document

```
install.packages("devtools")
install.packages("tidyverse")
install.packages("readr")
install.packages("readxl")
install.packages("writexl")
install.packages("dbplyr")
install.packages("ggfortify")
install.packages("ggplot2")
install.packages("geomorph")
install.packages("Morpho")
install.packages("rgl")
install.packages("car")
install.packages("Rcpp")
install.packages("PerformanceAnalytics")
install.packages("knitr")
install.packages("MASS")
install.packages("plyr")
install.packages("dplyr")
install.packages("viridis")
install.packages("PCDimension")
install.packages("cowplot")
devtools::install_github("vbonhomme/mevolCVP")
install.packages("knitr")

library(devtools)
library(tidyverse)
library(readr)
library(readxl)
library(writexl)
library(ggfortify)
library(ggplot2)
library(Morpho)
library(ggplot2)
library(devtools)
library(Morpho)
library(rgl)
library(RRPP)
library(car)
library(Rcpp)
library(PerformanceAnalytics)
library(knitr)
library(MASS)
library(dplyr)
library(plyr)
```

```
library(dbplyr)
library(geomorph)
library(cowplot)
library(viridis)
library(PCDimension)
library(mevolCVP)
library(knitr)

#DATA
data = read_excel("S1_Data for Rscript.xlsx")
data= data[,1:182]
table(data$W_D)
table(data$breed_variety)
#REMOVE BRACHYCEPHALICS
data= filter(data, breed_variety != "Thebener/Zaraibi")
data= filter(data, breed_variety != "Mamberziege")
#LANDMARKS AND CLASSIFIERS
p= 58
k= 3
n= 70
A=(data[1:70,9:182])
classifiers <- data[1:70,1:8]
landmarks= arrayspecs(A,p,k, sep = NULL)
landmark.pairs= read_excel("PairedLandmarks_goats2.xlsx")
landmark.pairs=as.matrix(landmark.pairs)
#GPA
gdf=geomorph.data.frame(shape= landmarks, ind= classifiers$ID)
gpa=gpagen(gdf$shape)

#BOXPLOT Skull size:
boxplot(log10(gpa$Csize)~classifiers$W_D, col=c("darkgrey ","black "), ylab="Skull size", xlab = "",
medcolor="white") #main= "Skull size: wild vs domestic"
#stats size
Z=lm(log(gpa$Csize)~ classifiers$W_D)
summary(Z)
#non-parametric:
pairwise.wilcox.test(log10(gpa$Csize),classifiers$W_D, p.adjust.method = 'BH')

#dataframe
gdf2=geomorph.data.frame(shape= gpa$coords, CS= gpa$Csize, ind= classifiers$ID,
W_D=classifiers$W_D, breed_variety=classifiers$breed_variety, region=classifiers$region)
#Symmetric component
X=bilat.symmetry(shape, ind, object.sym = TRUE, land.pairs = landmark.pairs,iter=999, RRPP=TRUE,
data=gdf2)
#dataframe for PCA
gdf3=geomorph.data.frame(shape= X$symm.shape, CS= gpa$Csize, ind= classifiers$ID,
W_D=classifiers$W_D,  breed_variety=classifiers$breed_variety, region=classifiers$region,
use=classifiers$selection)
```

```
#PCA:
P1=gm.prcomp(X$symm.shape)
summary(P1)
#ggplot dataframe
DF_pca<-data.frame(CS=gpa$Csize, P1$x,  ind=classifiers$ID, W_D=classifiers$W_D,
breed_variety=classifiers$breed_variety, region= classifiers$region, use= classifiers$selection)
table(DF_pca$W_D)
#convex hulls
find_hull1 <- function(df) df[chull(df$Comp1, df$Comp2), ]
hulls1 <- ddply(DF_pca, "W_D", find_hull1)

Plot1= ggplot(DF_pca, aes(x=Comp1,y=Comp2,col=W_D, shape= W_D))+
  geom_point(size=3)+
  scale_color_manual(values = c("darkgrey","black"))  +
  theme_classic() + geom_polygon(data=hulls1, alpha=0.1) +
  labs(title = "PCA")
Plot1

#allometry
Reg= procD.lm(shape ~CS, iter = 999, data=gdf3)
summary(Reg)
plot(Reg)
#Size effect along PC1 only
gdf4=geomorph.data.frame(shape= P1$x[,1], CS= gpa$Csize, ind= classifiers$ID,
W_D=classifiers$W_D,  breed_variety=classifiers$breed_variety, region=classifiers$region,
use=classifiers$selection)
Reg2= procD.lm(shape ~CS, iter = 999, data=gdf4)
summary(Reg2)
plot(Reg2)

#PCdimensions:
qqPlot(P1$x)
screeplot(P1, npcs=69)
brokenStick(k, 69)
spca <- SamplePCA(t(P1$x))
bsDimension(spca, FUZZ = 0.005)

#PCA stats:
summary(manova(P1$x[,1:6]~classifiers$W_D))
m= procD.lm(shape ~W_D, iter=999, data=gdf3)
summary(m)
pairwise.wilcox.test(P1$x, DF_pca$W_D, p.adjust.method = 'BH')

#lda with mevol:
Y=mevol_CVP(P1$x, group = classifiers$W_D, nrep=100, return.matrix = F, print.legend = T)
summary(Y)
Y$CVsummary
#predictive lda (mevol package):
```

```
set.seed(5)
predict_ids <- sample(nrow(P1$x), 70, replace=FALSE)
Y2=pldam(mat=P1$x, group=classifiers$W_D,
  mattoid=P1$x[predict_ids, ], codetoid=paste0("id_", 1:nrow(P1$x[predict_ids, ])),
  ncpts = 7)
 Y2$matplda
#regular LDA:
z<-lda(P1$x[,1:7],classifiers$W_D, CV=F) #false if only 2 groups!
zp<-predict(z, P1$x[,1:7])
zp$posterior
summary(zp)
#plot LDA
lda.df <- data.frame(LD1 = zp$x, class = zp$class) #only one LD axis
ggplot(lda.df) + geom_density(aes(LD1, fill = class), alpha = 0.2)

#DISPARITY:
disp= morphol.disparity(P1$x ~ 1, groups= ~W_D, data = gdf3, iter=999, print.progress = FALSE)
summary(disp)
disp$PV.dist.Pval

#WIREFRAMES for PCA PLOT
#MEAN shape
ref<- mshape(gpa$coords)
links=87
cranial.links <- matrix(data=c(1,2, 1,3, 3,4, 2,4, 4,11, 3,10, 1,35, 2,35, 10,9, 11,9, 9,30, #11
   30,31, 31,34, 31,32, 31,33, 32,28, 33,29, 28,26, 29,27, 26,20, 27,21,
  20,18, 21,19, 19,23, 18,22, 9,22, 9,23, 14,12, 15,13, 12,18, 13,19,
 12,7, 13,8, 5,7, 6,8, 25,41, 41,39, 39,37, 37,35, 35,42, 42,43, 43,44,
24,40, 40,38, 38,36, 36,35, 25,44, 24,44,
49,48, 49,47, 47,50, 48,51, 51,53, 50,52, 52,54, 53,55,
56,53, 56,52, 34,56, 58,57, 21,22, 20,21, 26,27, 28,29, 32,33, 6,7, 5,6,
30,32, 30,33, 55,54, 55,48, 54,47, 30,16, 30,17, 16,10, 17,22, 51,49,
50,49, 31,58, 32,57, 12,13, 9,16, 9,17, 24,54, 25,55, 40,45, 41,46 ),nrow=links,ncol=2,byrow=TRUE)

#PlotreftoTarget graphing parameters:
GP= gridPar(pt.bg = "black", pt.size =1, link.col = "black",
        link.lwd = 0, link.lty = 1, out.col = "black", out.cex = 0.1,
        tar.pt.bg = "darkgrey", tar.pt.size = 1, tar.link.col = "darkgrey",
        tar.link.lwd = 3, tar.link.lty = 6, tar.out.col = "darkgrey",
        tar.out.cex = 0.1, n.col.cell = 20, txt.adj = 1.5, txt.pos = 1,
        txt.cex = 0.1, txt.col = "black")
#PC1min:
plotRefToTarget(ref, P1$shapes$shapes.comp1$min, method="vector", gridPar= GP, mesh=NULL,
links=cranial.links, label= T, mag=3, axes=F)
#PC1max:
plotRefToTarget(ref, P1$shapes$shapes.comp1$max, method="vector", gridPar= GP, mesh=NULL,
links=cranial.links, label= T, mag=3, axes=F)
#PC2min:
```

```
plotRefToTarget(ref, P1$shapes$shapes.comp2$min, method="vector", gridPar= GP, mesh=NULL,
links=cranial.links, label= T, mag=3, axes=F)
#PC2max:
plotRefToTarget(ref, P1$shapes$shapes.comp2$max, method="vector", gridPar= GP, mesh=NULL,
links=cranial.links, label= T, mag=3, axes=F)


#DOMESTICATION TRENDS
#Rostral length (RL)= nasion to 35
#Tooth Row (TRL)= 38 to 40
#BL (braincase length)= 49 to 56 (basion)
data=mutate(data, RL=log10(sqrt((x9-x35)^2 +(y9-y35)^2 + (z9-z35)^2)), TRL= log10(sqrt((x38-x40)^2
+(y38-y40)^2 + (z38-z40)^2)), BL= log10(sqrt((x49-x56)^2 +(y49-y56)^2 + (z49-z56)^2)))
data=mutate(data, rost=RL/BL, rowl=TRL/BL)
#Boxplot relative rostral length (RL//BL):
boxplot(data$rost~classifiers$W_D, col=c("darkgrey ","black "), ylab="Rostral length", xlab = "",
medcolor="white", las=1) #main= "Skull size: wild vs domestic"
pairwise.wilcox.test(data$rost,classifiers$W_D, p.adjust.method = 'BH')
#Boxplot relative tooth row length (TRL/BL):
boxplot(data$rowl~classifiers$W_D, col=c("darkgrey ","black "), ylab="Tooth row length", xlab = "",
medcolor="white", las=1) #main= "Skull size: wild vs domestic"
pairwise.wilcox.test(data$rowl,classifiers$W_D, p.adjust.method = 'BH')
```

S7_Rscript_Breeds

```
library(devtools)
library(tidyverse)
library(readr)
library(readxl)
library(ggfortify)
library(ggplot2)
library(Morpho)
library(ggplot2)
library(devtools)
library(Morpho)
library(shapes)
library(plot3D)
library(rgl)
library(RRPP)
library(car)
library(Rcpp)
library(PerformanceAnalytics)
library(shiny)
library(knitr)
library(MASS)
library(dplyr)
library(plyr)
library(dbplyr)
library(geomorph)
library(caper)
library(cowplot)
library(viridis)
library(PCDimension)


data2 = read_excel("S1_Data for Rscript.xlsx")
data2= data[,1:182]
data2= filter(data2, breed_variety%in%  c("Thebener/Zaraibi","Mamberziege",
                        "Capra_grigia",
                        "Chamois","Pygmy","Stiefelgeiss",
                        "Toggenburger", "Saanen", "White_goat",
                        "Valais_Blackneck"))
table(data2$W_D)
count(data2$breed_variety)
p= 58
k= 3
n=42
A=(data2[1:42,9:182])
classifiers <- data2[1:42,1:8]
landmarks= arrayspecs(A,p,k, sep = NULL)
landmark.pairs= read_excel("PairedLandmarks_goats2.xlsx")
```

```
landmark.pairs=as.matrix(landmark.pairs)

gdf=geomorph.data.frame(shape= landmarks, ind= classifiers$ID)
gpa=gpagen(gdf$shape)
outliers <- plotOutliers(gpa$coords)

gdf2=geomorph.data.frame(shape= gpa$coords, CS= gpa$Csize, ind= classifiers$ID,  species=
classifiers$species, breed=classifiers$breed_variety,  region=classifiers$region)
X=bilat.symmetry(shape, ind, object.sym = TRUE, land.pairs = landmark.pairs,iter=999, RRPP=TRUE,
data=gdf2)
gdf3=geomorph.data.frame(shape= X$symm.shape, CS= gpa$Csize, ind= classifiers$ID,
W_D=classifiers$W_D, species= classifiers$species, breed=classifiers$breed_variety,
region=classifiers$region, cva_pop=classifiers$CVA_pop)

Reg= procD.lm(shape ~CS, iter = 999, data=gdf3)
summary(Reg)
plot(Reg)

#ANOVA
Z=lm(log(gpa$Csize)~ classifiers$breed_variety)
summary(Z)
pairwise.wilcox.test(log10(gpa$Csize),classifiers$breed_variety, p.adjust.method = 'BH')
boxplot(log10(gpa$Csize)~classifiers$breed_variety,
        col=c( "darkgrey", "darkgrey", "darkgrey", "darkgrey","darkgrey", "darkgrey","darkgrey",
             "darkgrey", "darkgrey"),
        ylab="log10 Centroid size", xlab="", medcolor="white", las=2)

#PCA Breeds
P2= gm.prcomp(gdf3$shape)
summary(P2)
P2.df<-data.frame(CS=gpa$Csize, P2$x, ind=classifiers$ID, W_D=classifiers$W_D,
breed=classifiers$breed_variety, region= classifiers$region, selection= classifiers$selection,
cva_pop=classifiers$CVA_pop)
table(P2.df$breed)
table(P2.df$cva_pop)

find_hull2 <- function(df) df[chull(df$Comp1, df$Comp2), ]
hulls2 <- ddply(P2.df, "breed", find_hull2)

Plot2= ggplot(P2.df, aes(x=Comp1,y=Comp2,col=breed))+
  geom_point(size=1.5)+ labs(title = "PCA: breeds/populations")+
scale_color_viridis(option= "turbo", discrete=TRUE) +
theme_classic() + geom_polygon(data=hulls2, alpha=0.1) + geom_polygon(data=hulls2, alpha=0.1)
Plot2

#NORMALality
qqPlot(P2$x)
screeplot(P2, npcs=41)
```

```
#Broken stick:
brokenStick(k, 41)
spca <- SamplePCA(t(P2$x))
bsDimension(spca, FUZZ = 0.005)
#Manova:
summary(manova(P2$x[,1:5]~classifiers$breed_variety))
#procrustes ANOVA
m= procD.lm(shape ~breed, iter=999, data=gdf3)
summary(m)
#pairwise:
pairwise.wilcox.test(P2$x, P2.df$breed, p.adjust.method = 'BH')

#DISPARITY
disp= morphol.disparity(P2$x ~ 1, groups= ~breed, data = gdf3, iter=999, print.progress = FALSE)
summary(disp)
disp$PV.dist.Pval

#WIREFRAMES
ref<- mshape(gpa$coords)
links=87
cranial.links <- matrix(data=c(1,2, 1,3, 3,4, 2,4, 4,11, 3,10, 1,35, 2,35, 10,9, 11,9, 9,30,
                    30,31, 31,34, 31,32, 31,33, 32,28, 33,29, 28,26, 29,27, 26,20, 27,21,
                    20,18, 21,19, 19,23, 18,22, 9,22, 9,23, 14,12, 15,13, 12,18, 13,19,
                    12,7, 13,8, 5,7, 6,8, 25,41, 41,39, 39,37, 37,35, 35,42, 42,43, 43,44,
                    24,40, 40,38, 38,36, 36,35, 25,44, 24,44,
                    49,48, 49,47, 47,50, 48,51, 51,53, 50,52, 52,54, 53,55,
                    56,53, 56,52, 34,56, 58,57, 21,22, 20,21, 26,27, 28,29, 32,33, 6,7, 5,6,
                    30,32, 30,33, 55,54, 55,48, 54,47, 30,16, 30,17, 16,10, 17,22, 51,49,
                    50,49, 31,58, 32,57, 12,13, 9,16, 9,17, 24,54, 25,55, 40,45, 41,46
),nrow=links,ncol=2,byrow=TRUE)

#PlotreftoTarget graphing parameters:
GP= gridPar(pt.bg = "black", pt.size =1, link.col = "black",
      link.lwd = 0, link.lty = 1, out.col = "black", out.cex = 0.1,
      tar.pt.bg = "darkgrey", tar.pt.size = 1, tar.link.col = "darkgrey",
      tar.link.lwd = 3, tar.link.lty = 6, tar.out.col = "darkgrey",
      tar.out.cex = 0.1, n.col.cell = 20, txt.adj = 1.5, txt.pos = 1,
      txt.cex = 0.1, txt.col = "black")

#PC1min:
plotRefToTarget(ref, P2$shapes$shapes.comp1$min, method="vector", gridPar= GP, mesh=NULL,
links=cranial.links, label= T, mag=3, axes=F)
#PC1max:
plotRefToTarget(ref, P2$shapes$shapes.comp1$max, method="vector", gridPar= GP, mesh=NULL,
links=cranial.links, label= T, mag=3, axes=F)
#PC2min:
plotRefToTarget(ref, P2$shapes$shapes.comp2$min, method="vector", gridPar= GP, mesh=NULL,
links=cranial.links, label= T, mag=3, axes=F)
```

```
#PC2max:
plotRefToTarget(ref, P2$shapes$shapes.comp2$max, method="vector", gridPar= GP, mesh=NULL,
links=cranial.links, label= T, mag=3, axes=F)

#PCA breeds (residuals)
P4= gm.prcomp(Reg$residuals)
summary(P4)
DF_P4<-data.frame(CS=gpa$Csize, P4$x,  ind=classifiers$ID, W_D=classifiers$W_D,
breeds=classifiers$breed_variety, region= classifiers$region, use= classifiers$selection)
table(DF_P4$W_D)

find_hull1 <- function(df) df[chull(df$Comp1, df$Comp2), ]
hulls1 <- ddply(DF_P4, "breeds", find_hull1)

Plot4= ggplot(DF_P4, aes(x=Comp1,y=Comp2,col=breeds))+
  geom_point(size=2)+
  scale_color_viridis(option = "turbo", discrete = T)  + #geom_text(aes(label="breeds") +
  theme_classic() + geom_polygon(data=hulls1, alpha=.1) +
  labs(title= "PCA breeds (size-normalized shape scores")
Plot4
```

1	2
3	4
5	6
7	8
10	11
12	13
14	15
16	17
18	19
20	21
22	23
24	25
26	27
28	29
32	33
36	37
38	39
40	41
45	46
47	48
50	51
52	53
54	55
57	58
